# [4 + 2] Cycloaddition of α-bromotrifluoromethylhydrazone with alkenes: synthesis of trifluoromethyltetrahydropyridazines†

**DOI:** 10.1039/d5ra03000e

**Published:** 2025-06-09

**Authors:** Yanhui Zhao, Hemin Rong, Khurshed Bozorov, Xueqing Zhang, Buer Song, Wei Liu

**Affiliations:** a Xinjiang Key Laboratory of Clean Conversion and High Value Utilization of Biomass Resources, School of Chemistry and Chemical Engineering, Yili Normal University Xinjiang Yining 835000 China zhxq0612@163.com ucasliuwei@126.com songbuer20211023@163.com; b Institute of Biochemistry, Samarkand State University University Blvd. 15 Samarkand 140104 Uzbekistan

## Abstract

A catalyst-free [4 + 2] cyclization process between trifluoromethyl-containing 1,2-diazabuta-1,3-diene and simple olefins was developed by *in situ* generation. Under mild conditions, trifluoromethyl-containing 1,4,5,6-tetrahydropyridazine compounds were obtained, in high yields (up to 96% yields).

## Abstract 2

This paper reports a mild, catalyst-free [4 + 2] cycloaddition of *in situ*-generated trifluoromethyl 1,2-diazabuta-1,3-dienes with alkenes to synthesize trifluoromethyl tetrahydropyridazines.

## Graphical abstract

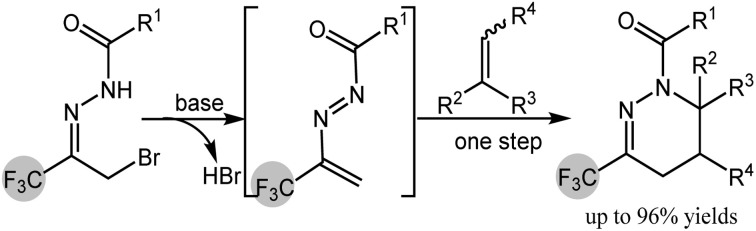

1,4,5,6-Tetrahydropyrazines^1^ are important six-membered nitrogen heterocycles that are widely found in numerous natural products. They also serve as structural subunits in various bioactive molecules and drugs, such as the antihypertensive hydralazine, dihydralazine, and endralazine, as well as the antidepressant drug piperazine.^2^

In addition, the introduction of trifluoromethyl groups (CF_3_) into drug molecules can significantly improve the physical and chemical properties, metabolic stability, and drug activity of drug molecules.^3^ Therefore, CF_3_ plays an important role in medicine, pesticides, and materials. So far, the direct introduction of trifluoromethylation using trifluoromethylation reagents has been well developed.^4^ The synthesis of trifluoromethylated organic molecules using trifluoromethylation building blocks is equally attractive and important as another important approach.^5^

At the same time, by consulting the literature, it was found that α-bromoacylhydrazone can generate 1,2-diazabuta-1,3-diene *in situ* under the action of alkali, and can undergo [4 + 1]^6^ cycloaddition, [4 + 2]^7^ cycloaddition and [4 + 3]^8^ cycloaddition with dienophiles to prepare biologically active nitrogen heterocyclic compounds. Therefore, in recent years, α-bromoacylhydrazone has been widely used in organic synthesis as a diene precursor. For example, in 2012, Bolm's group reported the asymmetric [4 + 1] cycloaddition of 1,2-diazabuta-1,3-diene *in situ* generated by α-haloacylhydrazone with a sulfur ylide, catalyzed by copper trifluoromethanesulfonate and Tol-BINAP, a series of dihydropyrazole compounds were obtained in up to 97% yield and 94% enantioselectivity (Scheme 1a).^9^ In 2015, Luo's group performed the [4 + 2] cycloaddition of α-haloacylhydrazone to 1,2-diazabuta-1,3-diene with simple olefins, especially ethylene, and obtained 1,4,5,6-tetrahydropyridazine compounds in up to 99% yield (Scheme 1b).^10^ In 2016, Zhao's group obtained 1,2,4,5-oxatriazepines from the [4 + 3] cycloaddition of α-halogenated acylhydrazones with nitrones in the presence of sodium carbonate (Scheme 1c).^11^

**Scheme 1:**
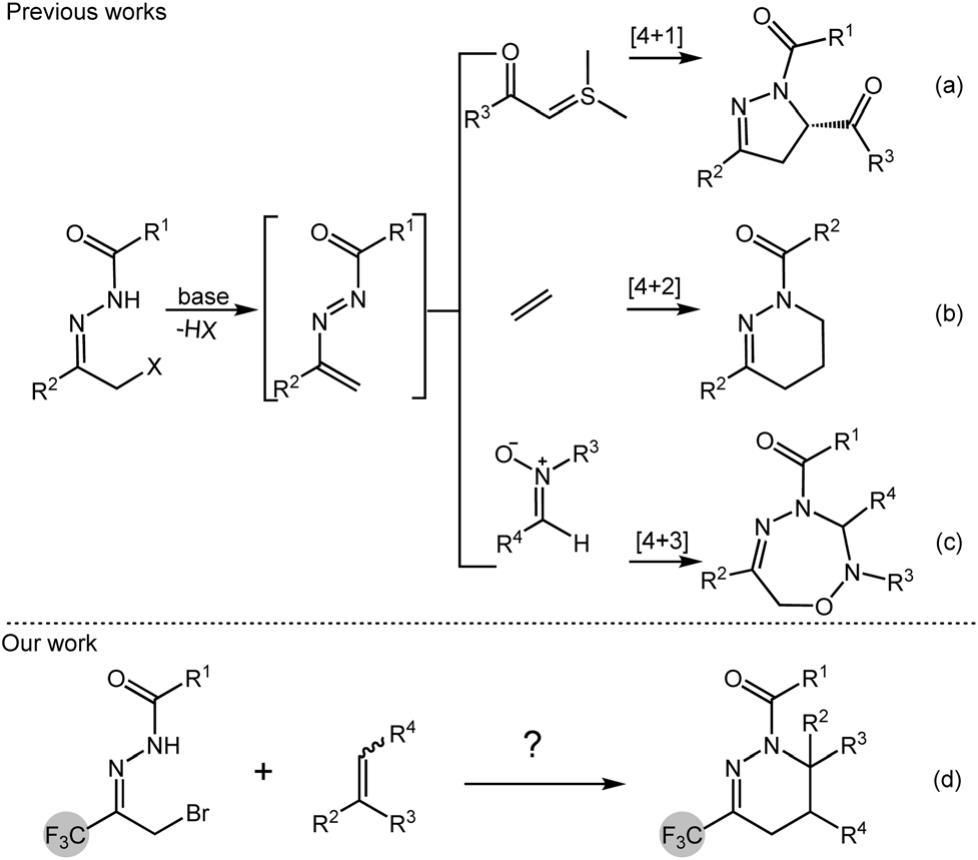
Selected examples of α-bromoacylhydrazones participated cycloaddition reactions.

Based on the above research, a catalyst-free [4 + 2] cyclization process between trifluoromethyl-containing 1,2-diazabuta-1,3-diene and simple olefins was developed by *in situ* generation. Under mild conditions, trifluoromethyl-containing 1,4,5,6-tetrahydropyridazine compounds were obtained (Scheme 1d).

Initially, α-bromotrifluoromethyl acylhydrazone 1a (1.0 equiv.), styrene 2a (3.0 equiv.) and K_2_CO_3_ (2.0 equiv.) were reacted in dichloromethane at room temperature to give the target compound 3a in 64% yield (Table 1, entry 1). To further improve the yield of the target product, the reaction conditions were optimized in terms of solvents, bases, and material ratios, and representative results are summarized in Table 1. When we studied the effect of different bases on the reaction, such as Cs_2_CO_3_, compound 3a was obtained in 25% yield (Table 1, entry 2). When the base is Na_2_CO_3_, compound 3a was obtained in 83% yield (Table 1, entry 3). At the same time, we also investigated the effect of organic base Et_3_N on the reaction, and obtained compound 3a in 16% yield (Table 1, entry 4). Therefore, we use Na_2_CO_3_ as the optimal alkali. Subsequently, we optimized the material ratio of the reaction (Table 1, entry 5–8). The results showed that the appropriate molar ratio of 1a/2a/Na_2_CO_3_ was 1/3/2 (Table 1, entry 3). Finally, we optimized the effects of different solvents on the reaction. We explored the effects of THF, CH_3_CN, and MeOH on the reaction (Table 1, entry 9–11). The results show that the optimal solvent is CH_2_Cl_2_.

**Table 1:** Optimization of additive and temperature^a^

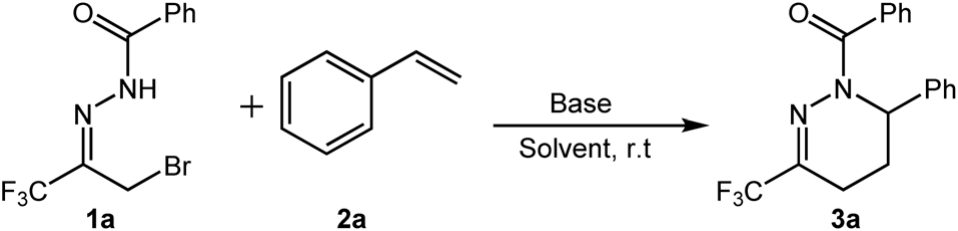				
Entry	Molar ratio of 1a/2a/base	Base	Solvent	Yield^b^ (%)
1	1 : 3 : 2	K_2_CO_3_	CH_2_Cl_2_	64
2	1 : 3 : 2	Cs_2_CO_3_	CH_2_Cl_2_	25
3	1 : 3 : 2	Na_2_CO_3_	CH_2_Cl_2_	83
4	1 : 3 : 2	Et_3_N	CH_2_Cl_2_	16
5	1 : 2 : 2	Na_2_CO_3_	CH_2_Cl_2_	66
6	1 : 1.5 : 2	Na_2_CO_3_	CH_2_Cl_2_	50
7	1 : 2 : 2.5	Na_2_CO_3_	CH_2_Cl_2_	52
8	1 : 2 : 1.5	Na_2_CO_3_	CH_2_Cl_2_	66
9	1 : 2 : 2	Na_2_CO_3_	THF	19
10	1 : 2 : 2	Na_2_CO_3_	CH_3_CN	28
11	1 : 2 : 2	Na_2_CO_3_	MeOH	N. R.

a All reactions were carried out by using 0.2 mmol of 1a, 3 eq. of 2a and 2 eq. of base in 3 mL of solvent.

b Isolated yields.

Under the optimized conditions, the substrate suitability of this transformation reaction was further investigated. The results are shown in Scheme 2.

**Scheme 2:**
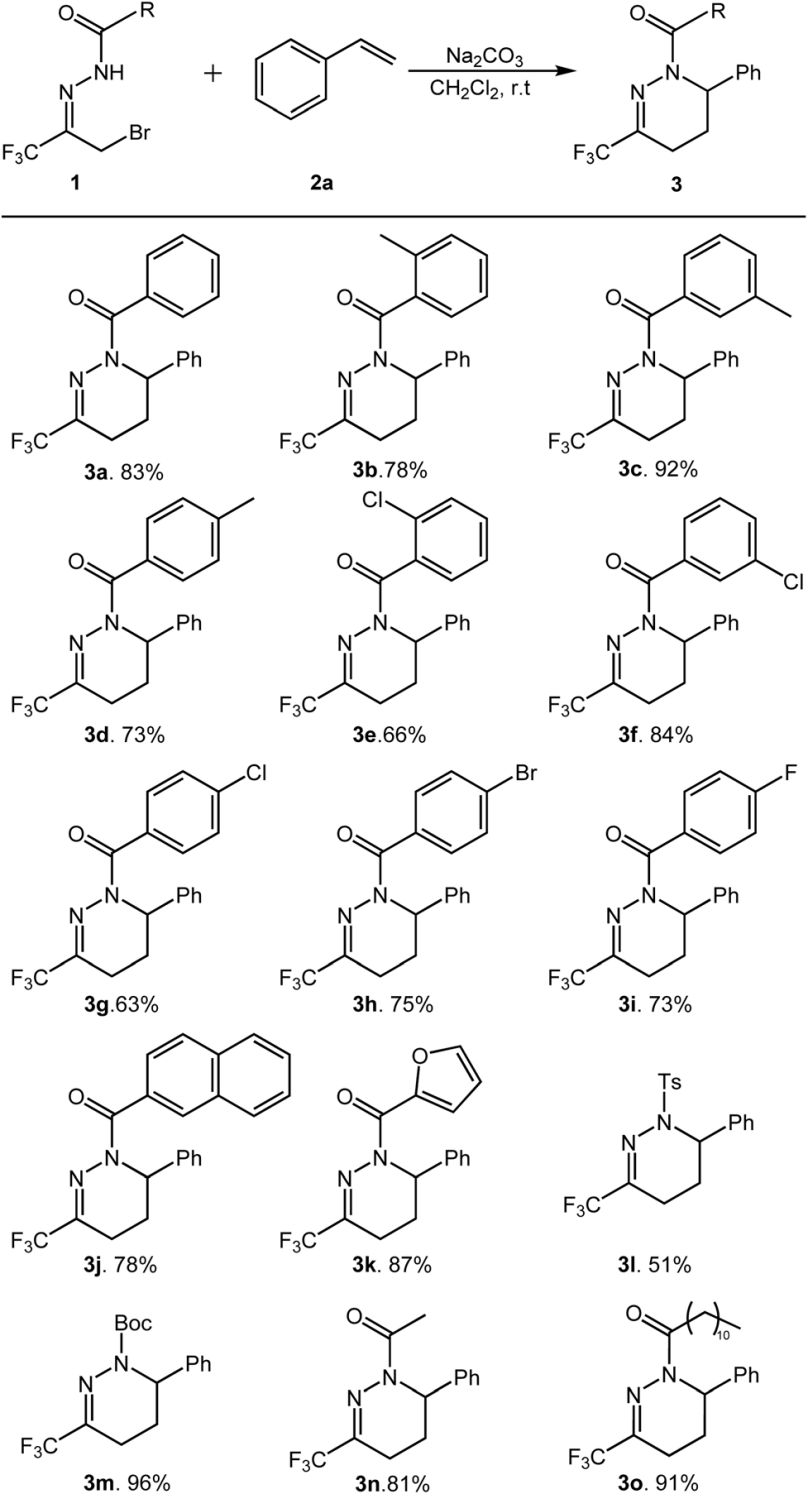
Substrate scope of hydrazones^*a*,*b*^. ^*a*^All reactions were carried out by using 0.2 mmol of 1, 3 eq. of 2a and 2 eq. of Na_2_CO_3_ in 3 mL of CH_2_Cl_2_. ^*b*^Isolated yields.

Firstly, the reaction adaptability of substrate 1 was studied, and the effect of R^1^ substituent on substrate activity was investigated. Various substituted α-bromotrifluoromethyl acylhydrazones can efficiently generate the target products in moderate to good yields (Scheme 2, 3a–3o). The results indicate that the reaction proceeds efficiently when the R^1^ group is either aromatic or aliphatic, affording the corresponding products in good yields. Notably, for aromatic R^1^ groups, the electronic characteristics of substituents on the phenyl ring exhibit minimal influence on product yield, whereas the positional isomerism of substituents on the phenyl ring demonstrates a pronounced effect on yield. For example, when R^1^ was the *o*-/*m*-/*p*-methylphenyl (3b–3d), the *m*-methylphenyl product (3c), was obtained with the highest yield. When R^1^ is *o*-/*m*-/*p*-chlorophenyl, *m*-chlorophenyl gives the target product in the highest yield (3e–3g). When R^1^ is *p*-bromophenyl and *p*-fluorophenyl, the target product was obtained in 75% and 73% yields, respectively (3h–3i). When R^1^ is a fused ring or a heterocyclic ring, such as 2-naphthyl and 2-furan groups, the corresponding products can also be successfully generated (3j–3k). When the R^1^ group was Ts-(3l) and Boc-(3m), the yields obtained were 51 and 96%, respectively. In addition, when R^1^ is aliphatic ethyl and lauryl, the target products can also be obtained in good to excellent yields (3n–3o).

To further expand the substrate scope, other simple olefins were tested next. As seen from Scheme 3, styrenes bearing either electron-donating or -withdrawing moieties can both be used equally well for this reaction, with slightly higher yields for the former (4a–4f). The yield of 2,3-dimethyl-2-butene was 42% (4g).

**Scheme 3:**
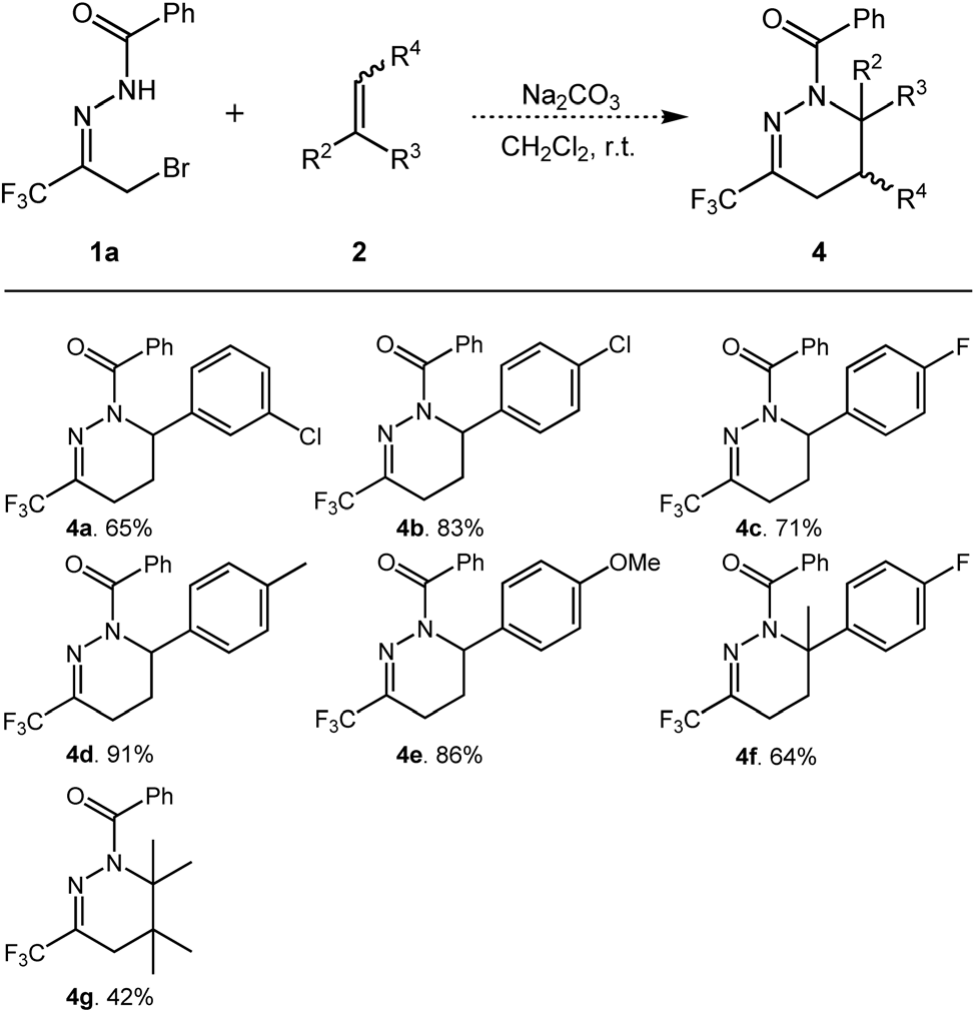
Substrate scope for simple alkenes^*a*,*b*^. ^*a*^All reactions were carried out by using 0.2 mmol of 1a, 3 eq. of 2 and 2 eq. of Na_2_CO_3_ in 3 mL of CH_2_Cl_2_. ^*b*^Isolated yields.

With α-bromo trifluoromethyl *N*-Boc acylhydrazone compounds, we also examined different olefins, and the results are shown in Scheme 4. Olefins bearing either electron-donating (5a–5b) or electron-withdrawing groups (5c–5d) could be applied to give good yields.

**Scheme 4:**
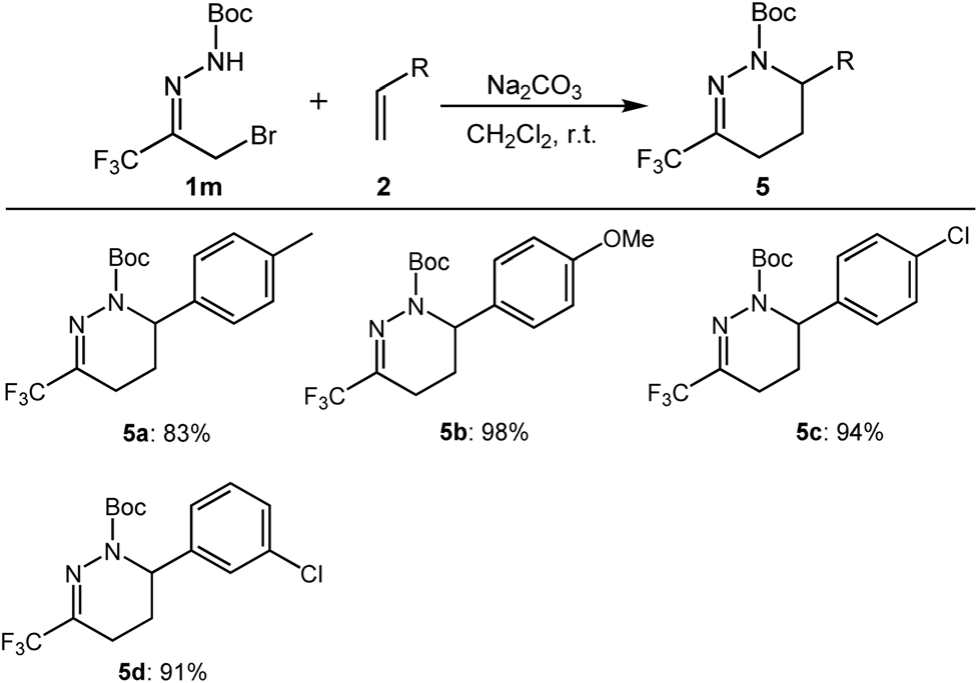
Substrate scope for hydrazone 1m with substituted styrenes^*a*,*b*^. ^*a*^All reactions were carried out by using 0.2 mmol of 1m, 3 eq. of 2 and 2 eq. of Na_2_CO_3_ in 3 mL of CH_2_Cl_2_. ^*b*^Isolated yields.

A plausible reaction mechanism was proposed based on a review of literature and reaction outcomes, as illustrated in Scheme 5. In the presence of a base, α-bromotrifluoromethyl acylhydrazone 1 undergoes dehydrohalogenation to eliminate one equivalent of HBr, generating the 1,2-diazabuta-1,3-diene intermediate 1a. This intermediate then participates in a Diels–Alder reaction with substituted olefin 2, forming the cyclic transition state 3a. The transition state subsequently evolves into the final product molecule 3, wherein the cleavage of preexisting bonds and the formation of new bonds occur in a concerted manner during a single mechanistic step.

**Scheme 5:**
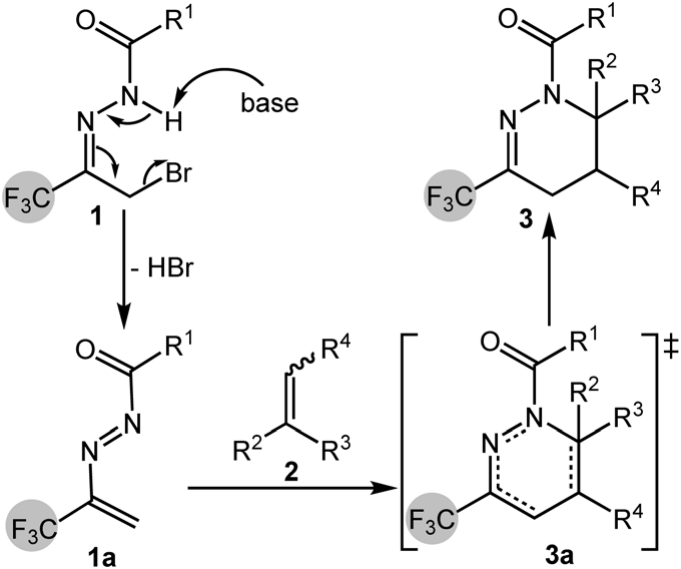
Proposed reaction mechanism.

## Conclusions

In summary, we reported here in a mild and catalyst-free [4 + 2] cycloaddition between *in situ* generated trifluoromethyl 1,2-diazabuta-1,3-diene with simple olefins. This protocol provides facile and atom economic access to trifluoromethyltetrahydropyridazine with moderate to excellent yields.

## Data availability

The authors confirm that the data supporting the findings of this study are available within the article and its ESI.†

## Author contributions

Yanhui Zhao: investigation, data curation, and methodology. Hemin Rong: investigation and data curation. Khurshed Bozorov: investigation and data curation. Buer Song: writing – original draft. Wei Liu: supervision and writing – review & editing. Xueqing Zhang: writing – review & editing, supervision, funding acquisition, and conceptualization.

## Conflicts of interest

There are no conflicts to declare.

## Supplementary Material

RA-015-D5RA03000E-s001
