# Supplementary material for: [4 + 2] Cycloaddition of α-bromotrifluoromethylhydrazone with alkenes: synthesis of trifluoromethyltetrahydropyridazines
Source: RSC Adv. 2025 Jun 9;15(24):19417–20. doi: 10.1039/d5ra03000e (PMC12146839; doi:10.1039/d5ra03000e)
Supplement: RA-015-D5RA03000E-s001 [file RA-015-D5RA03000E-s001.pdf]

Supplementary Information (SI) for RSC Advances.
This journal is © The Royal Society of Chemistry 2025

-Supporting Information

[4+2] cycloaddition of α-bromotrifluoromethylhydrazone with alkenes: synthesis of Trifluoromethyltetrahydropyridazines

Yanhui Zhao[1], Hemin Rong[1], Khurshed Bozorov[2], Xueqing Zhang[1,*], Buer Song[1,*] and Wei Liu[1,*]

1 Xinjiang Key Laboratory of Clean Conversion and High Value Utilization of Biomass Resources, School of Chemistry and Chemical Engineering, Yili Normal University, Xinjiang Yining 835000, China

2 Institute of Biochemistry, Samarkand State University, University Blvd. 15, Samarkand, 140104, Uzbekistan

*Corresponding author: Xueqing Zhang, Buer Song and Wei Liu

Email: zhxqsg@163.com; songbuer20211023@163.com.

Contents

1. General Information 2

2. General synthetic procedures 2-3

3. Characterization Data 3

3.1. NMR copies of compound **3a-3o**..3-8

3.2. NMR copies of compound **4a-4g**...8-11

3.3. NMR copies of compound **5a-5d**..11-12

4. References..12-13

5. NMR spectra...14-52

1. General Information

All solvents were treated according to standard procedures. Reagents were obtained from commercial suppliers except for acylhydrazines, and used without further purification unless otherwise noted. *α*-bromotrifluoromethylhydrazone **1** were prepared through condensation of hydrazides with 3-Bromo-1,1,1-trifluoroacetone according to the literature procedure.[1] Silica gel column chromatography was carried out using 230–400 mesh silica gel. The progress of reactions was monitored by TLC. TLC plates were analyzed by exposure to ultraviolet (UV) light or iodine vapor. NMR experiments were carried out in $CDCl_3$. ^1H and ^{13}CNMR spectra were recorded with 400 MHz or 600 MHz and 101 MHz or 151 MHz spectrometers, respectively. ^{19}F NMR spectra were recorded on 400 MHz and 600 MHz spectrometers, corresponding to Larmor frequencies of 376 MHz and 565 MHz for ^{19}F nuclei, respectively. Chemical shifts are reported as δ values relative to internal TMS (δ = 0.00 ppm for ^1H NMR), chloroform (δ = 7.26 ppm for ^1H NMR, δ = 77.0 ppm for ^{13}C NMR) in parts per million (ppm). The following abbreviations are used for the multiplicities: s: singlet, d: doublet, dd: doublet of doublet, t: triplet, q: quartet, m: multiplet, br: broad signal. Coupling constants (*J*) are reported in hertz (Hz). HRMS measurements were carried out using the ESI ionization technique with an FT-ICR analyzer.

2. General synthetic procedures

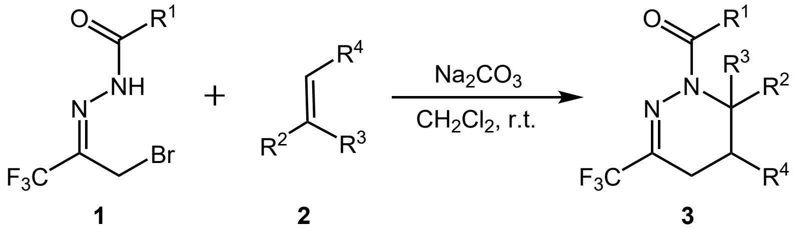

Trifluoromethylbromidoehydrazone **1** (0.3 mmol, 1.0 equiv), sodium carbonate (0.6 mmol, 2 equiv), olefins **2** (0.9 mmol, 3.0 equiv), and dichloromethane (2 mL) were added to a 50 mL round-bottom flask at room temperature. The degree of reaction was detected with TLC, and after the reaction was complete, the reaction mixture was quenched with a saturated aqueous solution NH_4Cl (10 mL). The organic layer was separated and the aqueous phase was extracted with EtOAc (3×15 mL). The pooled organic layer is dried on anhydrous $MgSO_4$ and then concentrated under reduced

pressure. The residue is purified by column chromatography on silica gel (petroleum ether-EtOAc) to obtain the desired product.

3. Characterization Data

3.1 NMR copies of compound 3a-3o

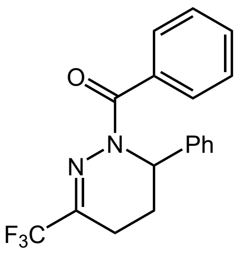

phenyl(6-phenyl-3-(trifluoromethyl)-5,6-dihydropyridazin-1(4*H*)-yl)methanone (3a): White crystals (55.17 mg, 83%); m.p: 104.2 – 106.4 °C; ^{1}H NMR (400 MHz, $CDCl_3$) δ 7.86 – 7.75 (m, 2H), 7.53 – 7.23 (m, 6H), 7.09 (d, J = 7.3 Hz, 2H), 6.06 (s, 1H), 2.47 – 2.28 (m, 2H), 2.18 (tt, J = 13.3, 4.8 Hz, 1H), 2.06 – 1.91 (m, 1H) ppm; ^{13}C NMR (101 MHz, $CDCl_3$) δ 169.9, 139.0, 137.8 (q, $J_{C\text{-}F}$ =35.4Hz), 133.3, 131.1, 130.4, 129.1, 127.7, 127.6, 125.1, 120.4 (q, $J_{C\text{-}F}$ = 274.7Hz), 52.3, 22.9, 16.4 ppm; ^{19}F NMR (376 MHz, $CDCl_3$) δ -71.18 ppm; HRMS (ESI): *m/z* [M + Na]$^+$ calcd for $C_{14}H_{13}F_3N_2O_3Na$: 337.0770; found: 337.0767.

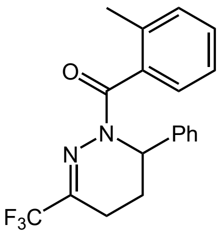

(6-phenyl-3-(trifluoromethyl)-5,6-dihydropyridazin-1(4*H*)-yl)(*o*-tolyl) methanone (3b): White crystals (50.23 mg, 78%); m.p: 106.4-109.6 °C; ^{1}H NMR (600 MHz, $CDCl_3$) δ 6.67 (t, J = 7.6 Hz, 2H), 6.64 – 6.55 (m, 3H), 6.54 – 6.48 (m, 2H), 6.40 (d, J = 7.6 Hz, 2H), 5.36 (s, 1H), 1.66 (dd, J = 18.3, 4.7 Hz, 1H), 1.60 (s, 4H), 1.43 (tt, J = 13.4, 4.8 Hz, 1H), 1.28 (ddd, J = 19.3, 12.8, 6.0 Hz, 1H); ^{13}C NMR (151 MHz, $CDCl_3$) δ 172.2, 139.5, 138.6 (q, $J_{C\text{-}F}$ =36.24 Hz), 135.6, 135.2, 130.3, 129.7, 129.4, 128.0, 127.8, 125.4, 125.4, 120.5 (q, $J_{C\text{-}F}$ =274.82 Hz), 52.3, 23.3, 20.0, 16.7; ^{19}F NMR (565 MHz, $CDCl_3$) δ -71.41; HRMS (ESI): *m/z* [M + H]$^+$ calcd for $C_{19}H_{18}F_3N_2O$: 347.1366; found: 347.1363.

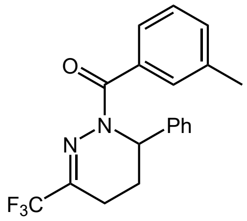

(6-phenyl-3-(trifluoromethyl)-5,6-dihydropyridazin-1(4*H*)-yl)(*m*-tolyl)methanone (3c): Colourless oil (59.25 mg, 92%) ; ^{1}H NMR (600 MHz, $CDCl_3$) δ 7.62 (s, 1H), 7.60 (dd, J = 5.5, 3.3 Hz, 1H), 7.34 (t, J = 7.7 Hz, 2H), 7.32 – 7.25 (m, 3H), 7.08 (d, J = 7.4 Hz, 2H), 6.06 – 6.01 (m, 1H), 2.39 (s, 4H), 2.34 – 2.29 (m, 1H), 2.15 (tt, J = 13.4, 4.8 Hz, 1H), 2.01 – 1.93 (m, 1H); ^{13}C NMR (151 MHz, $CDCl_3$) δ 170.0, 139.1, 137.6 (q, $J_{C\text{-}F}$ =36.24 Hz), 137.3, 133.2, 13

1.8, 131.0, 129.0, 127.6, 127.5, 127.4, 125.1, 120.4 (q, J_{C-F} =273.31 Hz), 52.3, 22.9, 21.3, 16.4; ^{19}F NMR (565 MHz, $CDCl_3$) δ -71.18; HRMS (ESI): *m/z* [M + H]$^+$ calcd for $C_{19}H_{18}F_3N_2O$: 347.1366; found: 347.1363.

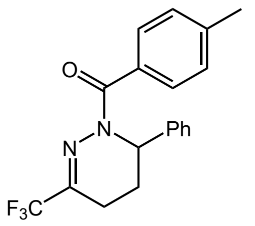

(6-phenyl-3-(trifluoromethyl)-5,6-dihydropyridazin-1(4*H*)-yl)(*p*-tolyl)methanone (3d): White solid (47.01 mg, 73%); m.p: 105.5-108.9 °C; ^{1}H NMR (600 MHz, $CDCl_3$) δ 7.75 (d, J = 8.3 Hz, 2H), 7.33 (t, J = 7.7 Hz, 2H), 7.26 (t, J = 7.4 Hz, 1H), 7.22 (d, J = 8.0 Hz, 2H), 7.07 (d, J = 7.5 Hz, 2H), 6.06 – 6.02 (m, 1H), 2.39 (s, 3H), 2.36 (dd, J = 17.9, 4.5 Hz, 1H), 2.34 – 2.28 (m, 1H), 2.15 (tt, J = 13.4, 4.8 Hz, 1H), 2.01 – 1.93 (m, 1H); ^{13}C NMR (151 MHz, $CDCl_3$) δ 169.7, 141.7, 139.2, 137.4 (q, J_{C-F} =36.24 Hz), 130.7, 130.3, 129.0, 128.3, 127.6, 125.1, 120.5 (q, J_{C-F} =274.83 Hz), 52.3, 22.9, 21.5, 16.4; ^{19}F NMR (565 MHz, $CDCl_3$) δ -71.09; HRMS (ESI): *m/z* [M + H]$^+$ calcd for $C_{19}H_{18}F_3N_2O$: 347.1366; found: 347.1363.

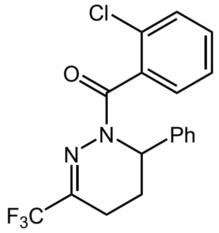

(2-chlorophenyl)(6-phenyl-3-(trifluoromethyl)-5,6-dihydropyridazin-1(4*H*)-yl)methanone (3e): White solid (48.32 mg, 66%); m.p: 119.6-121.3 °C; ^{1}H NMR (600 MHz, $CDCl_3$) δ 7.43 – 7.26 (m, 7H), 7.14 (d, J = 7.6 Hz, 2H), 6.02 (s, 1H), 2.36 (dd, J = 18.4, 4.8 Hz, 1H), 2.30 (dd, J = 13.6, 6.4 Hz, 1H), 2.14 (tt, J = 13. 4, 4.9 Hz, 1H), 1.97 (ddd, J = 19.3, 12.9, 6.2 Hz, 1H); ^{13}C NMR (151 MHz, $CDCl_3$) δ 169.2, 139.0 (q, J_{C-F} =36.24 Hz), 138.8, 135.2, 131.2, 130.5, 129.2, 129.0, 128.5, 127.4, 126.5, 125.2, 120.1 (q, J_{C-F} =274.82 Hz), 52.2, 22.8, 16.5; ^{19}F NMR (565 MHz, $CDCl_3$) δ -71.64; HRMS (ESI): *m/z* [M + H]$^+$ calcd for $C_{18}H_{15}F_3N_2OCl$: 367.0820; found: 367.0816.

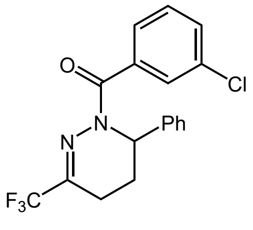

(3-chlorophenyl)(6-phenyl-3-(trifluoromethyl)-5,6-dihydropyridazin-1(4*H*)-yl)methanone (3f): White solid (61.50 mg, 84%); m.p: 115.5-119.4 °C; ^{1}H NMR (600 MHz, $CDCl_3$) δ 7.80 (t, J = 1.9 Hz, 1H), 7.68 (dt, J = 7.8, 1.4 Hz, 1H), 7.45 (ddd, J = 8.0, 2.3, 1.0 Hz, 1H), 7.39 – 7.32 (m, 3H), 7.31 – 7.26 (m, 1 H), 7.07 (d, J = 7.5 Hz, 2H), 6.05 – 5.97 (m, 1H), 2.45 – 2.27 (m, 2H), 2.16 (tt, J = 13.4, 4.9 Hz, 1H), 2.05 – 1.93 (m, 1H); ^{13}C NMR (151 MHz, $CDCl_3$)

δ 168.44, 138.8, 138.5 (q, J_{C-F} =34.73 Hz), 135.0, 133.7, 131.1, 130.4, 129.2, 128.9, 128.5, 127.8, 125.0, 120.3 (q, J_{C-F} =273.31 Hz), 52.4, 22.9, 16.4; ^{19}F NMR (565 MHz, $CDCl_3$) δ -71.32; HRMS (ESI): *m/z* [M + H] $^+$ calcd for $C_{18}H_{15}F_3N_2OCl$: 367.0820; found: 367.0816.

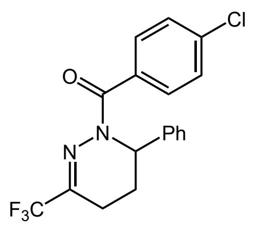

(4-chlorophenyl)(6-phenyl-3-(trifluoromethyl)-5,6-dihydropyridazin-1(4*H*)-yl)methanone (3g): White solid (46.12 mg, 63%); m.p: 98.6-101.5 °C; ^{1}H NMR (600 MHz, $CDCl_3$) δ 7.77 (d, J = 8.6 Hz, 2H), 7.42 – 7.37 (m, 2H), 7.35 (t, J = 7.6 Hz, 2H), 7.28 (t, J = 7.4 Hz, 1H), 7.06 (d, J = 7.6 Hz, 2H), 6.02 (dt, J = 4.3, 2.0 Hz, 1H), 2.39 (dd, J = 18.4, 4.9 Hz, 1H), 2.33 (dd, J = 13.6, 6.4 Hz, 1H), 2.16 (tt, J = 13.4, 4.8 Hz, 1H), 2.04 – 1.95 (m, 1H).^{13}C NMR (151 MHz, $CDCl_3$) δ 168.7, 138.9, 138.3 (q, J_{C-F} =36.24 Hz) , 137.4, 131.9, 131.2, 129.1, 127.9, 127.8, 125.0, 120.3 (q, J_{C-F} =273.31 Hz), 52.4, 22.9, 16.4; ^{19}F NMR (565 MHz, $CDCl_3$) δ -71.21; HRMS (ESI): *m/z* [M + H]$^+$ calcd for $C_{18}H_{15}F_3N_2OCl$: 367.0820; found: 367.0816.

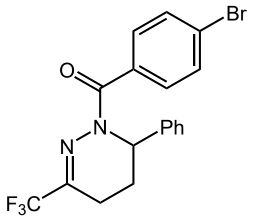

(4-bromophenyl)(6-phenyl-3-(trifluoromethyl)-5,6-dihydropyridazin-1(4*H*)-yl)methanone (3h): White solid (61.50 mg, 75%); m.p: 117.1-121.3 °C; ^{1}H NMR (600 MHz, $CDCl_3$) δ 7.69 (d, J = 8.6 Hz, 2H), 7.55 (d, J = 8.5 Hz, 2H), 7.38 – 7.32 (m, 2H), 7.28 (t, J = 7.4 Hz, 1H), 7.06 (d, J = 7.5 Hz, 2H), 6.04 – 5.99 (m, 1H), 2.38 (dd, J = 18.4, 4.9 Hz, 1H), 2.32 (dd, J = 13.6, 6.5 Hz, 1H), 2.16 (tt, J = 13.4, 4.8 Hz, 1H), 1.99 (ddd, J = 19.8, 13.0, 6.3 Hz, 1H);
^{13}C NMR (151 MHz, $CDCl_3$) δ 168.8, 138.9, 138.3 (q, J_{C-F} =34.73 Hz), 132.1, 132.1, 130.9, 129.1, 127.8, 125.9, 125.0, 120.3 (q, J_{C-F} =274.82 Hz), 52.4, 22.9, 16.4; ^{19}F NMR (565 MHz, $CDCl_3$) δ -71.19; HRMS (ESI): *m/z* [M + H]$^+$ calcd for $C_{18}H_{15}F_3N_2OBr$: 411.0314; found: 411.0310.

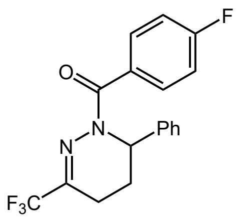

(4-fluorophenyl)(6-phenyl-3-(trifluoromethyl)-5,6-dihydropyridazin-1(4*H*)-yl)methanon (3i): White solid (51.11 mg, 73%); m.p: 75.6-77.5 °C; ^{1}H NMR (600 MHz, $CDCl_3$) δ 7.89 – 7.84 (m, 2H), 7.35 (t, J = 7.6 Hz, 2H), 7.28 (t, J = 7.4 Hz,

1H), 7.13 – 7.05 (m, 4H), 6.05 – 6.01 (m, 1H), 2.39 (dd, J = 18.4, 4.9 Hz, 1H), 2.34 (ddt, J = 13.6, 6.4, 2.1 Hz, 1H), 2.17 (tt, J = 13.4, 4.8 Hz, 1H), 2.04 – 1.95 (m, 1H); ^{13}C NMR (151 MHz, $CDCl_3$) δ 168.6, 164.4(d, $J_{C\text{-}F}$ = 253.68 Hz), 139.0, 138.0 (q, $J_{C\text{-}F}$ =36.24 Hz), 133.1 (d, $J_{C\text{-}F}$ = 9.06 Hz), 129.3, 129.3 (d, $J_{C\text{-}F}$ = 3.02 Hz), 129.1, 125.0, 120.4(q, $J_{C\text{-}F}$ = 274.82 Hz), 114.7 (d, $J_{C\text{-}F}$ = 22.65 Hz), 52.4, 22.9, 16.4; ^{19}F NMR (565 MHz, $CDCl_3$) δ -71.22, -108.09 – -108.17 (m); HRMS (ESI): m/z $[M + H]^+$ calcd for $C_{18}H_{15}F_4N_2O$: 351.1115; found: 351.1118.

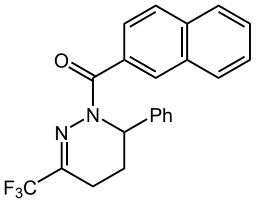

naphthalen-2-yl(6-phenyl-3-(trifluoromethyl)-5,6-dihydropyridazin-1(4*H*)-yl)methanone (3j): White solid (59.61 mg, 78%); m.p: 132.0-134.9 °C; ^{1}H NMR (600 MHz, $CDCl_3$) δ 8.40 (s, 1H), 7.91 (d, J = 8.1 Hz, 1H), 7.85 (dd, J = 8.6, 5.1 Hz, 3H), 7.57 – 7.49 (m, 2H), 7.36 (t, J = 7.7 Hz, 2H), 7.28 (t, J = 7.5 Hz, 1H), 7.12 (d, J = 7.3 Hz, 2H), 6.12 – 6.05 (m, 1H), 2.39 (dd, J = 18.3, 4.9 Hz, 1H), 2.36 – 2.31 (m, 1H), 2.19 (tt, J = 13.4, 4.9 Hz, 1H), 2.04 – 1.96 (m, 1H); ^{13}C NMR (151 MHz, $CDCl_3$) δ 169.8, 139.1, 137.9 (q, $J_{C\text{-}F}$ = 34.73 Hz), 134.5, 132.3, 131.6, 130.7, 129.2, 129.1, 127.8, 127.7, 127.7, 126.9, 126.8, 126.4, 125.1, 120.4 (q, $J_{C\text{-}F}$ = 274.82 Hz), 52.4, 23.0, 16.4; ^{19}F NMR (565 MHz, $CDCl_3$) δ -71.09; HRMS (ESI): m/z $[M + H]^+$ calcd for $C_{22}H_{18}F_3N_2O$: 383.1366; found: 383.1362.

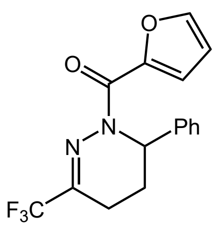

furan-2-yl(6-phenyl-3-(trifluoromethyl)-5,6-dihydropyridazin-1(4*H*)-yl)methanone (3k): Colourless oil (56.04 mg, 87%); ^{1}H NMR (600 MHz, $CDCl_3$) δ 7.64 (dd, J = 6.0, 2.6 Hz, 2H), 7.32 (t, J = 7.6 Hz, 2H), 7.29 – 7.23 (m, 1H), 7.04 (d, J = 7.5 Hz, 2H), 6.56 (dd, J = 3.5, 1.7 Hz, 1H), 6.06 – 5.99 (m, 1H), 2.45 – 2.37 (m, 1H), 2.36 – 2.29 (m, 1H), 2.13 (tt, J = 13.3, 4.7 Hz, 1H), 2.06 – 1.97 (m, 1H); ^{13}C NMR (151 MHz, $CDCl_3$) δ 158.4, 146.3, 145.2, 138.8, 138.2 (q, $J_{C\text{-}F}$ = 36.24 Hz), 129.0, 127.7, 125.1, 121.6, 120.7 (q, $J_{C\text{-}F}$ = 274.82 Hz), 112.1, 52.0, 22.7, 16.5; ^{19}F NMR (565 MHz, $CDCl_3$) δ -70.91; HRMS (ESI): m/z $[M + H]^+$ calcd for $C_{16}H_{14}F_3N_2O_2$: 323.1002; found: 323.1000.

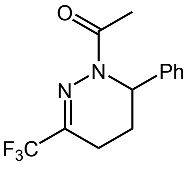

1-(6-phenyl-3-(trifluoromethyl)-5,6-dihydropyridazin-1(4*H*)-

yl)ethan-1-one (3l): Colourless oil (43.76 mg, 81%); ^{1}H NMR (600 MHz, $CDCl_3$) δ 7.32 (t, J = 7.6 Hz, 2H), 7.25 (t, J = 7.4 Hz, 1H), 6.97 (dd, J = 7.9, 1.4 Hz, 2H), 5.91 – 5.86 (m, 1H), 2.43 (s, 3H), 2.37 – 2.30 (m, 1H), 2.27 – 2.21 (m, 1H), 2.01 (tt, J = 13.1, 4.6 Hz, 1H), 1.97 – 1.88 (m, 1H); ^{13}C NMR (151 MHz, $CDCl_3$) δ 172.4, 139.2, 137.4 (q, $J_{C\text{-}F}$ = 33.22 Hz), 129.0, 127.6, 125.0, 120.4 (q, $J_{C\text{-}F}$ = 273.31 Hz), 51.3, 22.7, 21.1, 16.2; ^{19}F NMR (565 MHz, $CDCl_3$) δ -71.35; HRMS (ESI): *m/z* $[M + H]^+$ calcd for $C_{13}H_{14}F_3N_2O$: 271.1053; found: 271.1052.

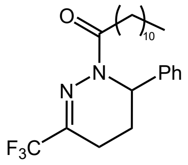

1-(6-phenyl-3-(trifluoromethyl)-5,6-dihydropyridazin-1(4*H*)-yl)dodecan-1-one (3m): Colourless oil (74.67 mg, 91%); ^{1}H NMR (600 MHz, $CDCl_3$) δ 7.31 (t, J = 7.6 Hz, 2H), 7.27 – 7.22 (m, 1H), 6.96 (d, J = 7.1 Hz, 2H), 5.91 – 5.84 (m, 1H), 2.86 (ddd, J = 15.3, 8.7, 6.5 Hz, 1H), 2.76 (ddd, J = 15.3, 8.6, 6.6 Hz, 1H), 2.37 – 2.30 (m, 1H), 2.23 (ddd, J = 8.2, 6.2, 4.1 Hz, 1H), 2.05 – 1.88 (m, 2H), 1.73 – 1.60 (m, 2H), 1.26 (s, 16H), 0.88 (t, J = 7.0 Hz, 3H); ^{13}C NMR (151 MHz, $CDCl_3$) δ 174.9, 139.4, 136.9 (q, $J_{C\text{-}F}$ = 36.24 Hz), 128.9, 127.5, 125.0, 120.5 (q, $J_{C\text{-}F}$ = 273.31 Hz), 51.3, 33.1, 31.9, 29.6, 29.5, 29.4, 24.9, 22.8, 22.7, 16.2, 14.1; ^{19}F NMR (565 MHz, $CDCl_3$) δ -71.25; HRMS (ESI): *m/z* $[M + H]^+$ calcd for $C_{23}H_{34}F_3N_2O$: 411.2618; found: 411.2615.

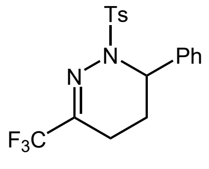

6-phenyl-1-tosyl-3-(trifluoromethyl)-1,4,5,6-tetrahydropyridazine (3n): White solid (38.97 mg, 51%); m.p: 142.7-144.9 ^{o}C; ^{1}H NMR (600 MHz, $CDCl_3$) δ 7.65 – 7.59 (m, 2H), 7.25 – 7.18 (m, 5H), 6.90 – 6.82 (m, 2H), 5.64 (s, 1H), 2.41 (s, 3H), 2.31 – 2.25 (m, 1H), 2.12 (ddt, J = 10.9, 4.0, 2.3 Hz, 1H), 1.98 – 1.85 (m, 2H), ^{13}C NMR (151 MHz, $CDCl_3$) δ 144.4, 139.4, 137.7 (q, $J_{C\text{-}F}$ = 34.73 Hz), 135.0, 129.4, 128.7, 128.1, 127.7, 125.7, 120.2 (q, $J_{C\text{-}F}$ = 273.31 Hz), 55.8, 23.7, 21.6, 15.4; ^{19}F NMR (565 MHz, $CDCl_3$) δ -71.20; HRMS (ESI): *m/z* $[M + H]^+$ calcd for $C_{18}H_{18}F_3N_2O_2S$: 383.1036; found: 383.1034.

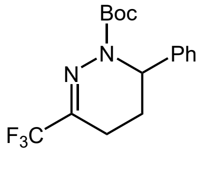

tert-butyl-6-phenyl-3-(trifluoromethyl)-5,6-dihydropyridazine-1(4*H*)-carboxylate (3o): White solid (63.00 mg, 96%); m.p: 108.6-111.3 ^{o}C; ^{1}H NMR (600 MHz, $CDCl_3$) δ 7.33 (t, J = 7.6 Hz, 2H), 7.29 – 7.24 (m, 1H), 7.02 (d, J = 7.5 Hz, 2H), 5.56 (s, 1H), 2.30 (dd, J = 18.2, 4.9 Hz, 1H), 2.23 – 2.17 (m, 1H), 2.10 (tt, J = 13.3, 4.9 Hz, 1H), 1.94 – 1.85 (m, 1H), 1.43 (s,

9H); ^{13}C NMR (151 MHz, $CDCl_3$) δ 152.0, 140.2, 137.1 (q, $J_{C\text{-}F}$ = 36.24 Hz), 128.9, 127.5, 124.9, 120.3 (q, $J_{C\text{-}F}$ = 274.82 Hz), 82.7, 54.3, 28.0, 23.1, 15.7; ^{19}F NMR (565 MHz, $CDCl_3$) δ -71.23; HRMS (ESI): *m/z* $[M + H]^+$ calcd for $C_{16}H_{20}F_3N_2O_2$: 329.1471; found: 329.1474.

3.2. NMR copies of compound 4a-4g

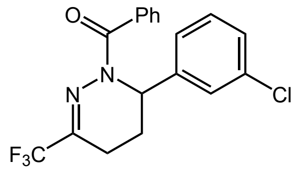

(6-(3-chlorophenyl)-3-(trifluoromethyl)-5,6-dihydropyridazin-1(4*H*)-yl)(phenyl)methanone (4a): White solid (47.59 mg, 65%); m.p: 108.6-111.3 °C; ^{1}H NMR (600 MHz, $CDCl_3$) δ 7.99 – 7.90 (m, 2H), 7.68 – 7.62 (m, 1H), 7.58 (t, J = 7.7 Hz, 2H), 7.47 – 7.37 (m, 2H), 7.26 (s, 1H), 7.09 (dt, J = 6.8, 2.1 Hz, 1H), 6.19 – 6.11 (m, 1H), 2.56 (dd, J = 18.5, 5.0 Hz, 1H), 2.48 (dd, J = 13.7, 6.5 Hz, 1H), 2.34 (tt, J = 13.5, 4.9 Hz, 1H), 2.18 – 2.09 (m, 1H); ^{13}C NMR (151 MHz, $CDCl_3$) δ 169.8, 141.2, 137.9 (q, $J_{C\text{-}F}$ = 34.73 Hz), 135.1, 133.0, 131.3, 130.4, 130.4, 128.0, 127.7, 125.6, 123.2, 120.3 (q, $J_{C\text{-}F}$ = 274.82 Hz), 51.9, 22.8, 16.3; ^{19}F NMR (565 MHz, $CDCl_3$) δ -71.20; HRMS (ESI): *m/z* $[M + H]^+$ calcd for $C_{18}H_{15}F_3N_2OCl$: 367.0820; found: 367.0816.

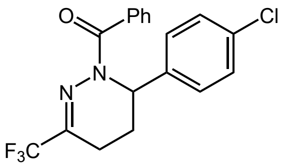

(6-(4-chlorophenyl)-3-(trifluoromethyl)-5,6-dihydropyridazin-1(4*H*)-yl)(phenyl)methanone (4b): White solid (60.77 mg, 83%); m.p: 88.5-91.3 °C; ^{1}H NMR (600 MHz, $CDCl_3$) δ 7.83 – 7.75 (m, 2H), 7.52 – 7.46 (m, 1H), 7.42 (t, J = 7.6 Hz, 2H), 7.37 – 7.29 (m, 2H), 7.07 – 6.99 (m, 2H), 6.04 – 5.97 (m, 1H), 2.40 (dd, J = 18.5, 5.0 Hz, 1H), 2.33 – 2.27 (m, 1H), 2.18 (tt, J = 13.5, 4.9 Hz, 1H), 2.01 – 1.91 (m, 1H); ^{13}C NMR (151 MHz, $CDCl_3$) δ 169.8, 137.8 (q, $J_{C\text{-}F}$ = 36.24 Hz), 137.6, 133.6, 133.0, 131.3, 130.4, 129.3, 127.6, 126.6, 120.3 (q, $J_{C\text{-}F}$ = 274.82 Hz), 51.8, 22.8, 16.3; ^{19}F NMR (565 MHz, $CDCl_3$) δ -71.20; HRMS (ESI): *m/z* $[M + H]^+$ calcd for $C_{18}H_{15}F_3N_2OCl$: 367.0820; found: 367.0816.

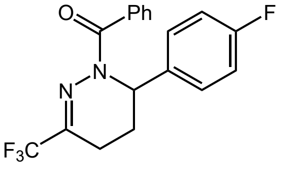

(6-(4-fluorophenyl)-3-(trifluoromethyl)-5,6-dihydropyridazin-1(4*H*)-yl)(phenyl)methanone (4c): Yellow oily liquid (49.71 mg, 71%); ^{1}H NMR (600 MHz, $CDCl_3$) δ 7.82

– 7.76 (m, 2H), 7.51 – 7.46 (m, 1H), 7.42 (t, *J* = 7.6 Hz, 2H), 7.10 – 7.01 (m, 4H), 6.04 – 5.99 (m, 1H), 2.40 (dd, *J* = 18.5, 4.9 Hz, 1H), 2.30 (ddt, *J* = 13.7, 6.5, 2.1 Hz, 1H), 2.17 (tt, *J* = 13.4, 4.8 Hz, 1H), 2.02 – 1.93 (m, 1H); ^{13}C NMR (151 MHz, $CDCl_3$) δ 169.8, 162.2 (d, $J_{C\text{-}F}$ = 247.64 Hz), 137.8 (q, $J_{C\text{-}F}$ = 36.24 Hz), 134.1 (d, $J_{C\text{-}F}$ = 3.02 Hz), 133.1, 131.3, 130.4, 127.6, 126.8 (d, $J_{C\text{-}F}$ = 9.06 Hz), 120.3 (q, $J_{C\text{-}F}$ = 274.82 Hz), 116.1 (d, $J_{C\text{-}F}$ = 19.63 Hz), 51.7, 22.9, 16.3; ^{19}F NMR (565 MHz, $CDCl_3$) δ -71.19, -114.70 – -114.76 (m); HRMS (ESI): *m/z* $[M + H]^+$ calcd for $C_{18}H_{14}F_4N_2O$: 350.1042; found: 350.103 8.

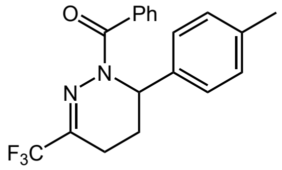

phenyl(6-(*p*-tolyl)-3-(trifluoromethyl)-5,6-dihydropyridaz in-1(4*H*)-yl)methanone (4d): Yellow oily liquid (63.00 m g, 91%); ^{1}H NMR (600 MHz, $CDCl_3$) δ 7.82 – 7.77 (m, 2H), 7.49 – 7.44 (m, 1H), 7.41 (ddt, *J* = 8.3, 6.6, 1.4 Hz, 2H), 7.15 (d, *J* = 8.0 Hz, 2H), 6.97 (d, *J* = 8.1 Hz, 2H), 6.02 – 5.98 (m, 1H), 2.39 – 2.33 (m, 1H), 2.32 (s, 3H), 2.30 – 2.27 (m, 1H), 2.13 (tt, *J* = 13.3, 4.8 Hz, 1H), 2.04 – 1.95 (m, 1H); ^{13}C NMR (151 MHz, $CDCl_3$) δ 169.9, 137.8 (q, $J_{C\text{-}F}$ = 34.73 Hz), 137.4, 136.1, 133.4, 131.1, 130.4, 129.8, 127.6, 125.0, 120.4 (q, $J_{C\text{-}F}$ = 2 74.82 Hz), 52.1, 22.9, 21.0, 16.4; ^{19}F NMR (565 MHz, $CDCl_3$) δ -71.16; HR MS (ESI): *m/z* $[M + H]^+$ calcd for $C_{19}H_{17}F_3N_2O$: 346.1293; found: 346.1289.

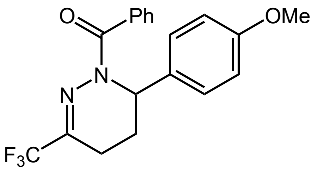

(6-(4-methoxyphenyl)-3-(trifluoromethyl)-5,6-dihydrop yridazin-1(4*H*)-yl)(phenyl)methanone (4e) Colourless oi l (62.28 mg, 86%); ^{1}H NMR (600 MHz, $CDCl_3$) δ 7. 82 – 7.76 (m, 2H), 7.50 – 7.45 (m, 1H), 7.41 (t, *J* = 7.6 Hz, 2H), 7.00 (d, *J* = 8.6 Hz, 2H), 6.90 – 6.85 (m, 2H), 5.99 (s, 1H), 3.78 (s, 3H), 2.38 (dd, *J* = 18.3, 4.9 Hz, 1H), 2.29 (dd, *J* = 13.4, 6.5 Hz, 1H), 2.13 (tt, *J* = 13.3, 4.7 Hz, 1H), 2.06 – 1.97 (m, 1H); ^{13}C NMR (151 MHz, $CDCl_3$) δ 169.8, 159.0, 137.8 (q, $J_{C\text{-}F}$ =34.73 Hz), 133.4, 131.1, 131.0, 130.3, 127.6, 126.2, 120.4 (q, $J_{C\text{-}F}$ = 273.31 Hz), 114.5, 55.3, 51.8, 23.0, 16.4; ^{19}F NMR (565 MHz, $CDCl_3$) δ -71. 18; HRMS (ESI): *m/z* $[M + H]^+$ calcd for $C_{19}H_{18}F_3N_2O_2$: 363.1315; found: 363.1 313.

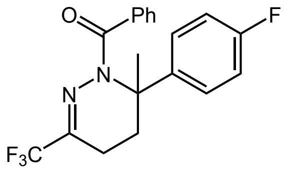

(6-(4-fluorophenyl)-6-methyl-3-(trifluoromethyl)-5,6-dihydropyridazin-1(4*H*)-yl)(phenyl)methanone (4f): Colourless oil (46.61 mg, 64%); ^{1}H NMR (600 MHz, C DCl$_3$) δ 7.73 – 7.68 (m, 2H), 7.48 – 7.44 (m, 1H), 7.38 (t, J = 7.6 Hz, 2H), 7.21 – 7.15 (m, 2H), 7.07 – 6.99 (m, 2H), 2.43 – 2.35 (m, 1H), 2.20 – 2.14 (m, 1H), 2.14 – 2.07 (m, 2H), 2.01 (s, 3H); ^{13}C NMR (151 MHz, CDCl$_3$) δ 1 70.7, 162.5, 160.9, 139.5, 139.5, 135.9 (q, J_{C-F} = 36.24 Hz), 134.5, 131.2, 130.2 , 127.3, 125.9, 125.9, 120.4 (q, J_{C-F} = 274.82 Hz), 115.8, 115.6, 60.1, 35.0, 2 4.8, 17.9; ^{19}F NMR (565 MHz, CDCl$_3$) δ -71.09, -115.86 (ddd, J = 13.7, 8.5, 5.0 Hz); HRMS (ESI): *m/z* [M + H]$^+$ calcd for $C_{19}H_{17}F_4N_2O$: 365.1272; found: 365.1268.

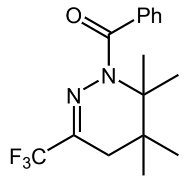

phenyl(5,5,6,6-tetramethyl-3-(trifluoromethyl)-5,6-dihydropyrida zin-1(4*H*)-yl)methanone (4g): Yellow oily liquid (26.22 mg, 42%); ^{1}H NMR (600 MHz, CDCl$_3$) δ 7.60 – 7.56 (m, 2H), 7.45 – 7.4 1 (m, 1H), 7.36 (t, J = 7.7 Hz, 2H), 2.22 (s, 2H), 1.58 (s, 6H), 1.09 (s, 6H); ^{13}C NMR (151 MHz, CDCl$_3$) δ 172.7, 136.1, 134.1 (q, J_{C-F} = 36.24 Hz), 130. 9, 129.8, 127.5, 120.5 (q, J_{C-F} = 273.31 Hz), 62.4, 33.5, 33.1, 24.0, 20.4; ^{19}F NMR (565 MHz, CDCl$_3$) δ -71.26. HRMS (ESI): *m/z* [M + H]$^+$ calcd for $C_{16}H_{19}F_3N_2O$: 312.1450; found: 312.1448.

3. NMR copies of compound 5a-5d

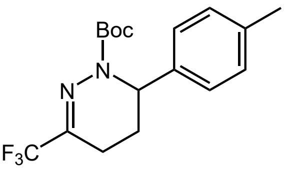

tert-butyl-6-(*p*-tolyl)-3-(trifluoromethyl)-5,6-dihydropyri dazine-1(4*H*)-carboxylate (5a): White solid (56.80 mg, 83%); m.p: 65.4.5-68.3 °C; ^{1}H NMR (600 MHz, CDCl$_3$) δ 7.13 (d, J = 7.8 Hz, 2H), 6.91 (d, J = 8.0 Hz, 2H), 5.53 (s, 1H), 2.32 (s, 3H), 2.29 (dd, J = 18.1, 4.3 Hz, 1H), 2.17 (dd, J = 13.4, 6.5 Hz, 1H), 2.07 (tt, J = 13.2, 4.8 Hz, 1H), 1.94 – 1.85 (m, 1H), 1.43 (s, 9H); ^{13}C NMR (101 MHz, CDCl$_3$) δ 152.1, 137.1, 137.1 (q, J_{C-F} = 36.36 Hz), 129.6, 124.8, 120.6 (q, J_{C-F} = 274.72 Hz), 82.6, 54.1, 28.0, 23.1, 21.0, 15.7; ^{19}F NMR (565 MHz, CDCl$_3$) δ -71.22; HRMS (ESI): *m/z* [M + H]$^+$ calcd for $C_{17}H_{21}F_3N_2O_2$: 342.15 55; found: 342.1551.

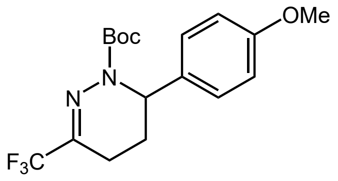

tert-butyl-6-(4-methoxyphenyl)-3-(trifluoromethyl)-5,6-dihydropyridazine-1(4*H*)-carboxylate (5b): White solid (70.20 mg, 98%); m.p: 84.2-86.3 °C; ^{1}H NMR (600 MHz, $CDCl_3$) δ 6.96 – 6.92 (m, 2H), 6.88 – 6.84 (m, 2H), 5.51 (s, 1H), 3.79 (s, 3H), 2.30 (dd, J = 18.2, 4.8 Hz, 1H), 2.19 – 2.12 (m, 1H), 2.06 (tt, J = 13.3, 4.7 Hz, 1H), 1.96 – 1.87 (m, 1H), 1.44 (s, 9H); ^{13}C NMR (151 MHz, $CDCl_3$) δ 158.9, 152.1, 137.1 (q, J_{C-F} = 34.73 Hz), 132.2, 126.1, 120.6 (q, J_{C-F} = 273.31 Hz), 114.2, 82.6, 55.3, 53.8, 28.0, 23.2, 15.7; ^{19}F NMR (565 MHz, $CDCl_3$) δ -71.20; HRMS (ESI): *m/z* $[M + H]^+$ calcd for $C_{17}H_{21}F_3N_2O_3$: 358.1504; found: 358.1508.

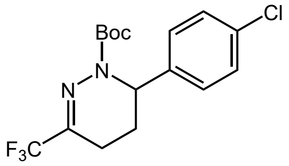

tert-butyl-6-(4-chlorophenyl)-3-(trifluoromethyl)-5,6-dihydropyridazine-1(4*H*)-carboxylate (5c): White solid (68.07 mg, 94%); m.p:116.5-118.9 °C; ^{1}H NMR (600 MHz, $CDCl_3$) δ 7.33 – 7.30 (m, 2H), 6.97 (d, J = 8.5 Hz, 2H), 5.54 (s, 1H), 2.33 (dd, J = 18.3, 4.6 Hz, 1H), 2.17 (ddt, J = 13.6, 6.7, 2.1 Hz, 1H), 2.10 (tt, J = 13.4, 4.9 Hz, 1H), 1.91 – 1.82 (m, 1H), 1.44 (s, 9H); ^{13}C NMR (151 MHz, $CDCl_3$) δ 151.9, 138.7, 137.1 (q, J_{C-F} = 36.24 Hz), 133.4, 129.1, 126.4, 120.5 (q, J_{C-F} = 273.31 Hz), 83.0, 53.7, 28.0, 23.0, 15.6; ^{19}F NMR (565 MHz, $CDCl_3$) δ -71.24; HRMS (ESI): *m/z* $[M + H]^+$ calcd for $C_{16}H_{18}F_3N_2O_2Cl$: 362.1009; found: 362.1011.

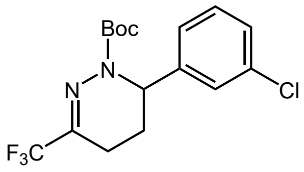

tert-butyl-6-(3-chlorophenyl)-3-(trifluoromethyl)-5,6-dihydropyridazine-1(4*H*)-carboxylate(5d): White solid (65.90 mg, 91%); m.p:114.2.5-117.3 °C; ^{1}H NMR (600 MHz, $CDCl_3$) δ 7.30 – 7.24 (m, 2H), 7.06 (s, 1H), 6.89 (dt, J = 6.8, 1.9 Hz, 1H), 5.53 (s, 1H), 2.33 (dd, J = 18.2, 4.9 Hz, 1H), 2.20 (ddt, J = 13.6, 6.5, 2.1 Hz, 1H), 2.11 (tt, J = 13.4, 4.9 Hz, 1H), 1.93 – 1.84 (m, 1H), 1.45 (s, 9H); ^{13}C NMR (151 MHz, $CDCl_3$) δ 151.9, 142.3, 137.2 (q, J_{C-F} = 34.73 Hz), 135.0, 127.9, 125.3, 123.1, 120.5 (q, J_{C-F} = 273.31 Hz), 83.1, 53.8, 28.0, 22.9, 15.6; ^{19}F NMR (565 MHz, $CDCl_3$) δ -71.24; HRMS (ESI): *m/z* $[M + H]^+$ calcd for $C_{16}H_{18}F_3N_2O_2Cl$: 362.1009; found: 362.1004.

4. References

1. Hatcher, J. M., Coltart, D. M., Copper(I)-Catalyzed Addition of Grignard Reagents to *in Situ*-Derived *N*-Sulfonyl Azoalkenes: An Umpolung Alkylation

Procedure Applicable to the Formation of Up to Three Contiguous Quaternary Centers. J. Am. Chem. Soc. 2010, 132, 4546–4547; (b) Chen, J.-R., Dong, W.-R., Candy, M., Pan, F.-F., Jörres, M., Bolm, C., Enantioselective Synthesis of Dihydropyrazoles by Formal [4 + 1] Cycloaddition of *in Situ*-Derived Azoalkenes and Sulfur Ylides. J. Am. Chem. Soc., 2012, 134, 6924–6927; (c) Chen, D.-Z., Xiao, W.-J., Chen, J.-R., Synthesis of Spiropyrazoline Oxindoles by a Formal [4 + 1] Annulation Reaction Between 3-Bromooxindoles and *in Situ*-Derived 1,2-Diaza-1,3-Dienes. Org. Chem. Front., 2017, 4, 1289–1293; (d) Li, D., Metal- and Azide-Free Iodine-Promoted Aerobic Oxidative Cyclization to Trifluoromethylated Triazoles. J. Org. Chem. 2025, 90, 1256–1261.

5. NMR spectra

NMR copies of compound **3a:**

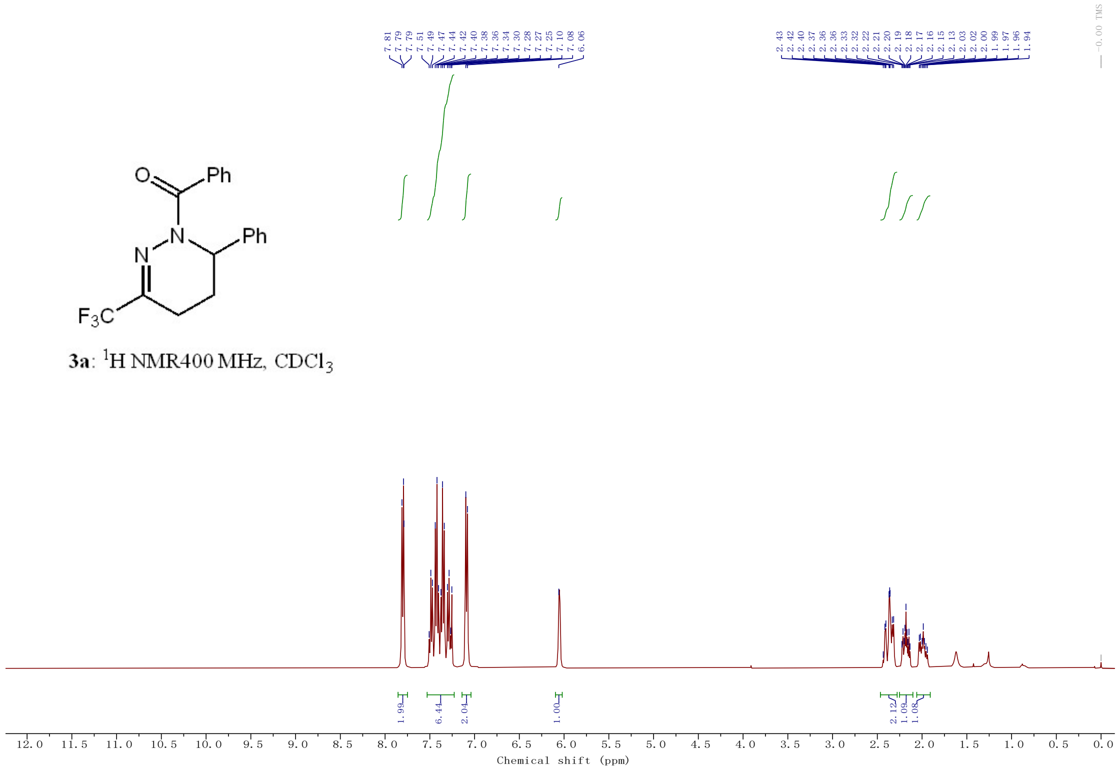

3a: ^{1}H NMR400 MHz, CDCl$_3$

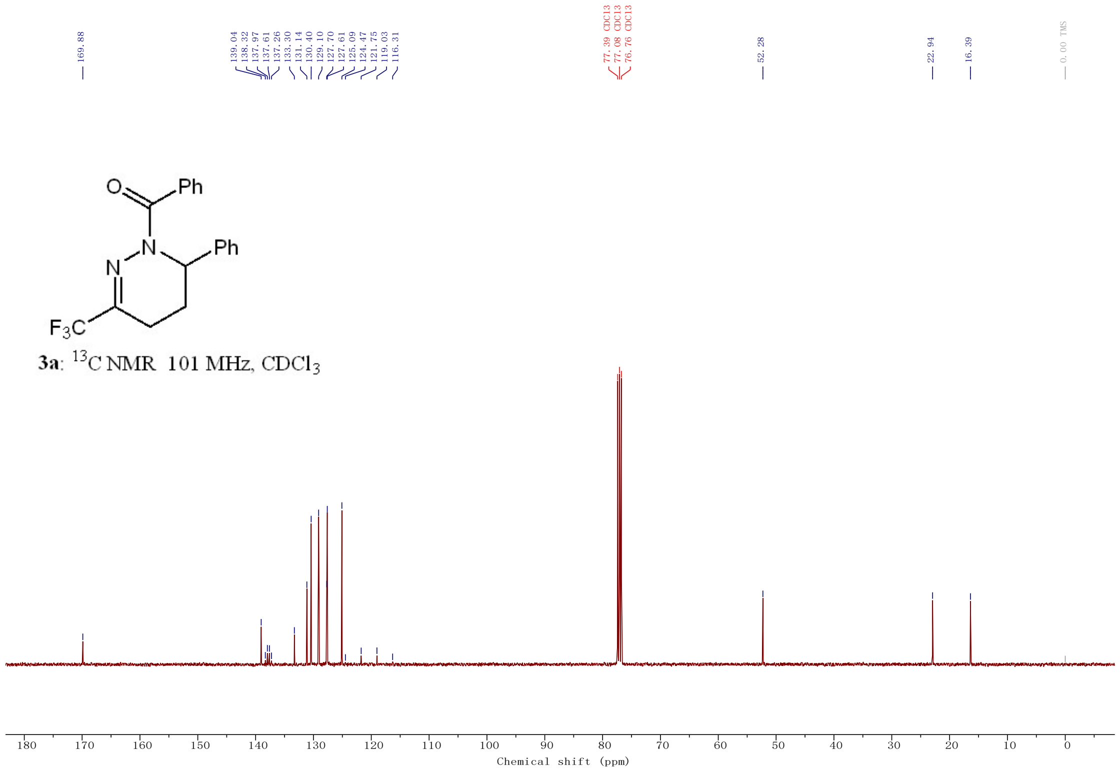

3a: ^{13}C NMR 101 MHz, CDCl$_3$

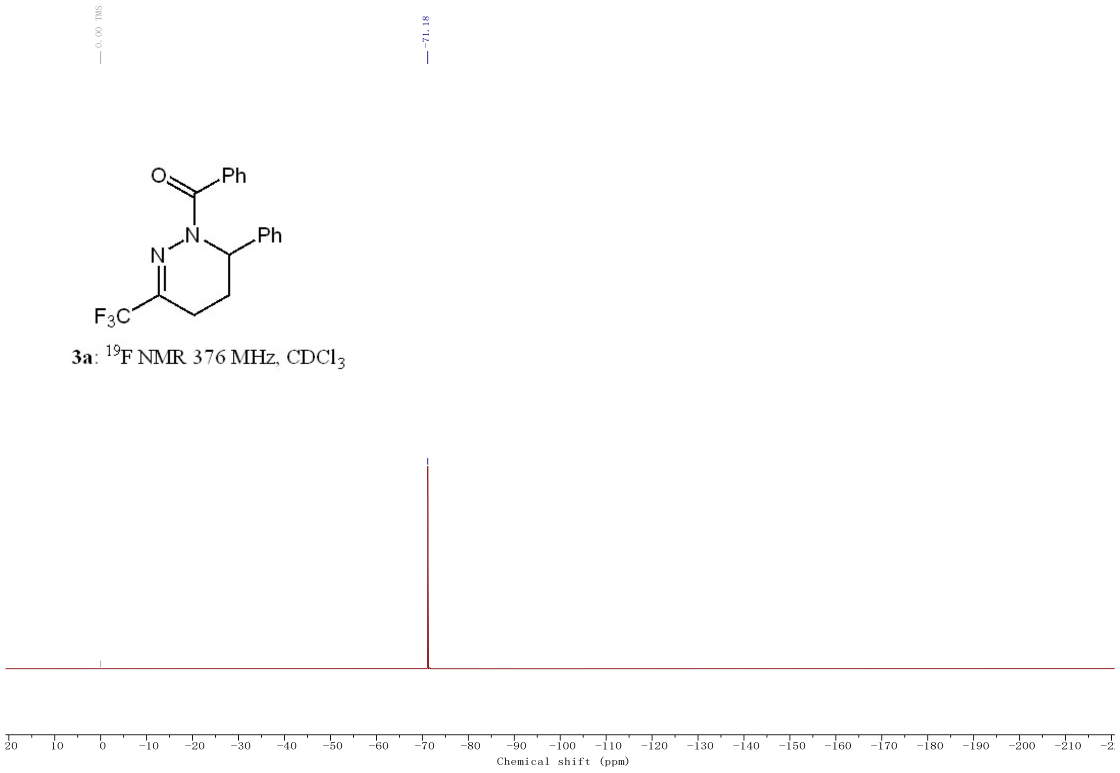

NMR copies of compound **3b:**

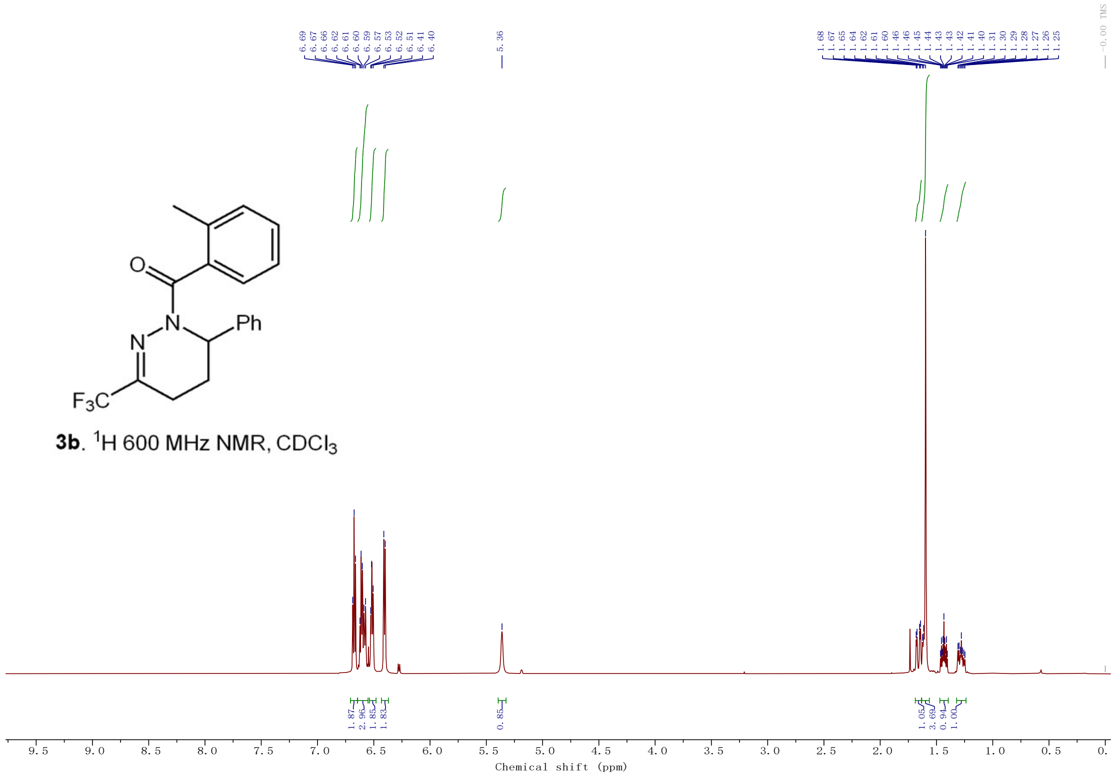

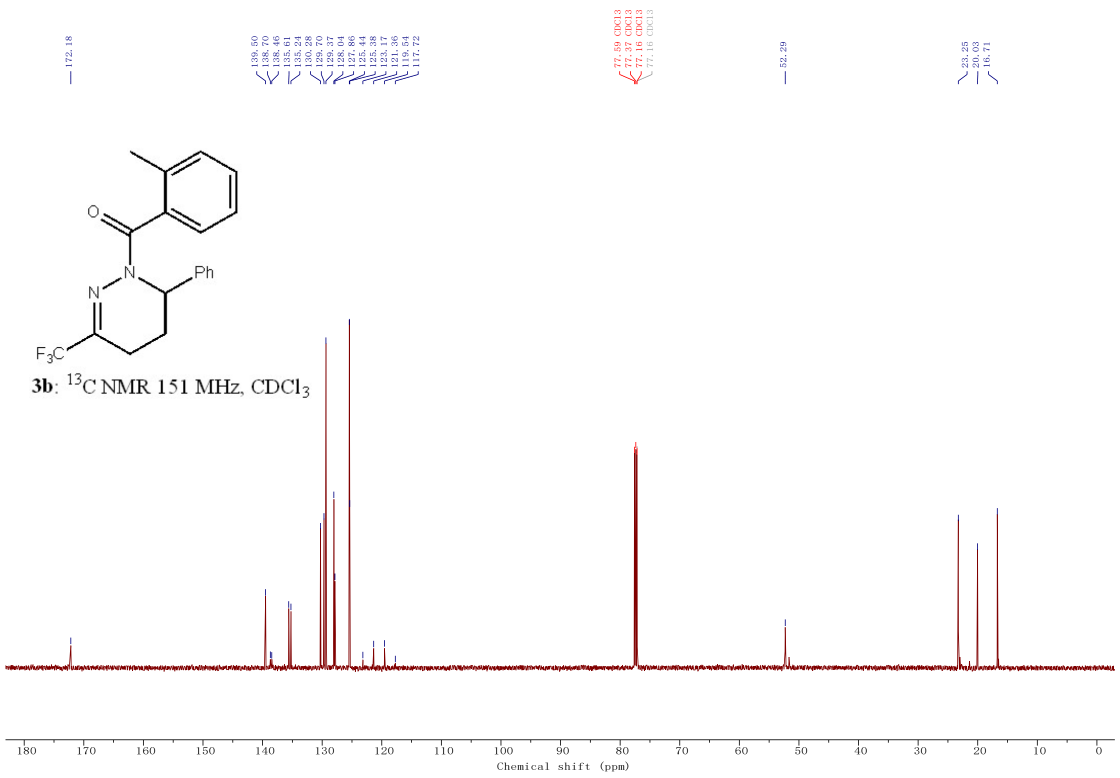
172.18
139.50
138.70
138.46
135.61
135.24
130.28
129.70
129.37
128.04
127.86
125.44
125.38
123.17
121.36
119.54
117.72
77.59 CDCl3
77.37 CDCl3
77.16 CDCl3
77.16 CDCl3
52.29
23.25
20.03
16.71
O
N
N
Ph
F3C
3b: 13C NMR 151 MHz, CDCl3
Chemical shift (ppm)

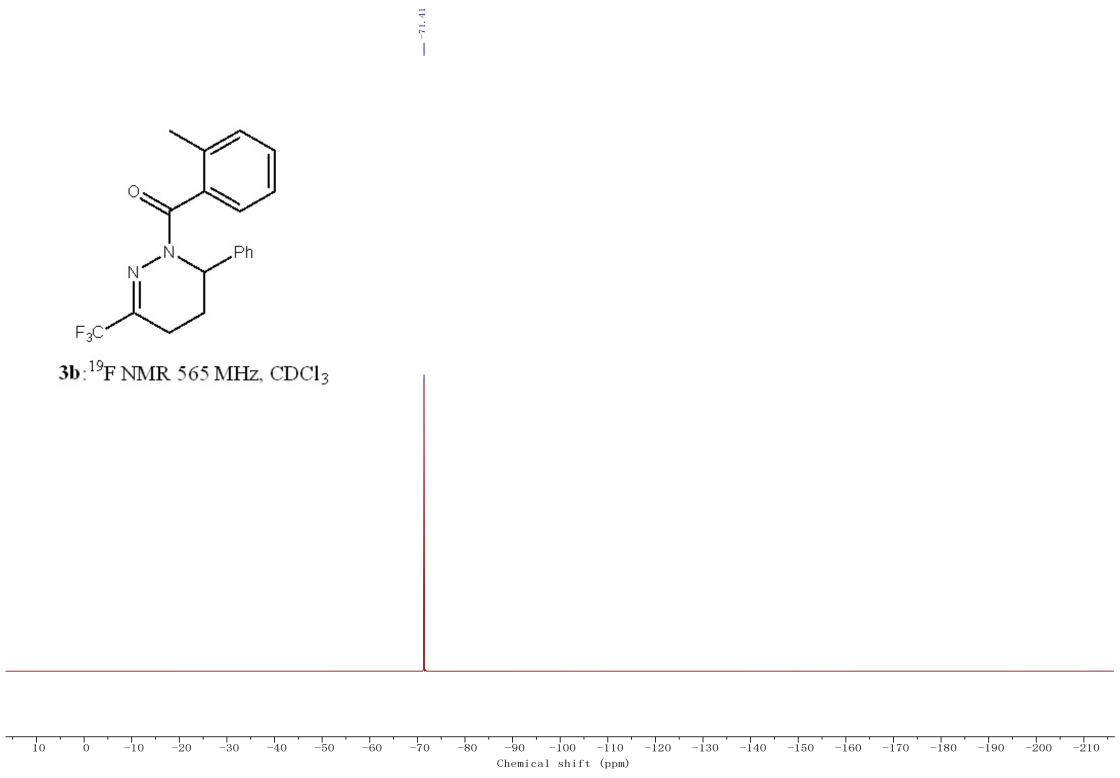
-71.41
O
N
N
Ph
F3C
3b: 19F NMR 565 MHz, CDCl3
Chemical shift (ppm)

NMR copies of compound **3c:**

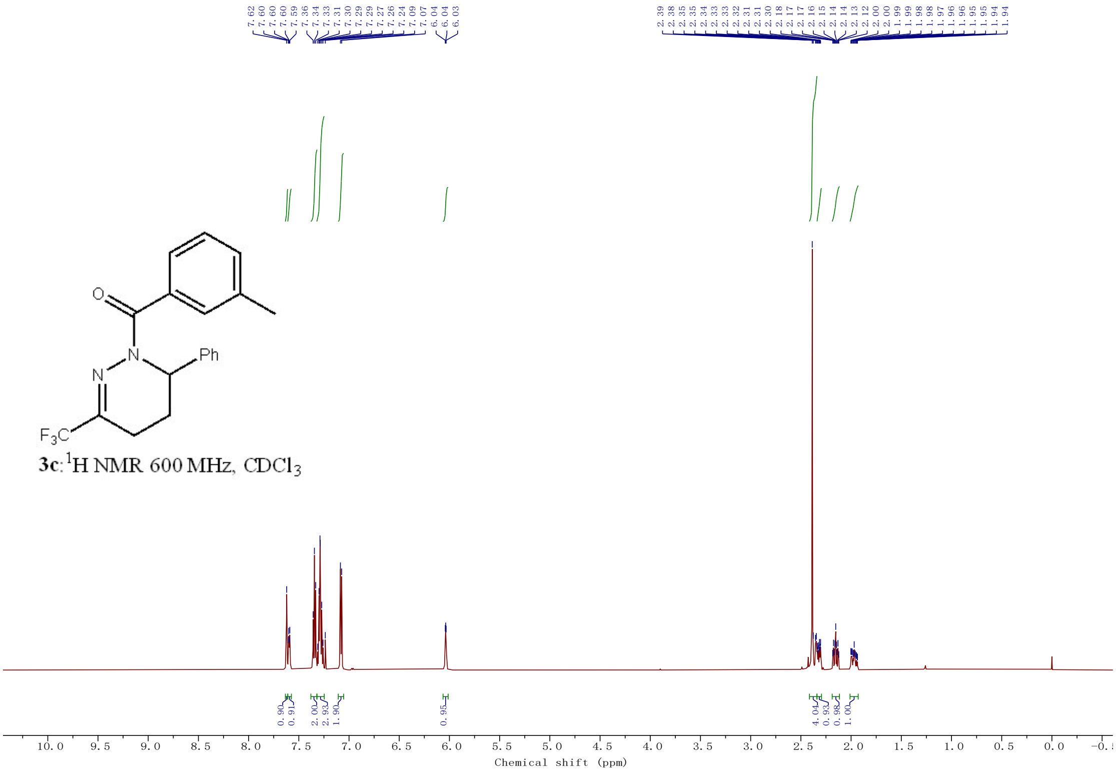

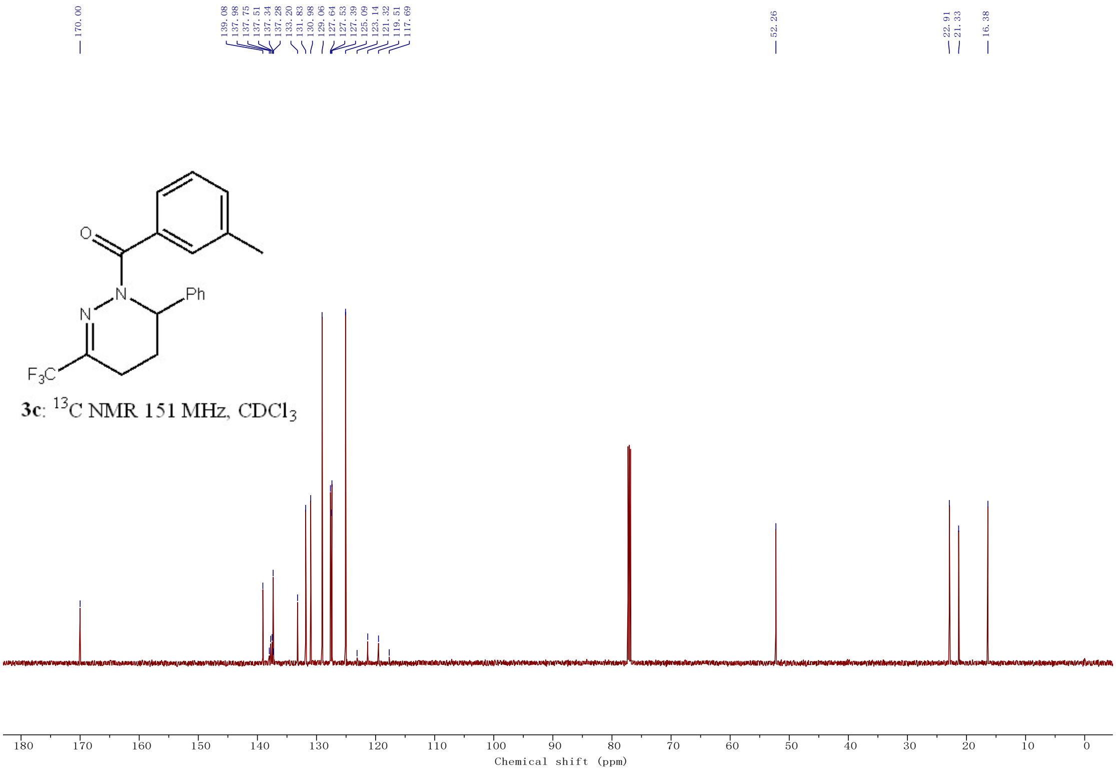

3c: ^{13}C NMR 151 MHz, $CDCl_3$

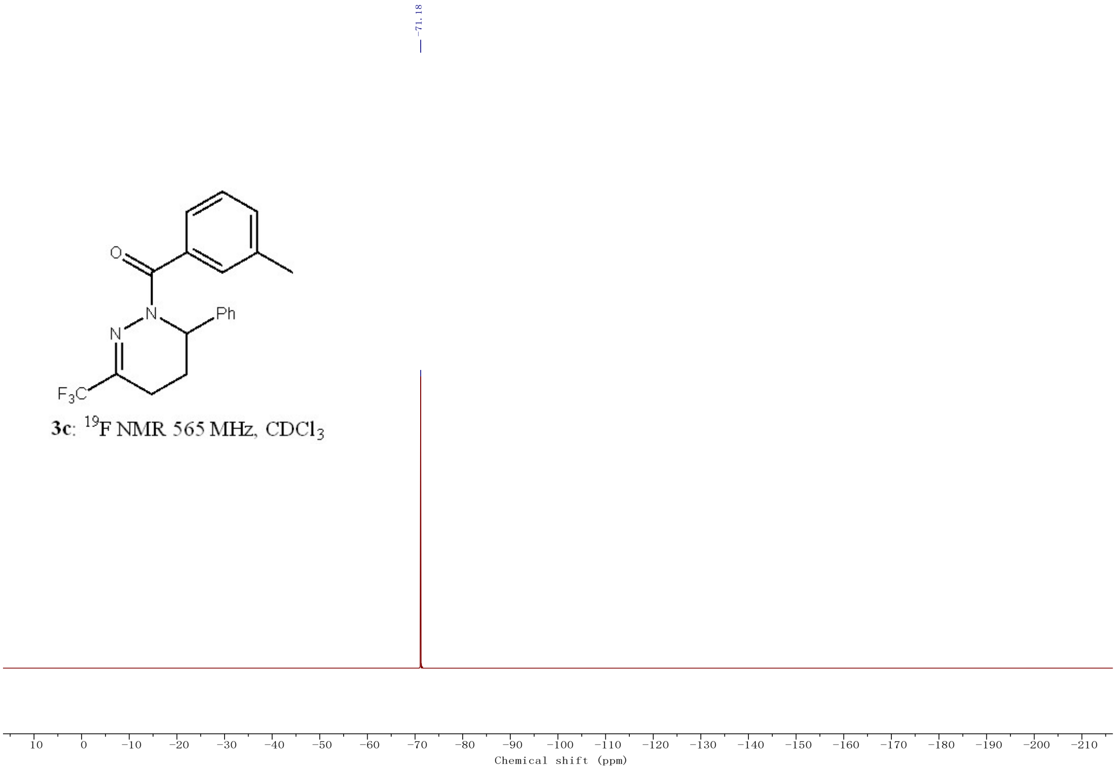

3c: ^{19}F NMR 565 MHz, $CDCl_3$

NMR copies of compound **3d:**

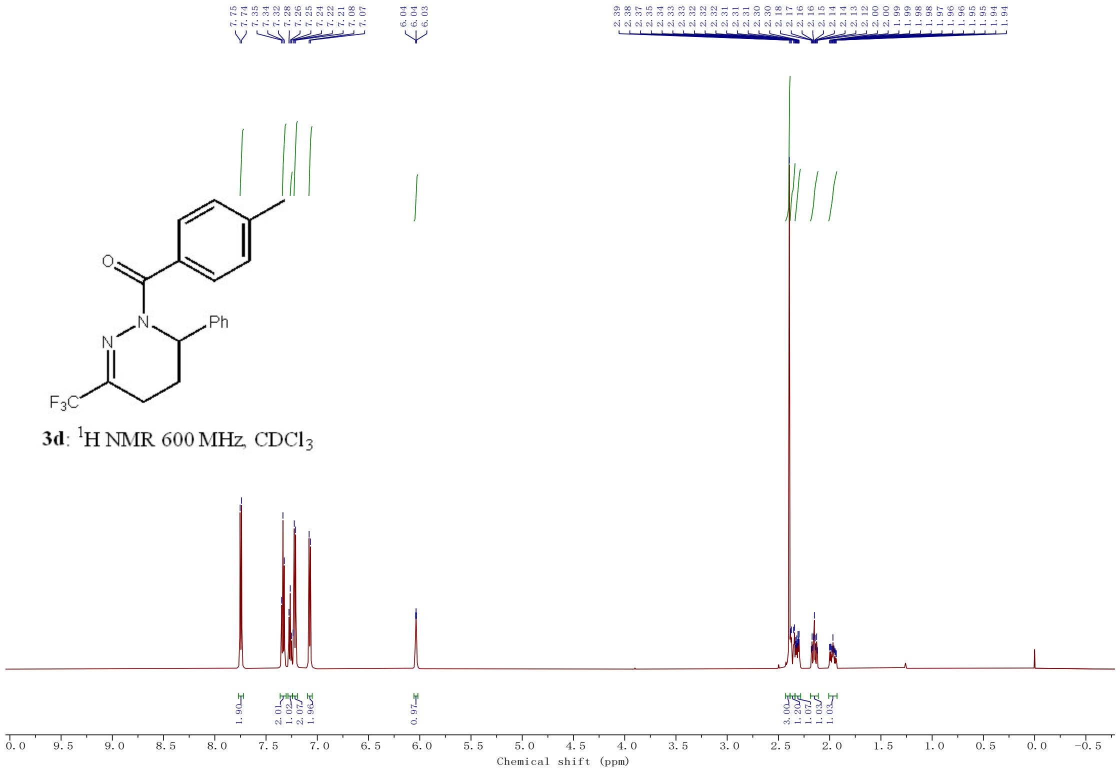
7.75 7.74 7.35 7.34 7.32 7.28 7.26 7.25 7.24 7.22 7.21 7.08 7.07
6.04 6.04 6.03
2.39 2.38 2.37 2.35 2.34 2.33 2.33 2.32 2.32 2.31 2.31 2.31 2.30 2.30 2.18 2.17 2.16 2.16 2.15 2.14 2.14 2.13 2.12 2.00 1.99 1.99 1.98 1.98 1.97 1.96 1.96 1.95 1.95 1.94 1.94
O
N
N
Ph
F3C
3d: 1H NMR 600 MHz, CDCl3
1.90
2.01 1.02 2.07 1.96
0.97
3.00 1.20 1.07 1.03 1.03
Chemical shift (ppm)

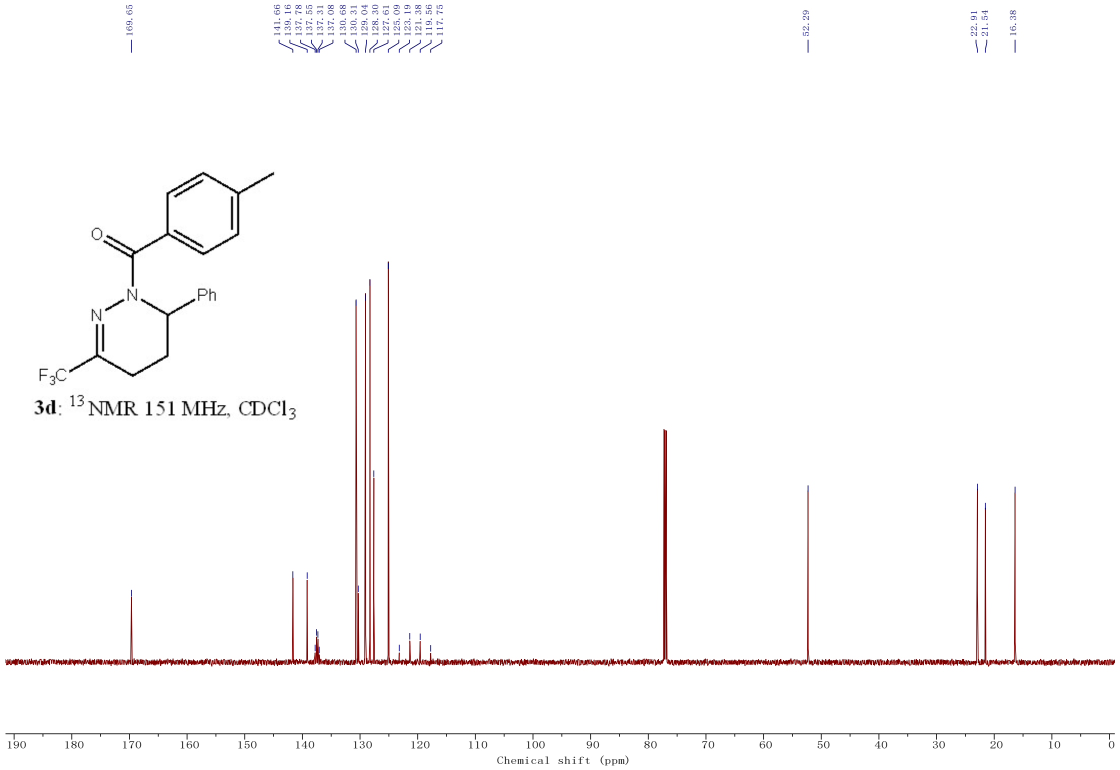
169.65
141.66 139.16 137.78 137.55 137.31 137.08 130.68 130.31 129.04 128.30 127.61 125.09 123.19 121.38 119.56 117.75
52.29
22.91 21.54 16.38
O
N
N
Ph
F3C
3d: 13NMR 151 MHz, CDCl3
Chemical shift (ppm)

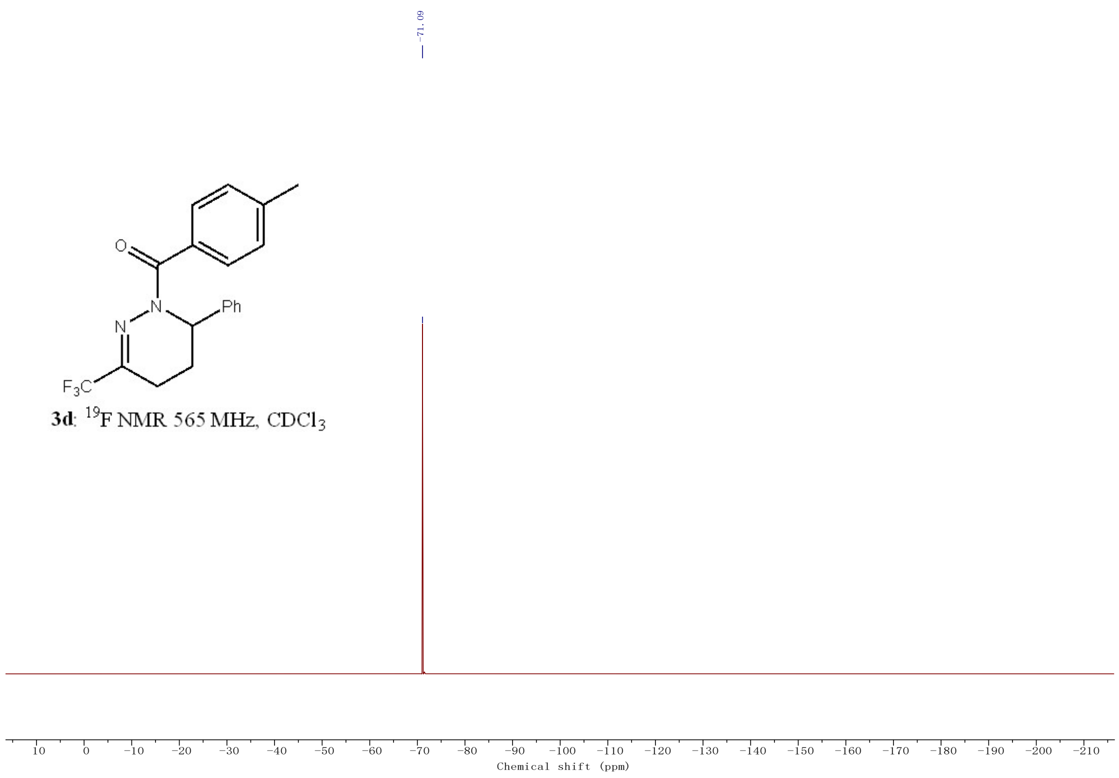

NMR copies of compound **3e:**

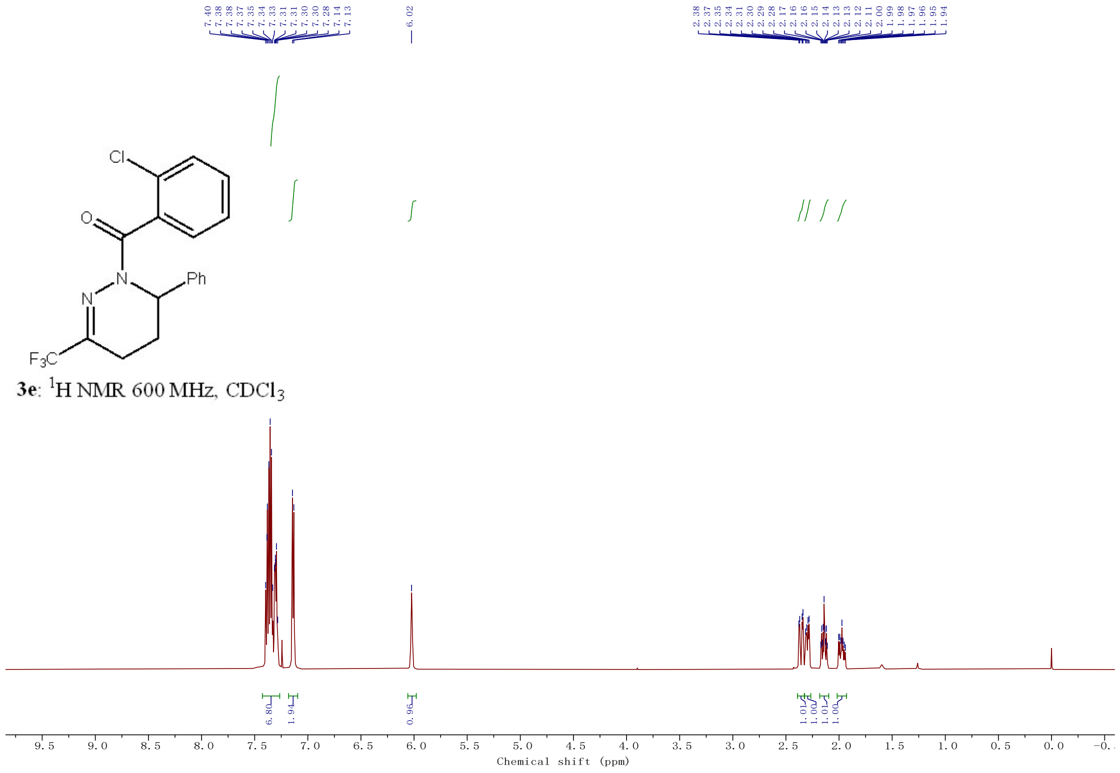

3e: ^{1}H NMR 600 MHz, $CDCl_3$

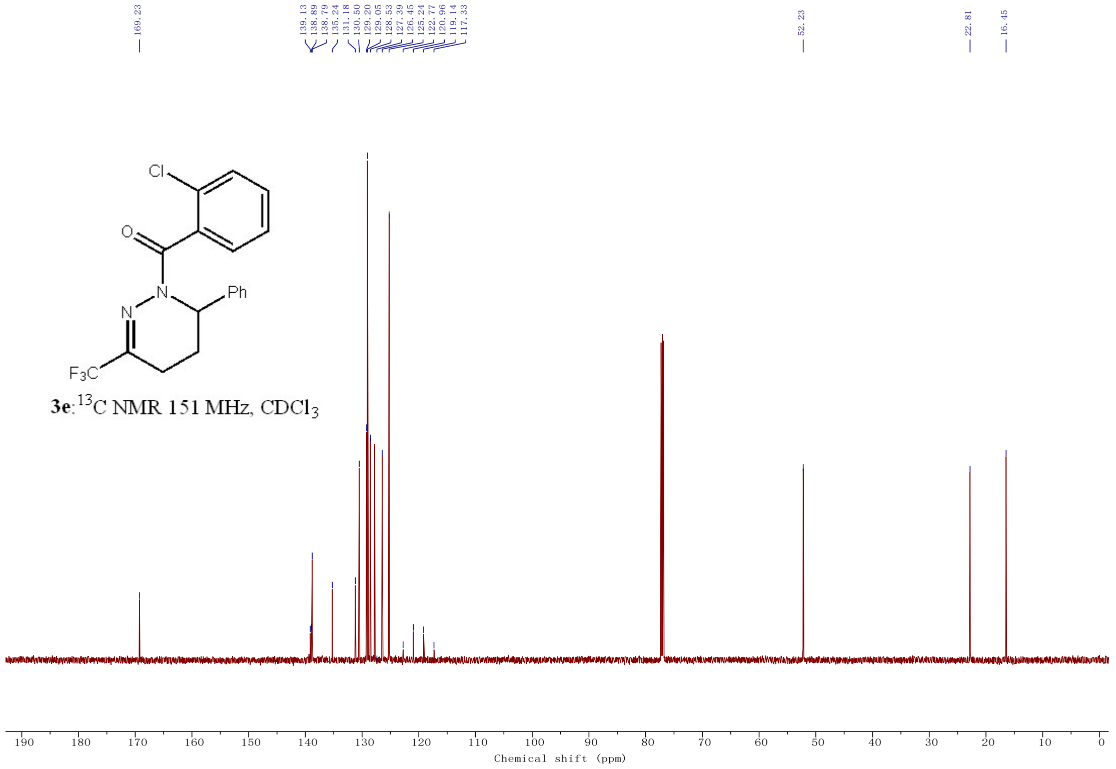

3e: ^{13}C NMR 151 MHz, $CDCl_3$

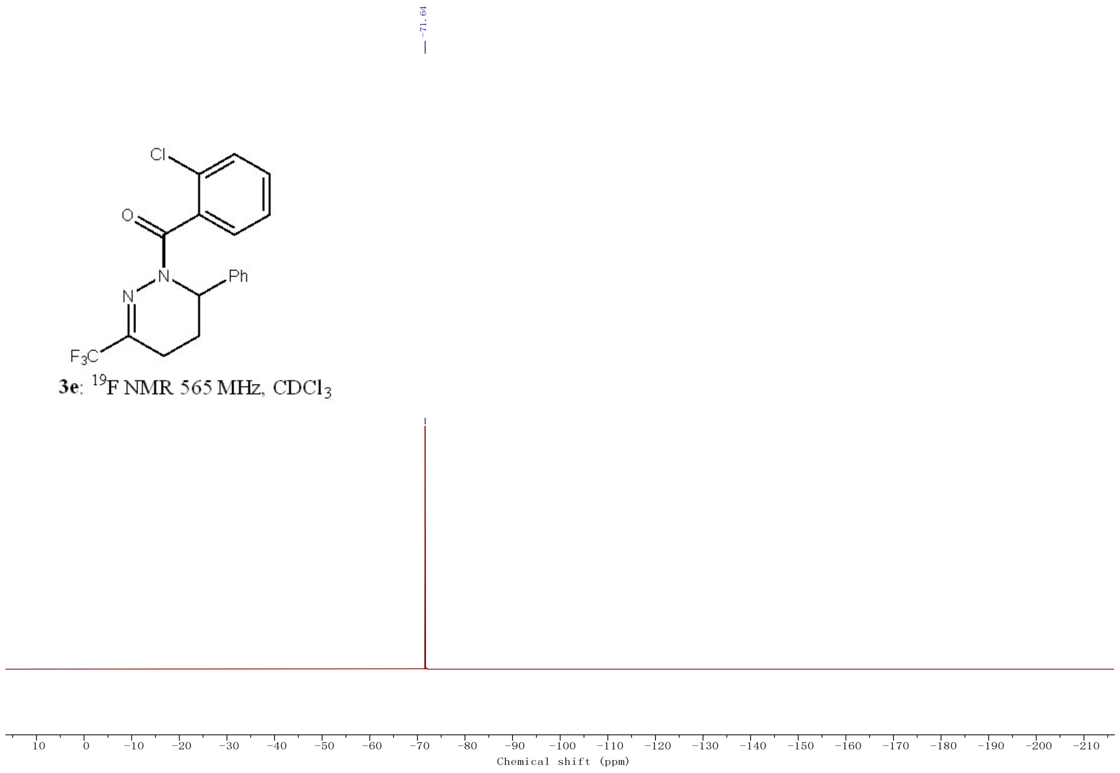

NMR copies of compound **3f:**

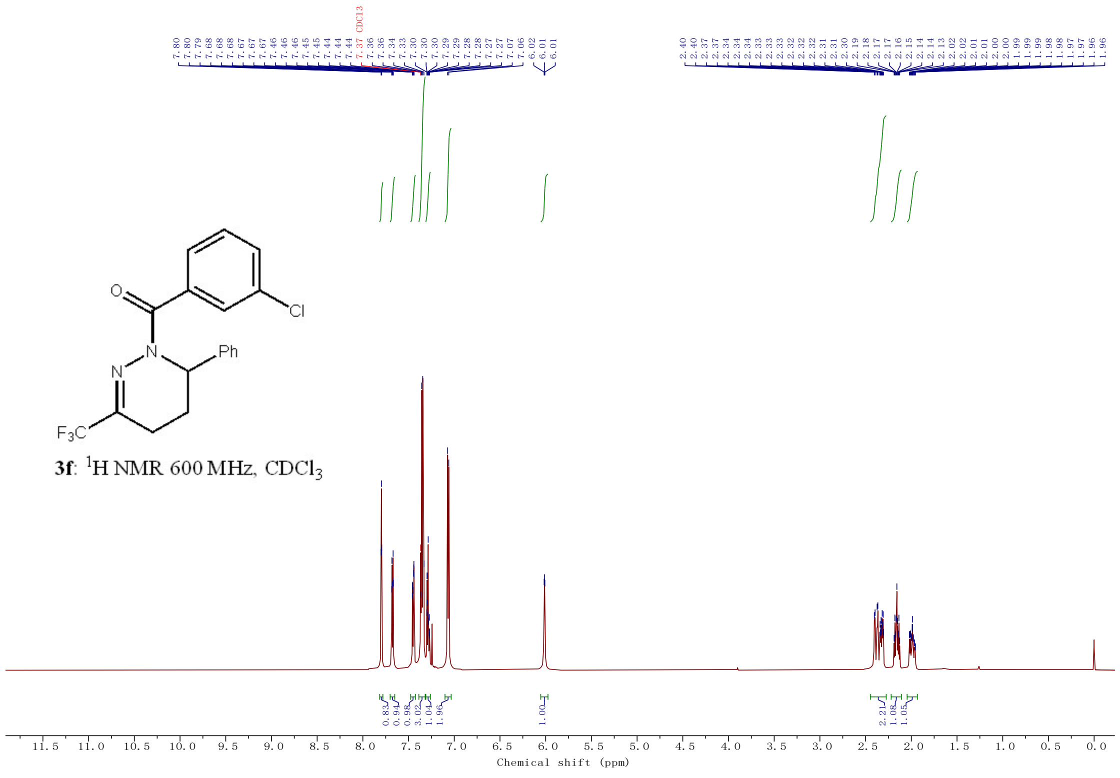

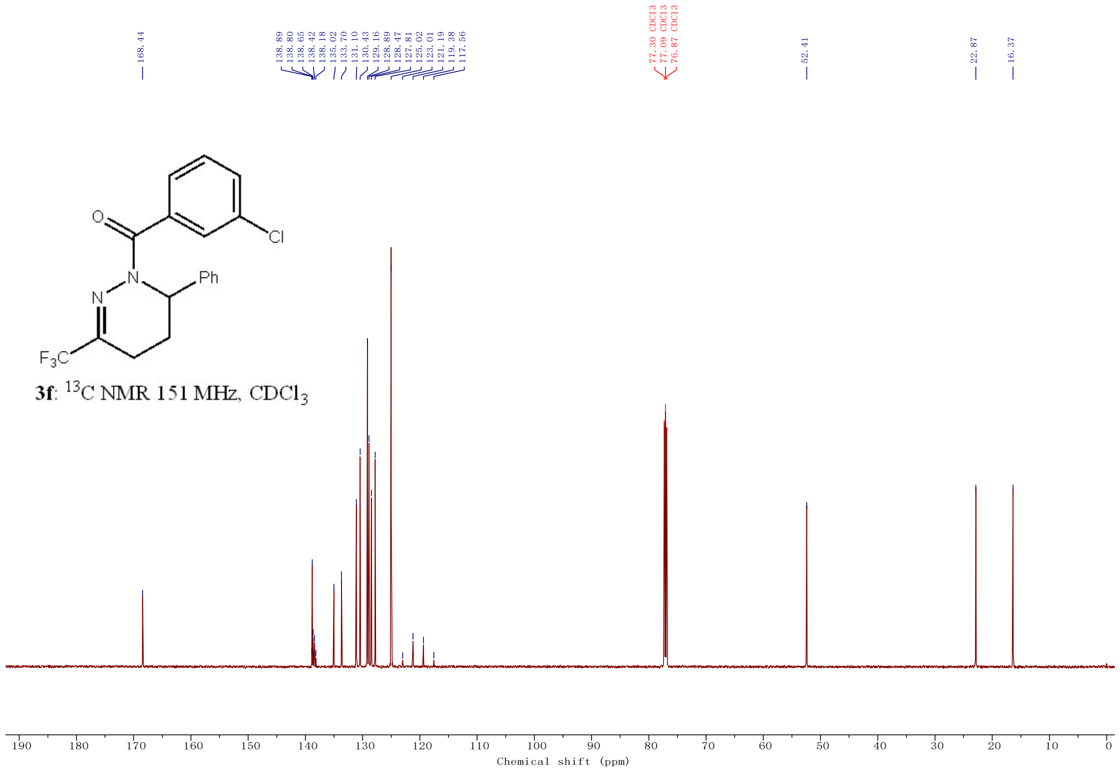
168.44
138.89
138.80
138.65
138.42
138.18
135.02
133.70
131.10
130.43
129.16
128.89
128.47
127.81
125.02
123.01
121.19
119.38
117.56
77.30 CDCl3
77.09 CDCl3
76.87 CDCl3
52.41
22.87
16.37
O
Cl
N
N
Ph
F_3C
3f: ^{13}C NMR 151 MHz, $CDCl_3$
Chemical shift (ppm)

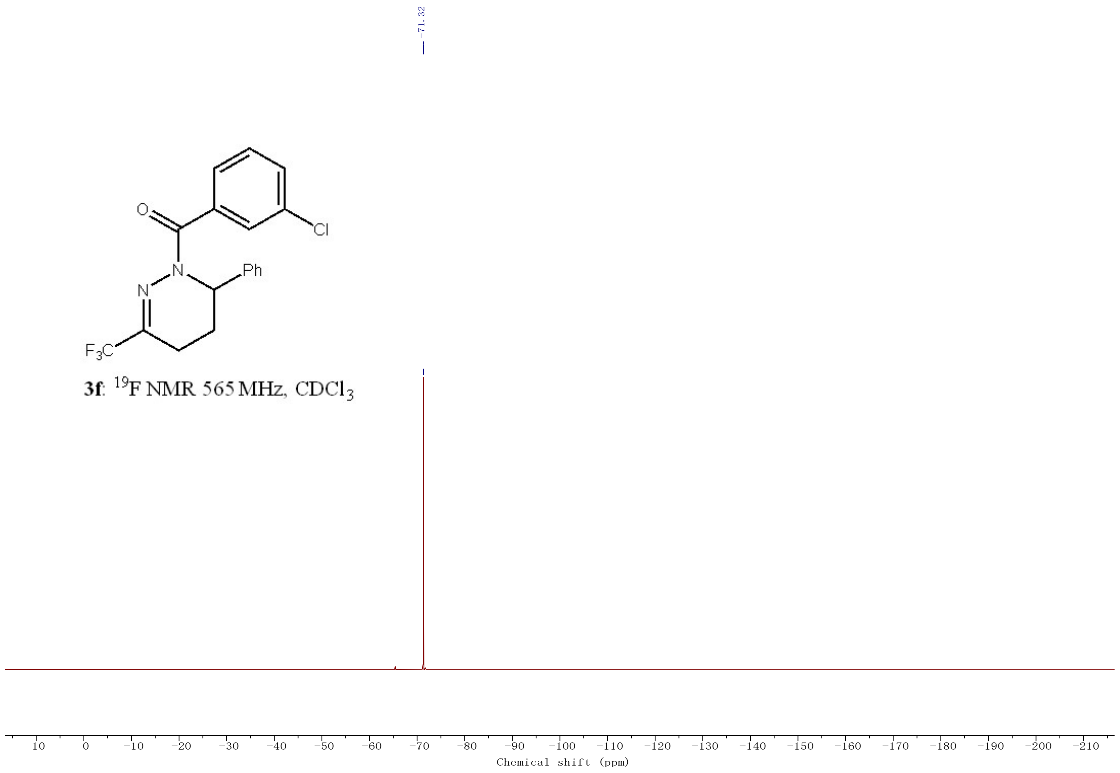
−71.32
O
Cl
N
N
Ph
F_3C
3f: ^{19}F NMR 565 MHz, $CDCl_3$
Chemical shift (ppm)

NMR copies of compound **3g:**

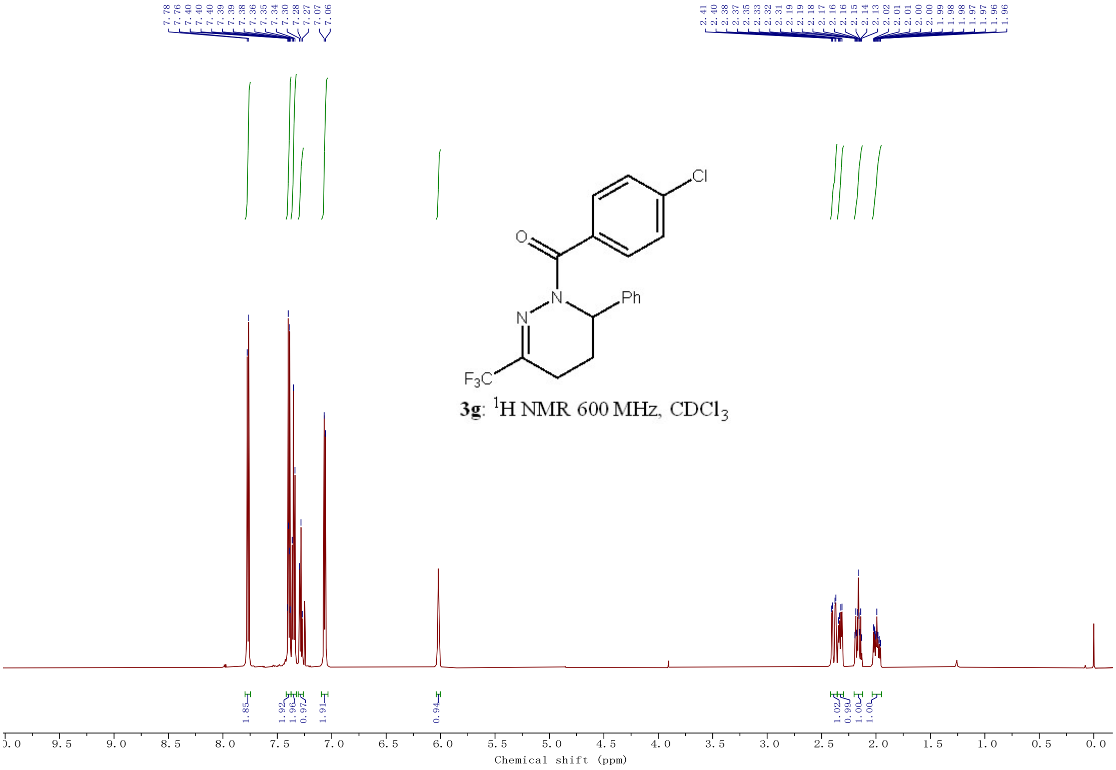

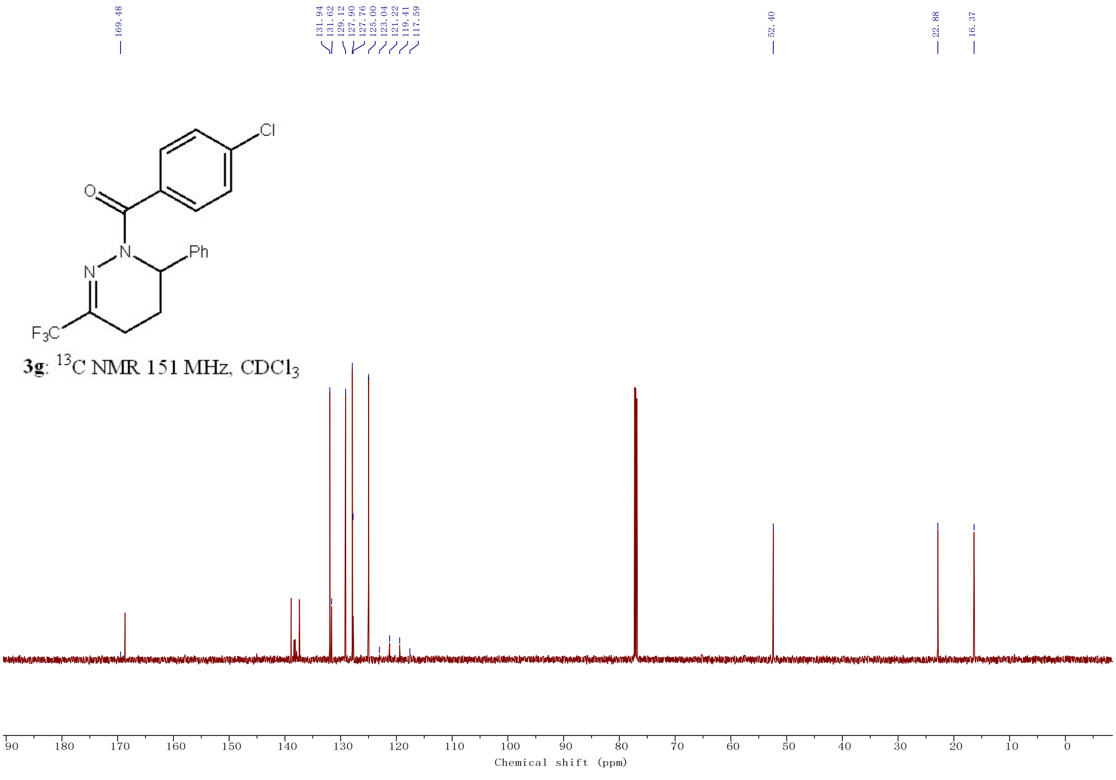

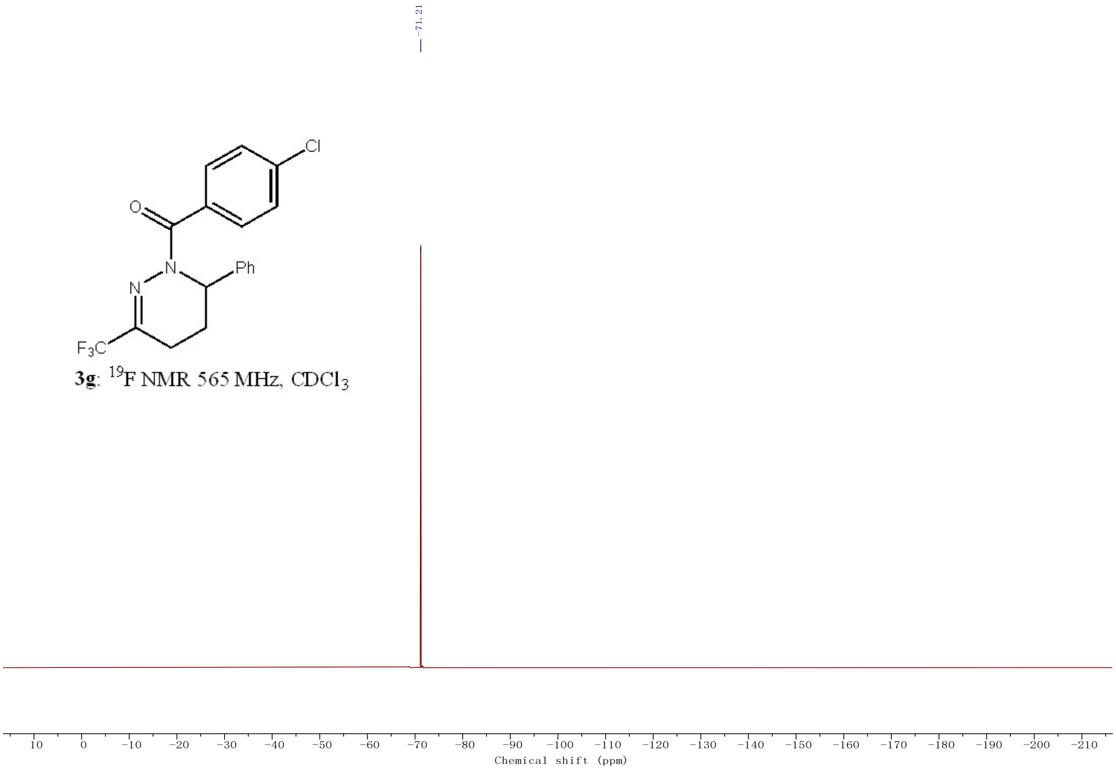

NMR copies of compound **3h:**

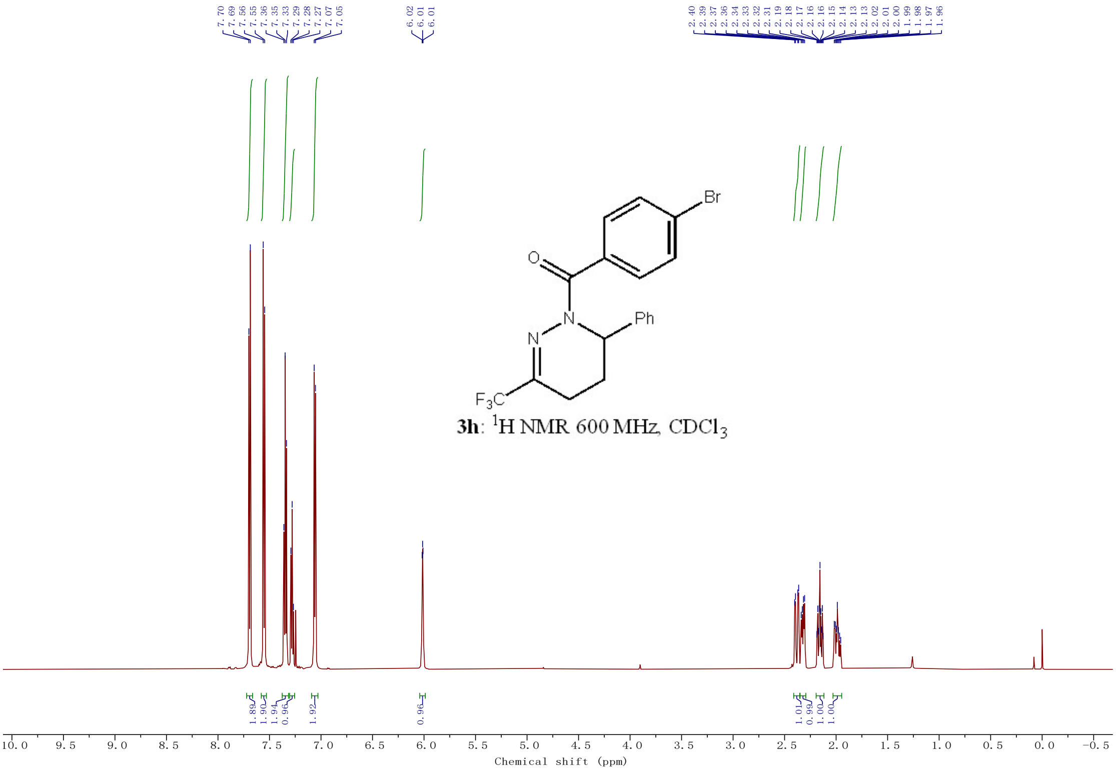

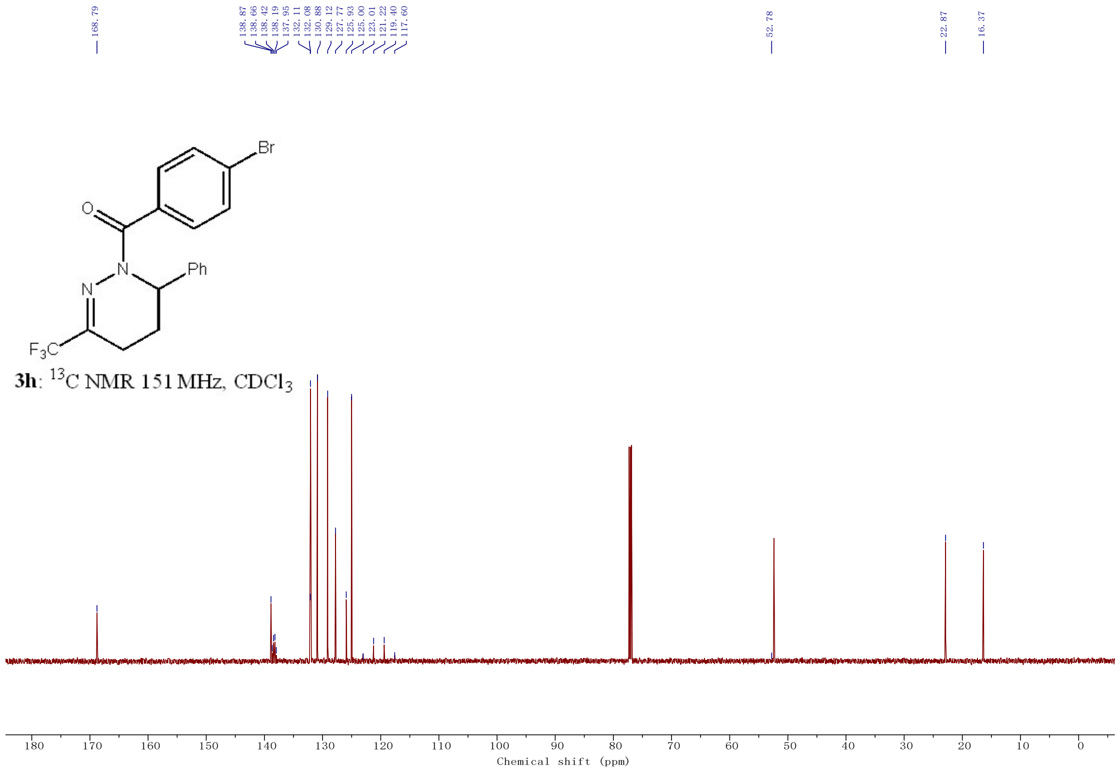
168.79
138.87
138.66
138.42
138.19
137.95
132.11
132.08
130.88
129.12
127.77
125.93
125.00
123.01
121.22
119.40
117.60
52.78
22.87
16.37
Br
O
N
N
Ph
F3C
3h: 13C NMR 151 MHz, CDCl3
180 170 160 150 140 130 120 110 100 90 80 70 60 50 40 30 20 10 0
Chemical shift (ppm)

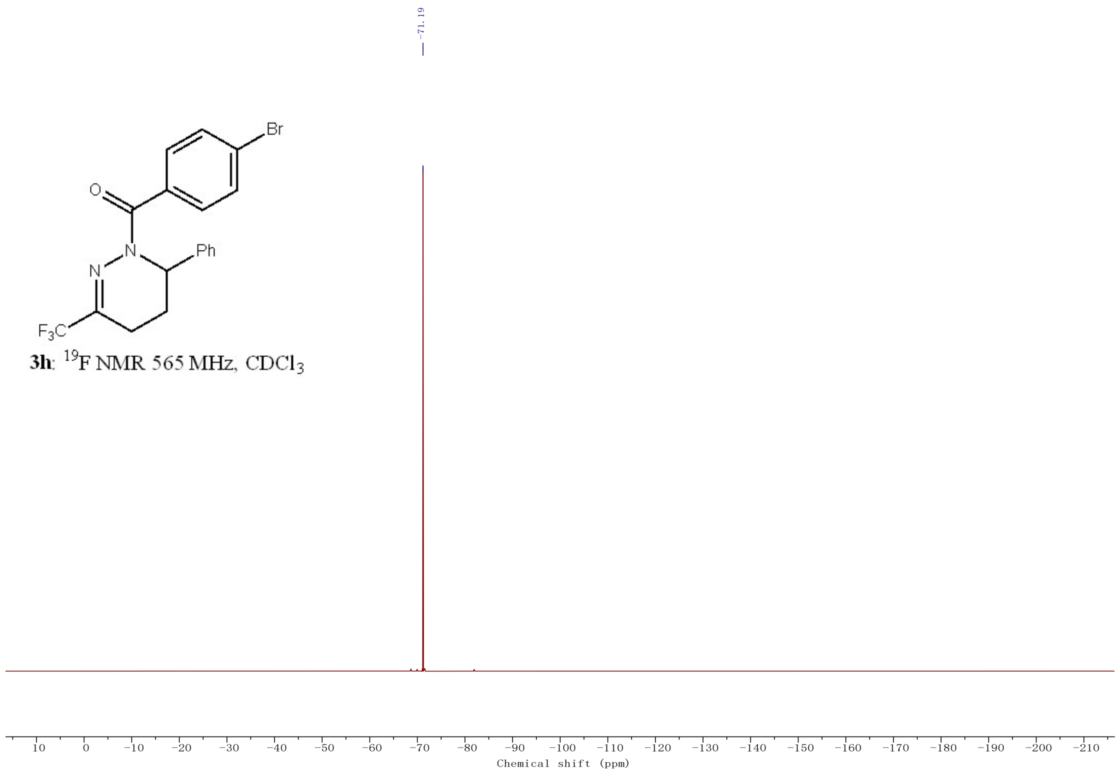
-71.19
Br
O
N
N
Ph
F3C
3h: 19F NMR 565 MHz, CDCl3
10 0 -10 -20 -30 -40 -50 -60 -70 -80 -90 -100 -110 -120 -130 -140 -150 -160 -170 -180 -190 -200 -210
Chemical shift (ppm)

NMR copies of compound **3i:**

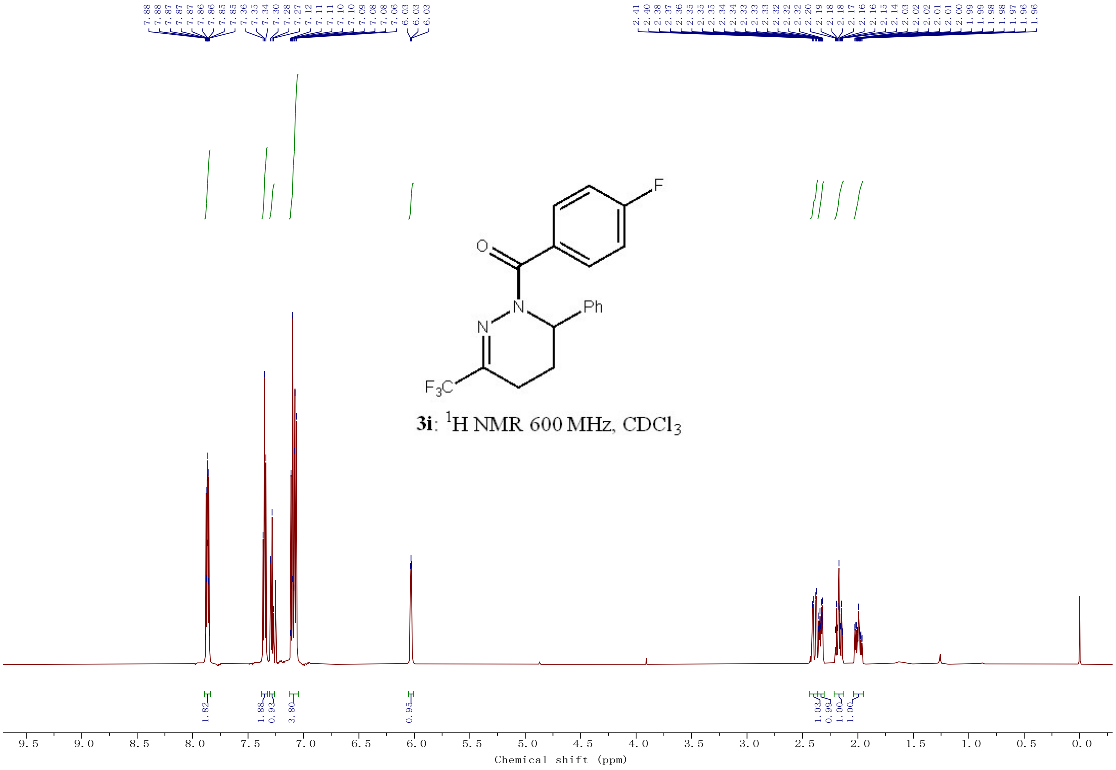

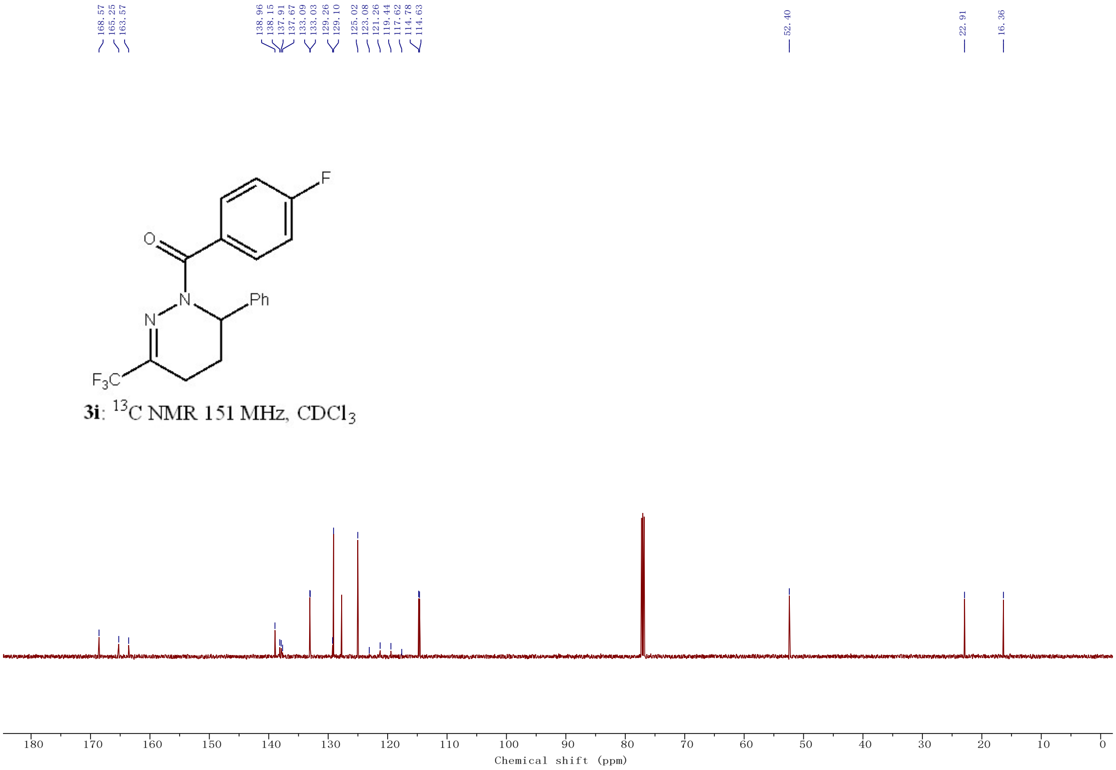

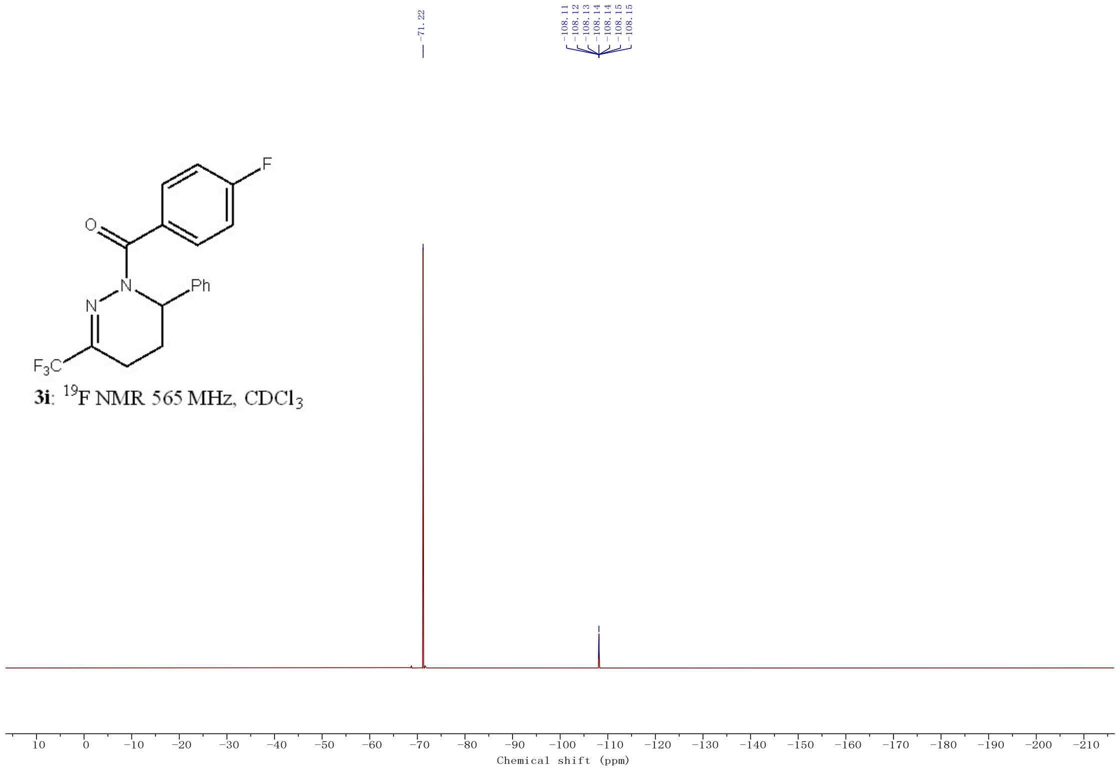

NMR copies of compound **3j:**

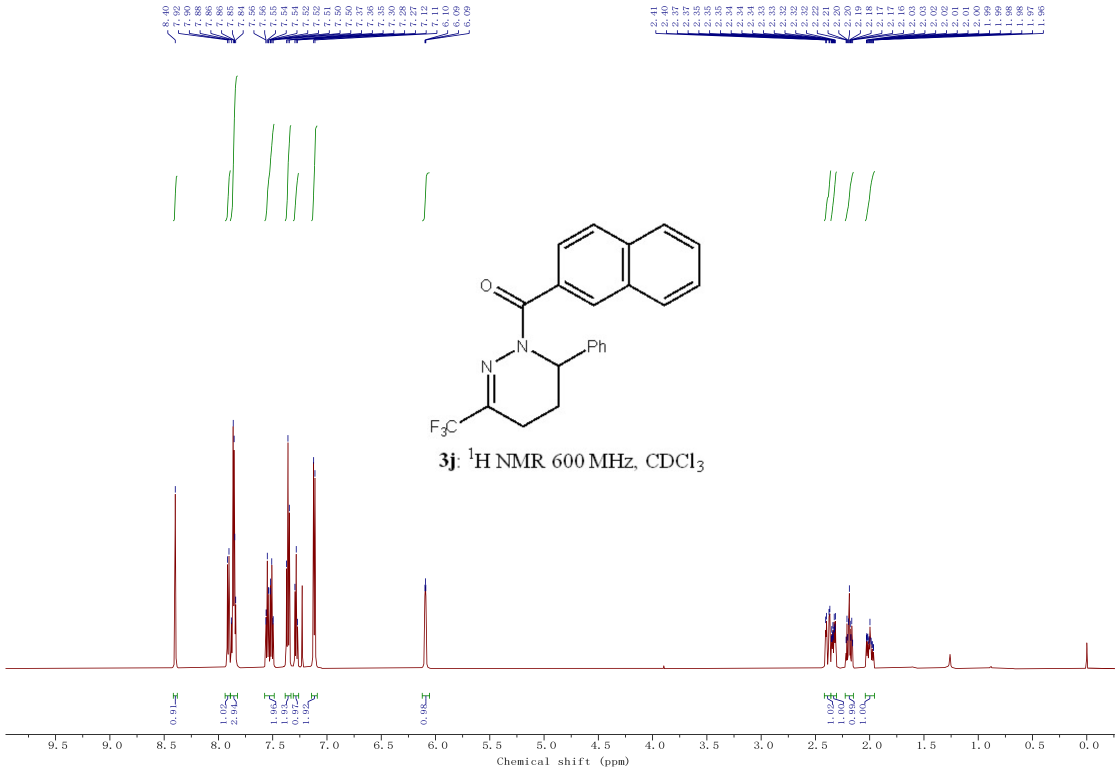

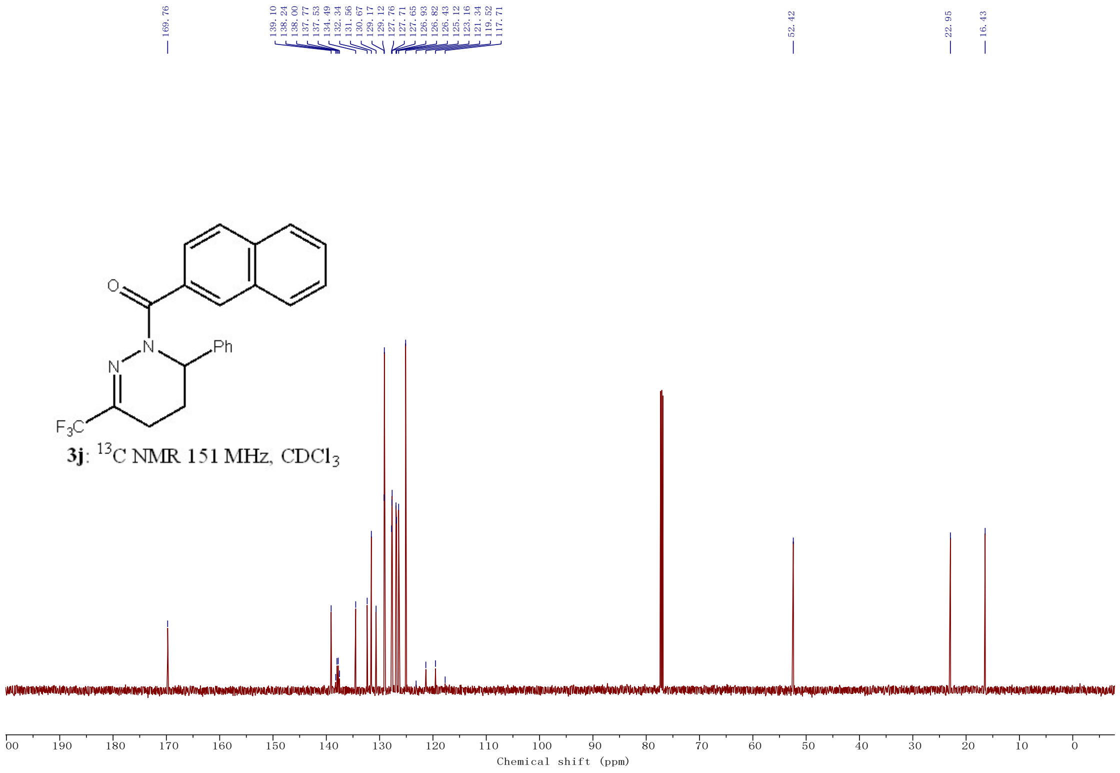

3j: ^{13}C NMR 151 MHz, $CDCl_3$

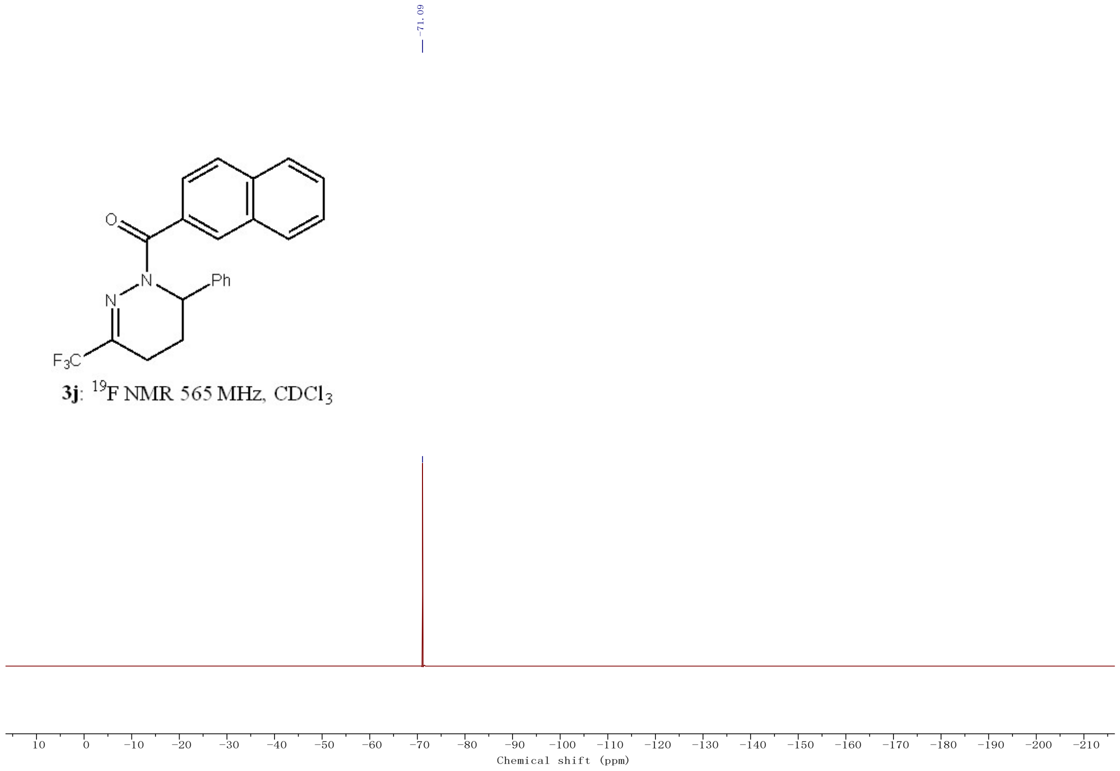

3j: ^{19}F NMR 565 MHz, $CDCl_3$

NMR copies of compound **3k:**

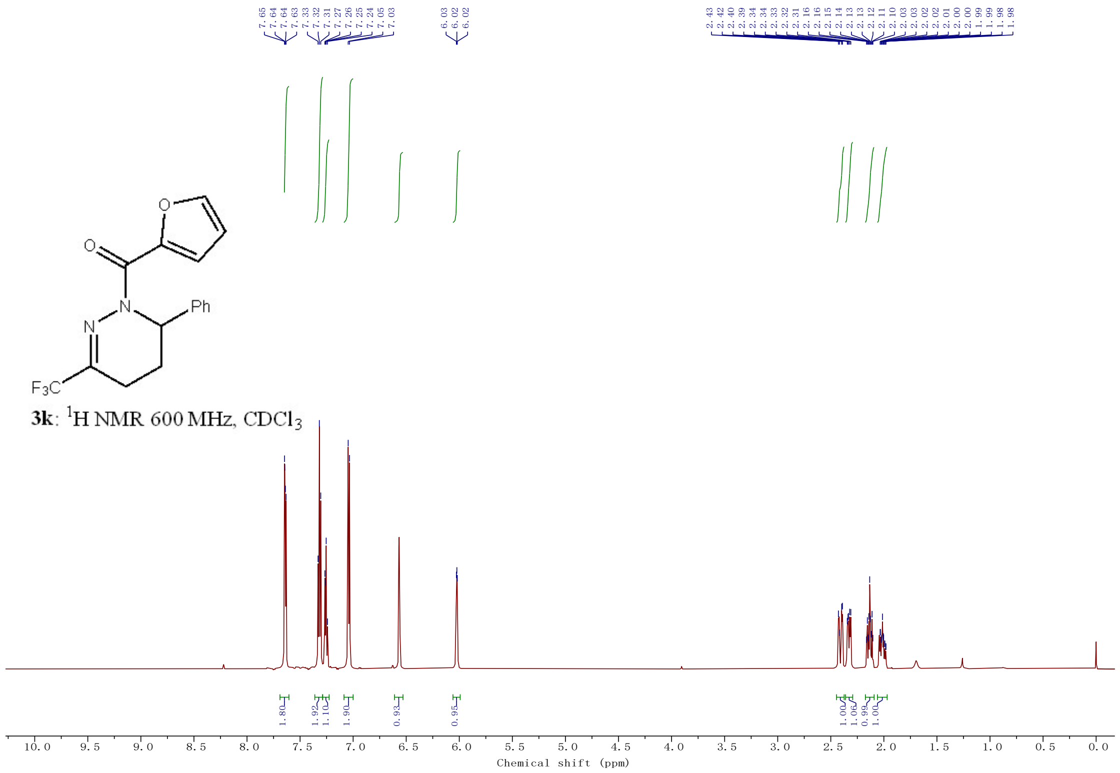

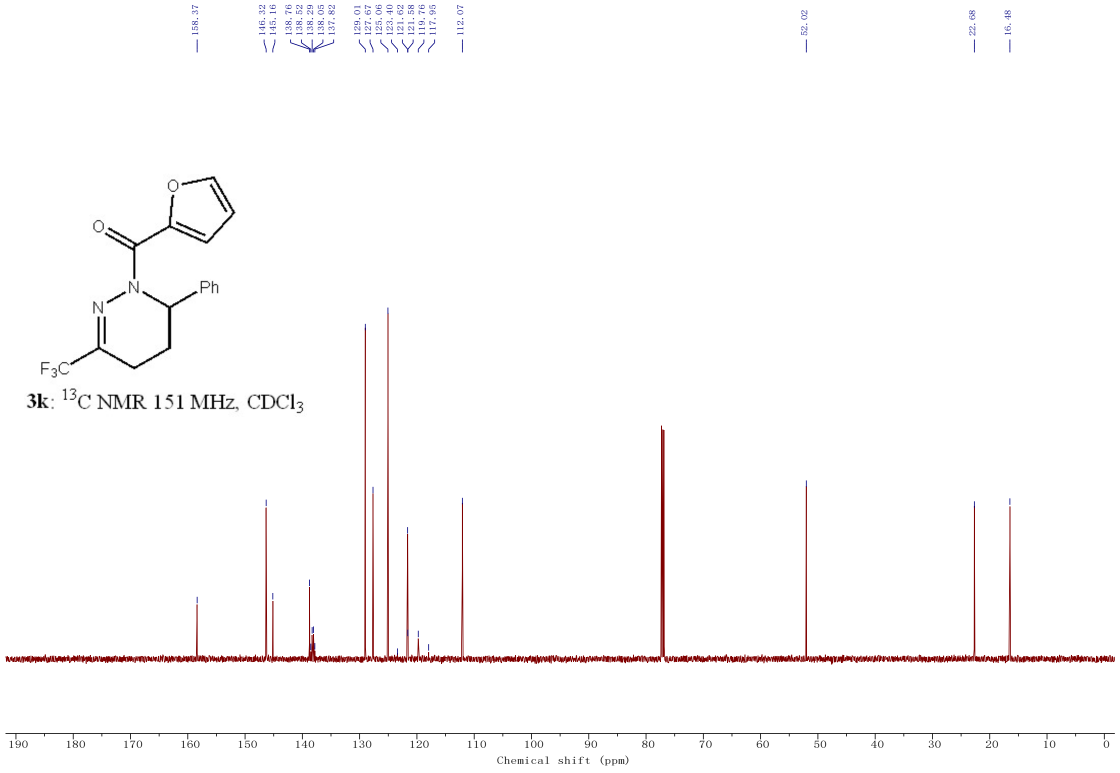

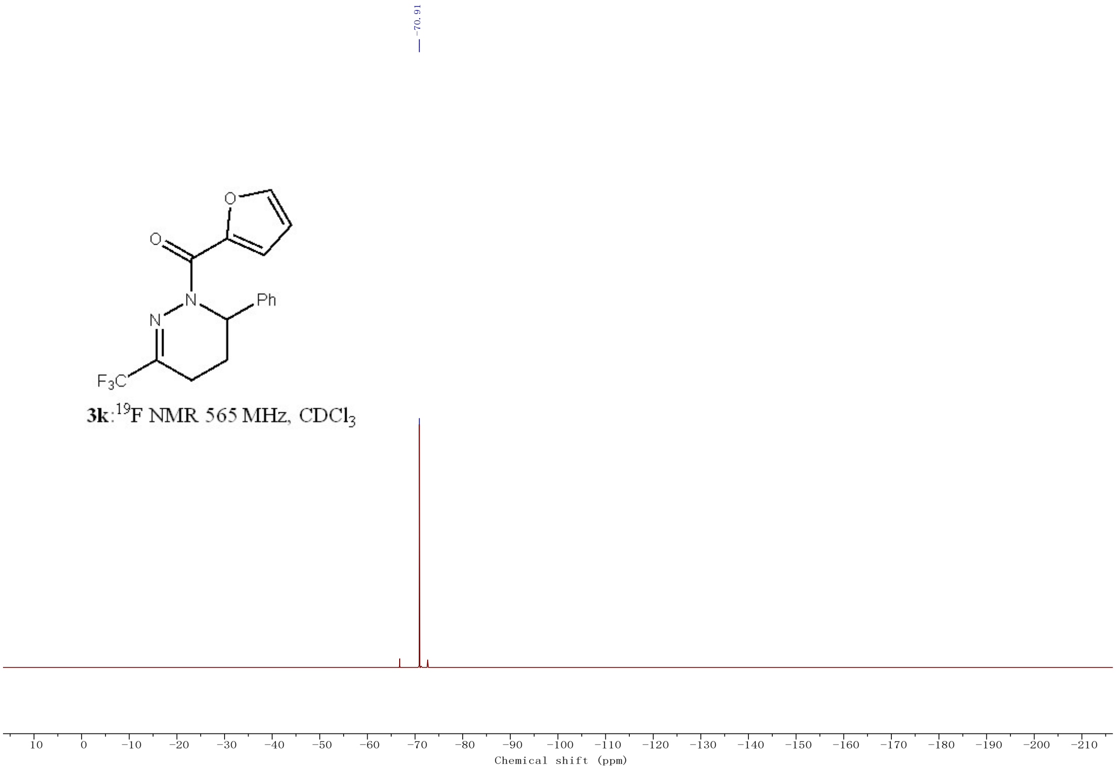

3k:^{19}F NMR 565 MHz, $CDCl_3$

NMR copies of compound **3l:**

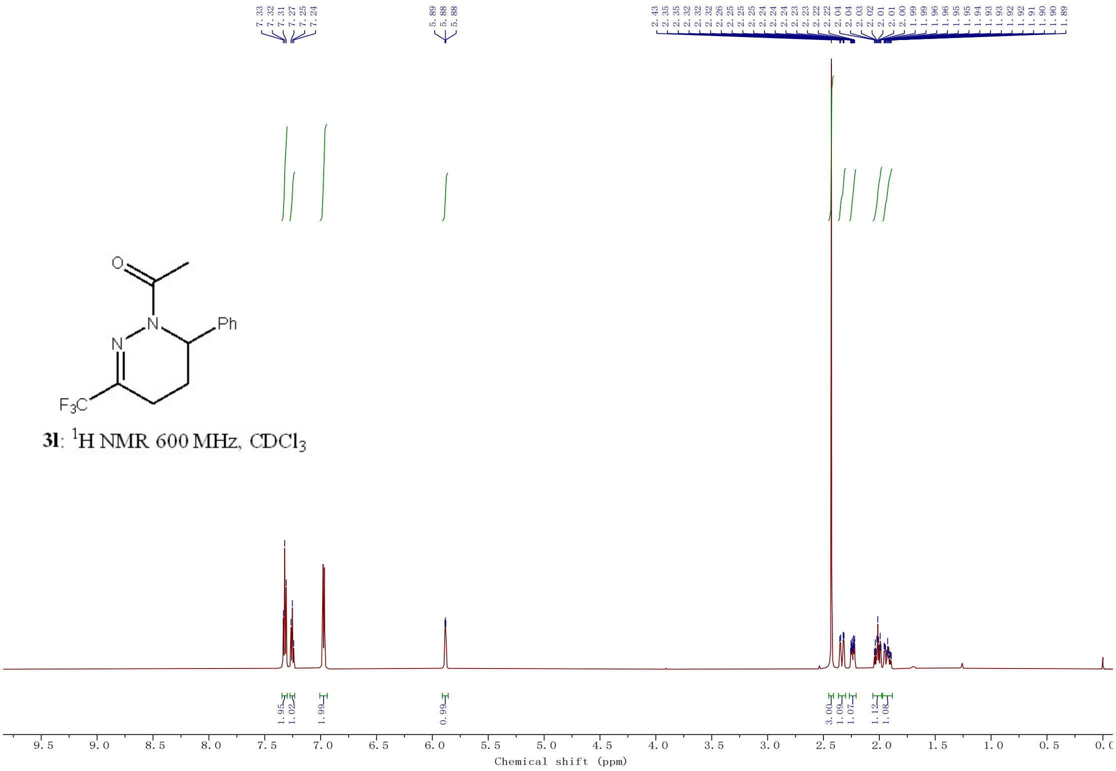

3l: ^{1}H NMR 600 MHz, $CDCl_3$

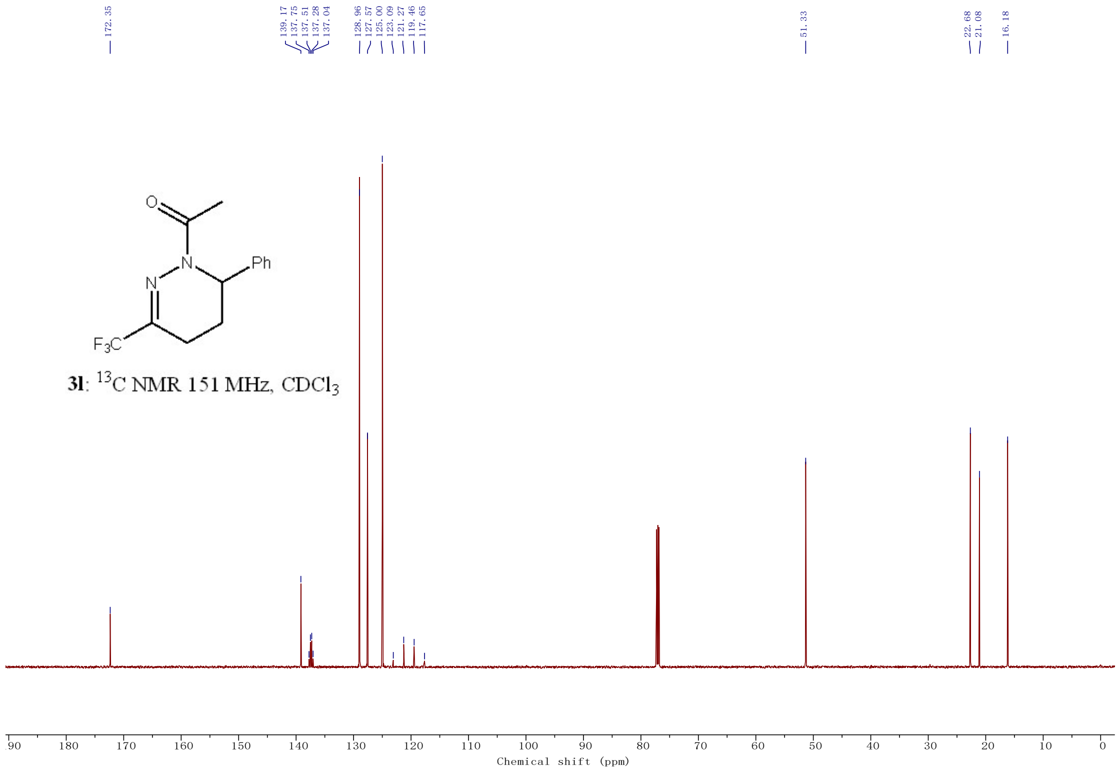
172.35
139.17
137.75
137.51
137.28
137.04
128.96
127.57
125.00
123.09
121.27
119.46
117.65
51.33
22.68
21.08
16.18
O
N
N
Ph
F3C
3l: 13C NMR 151 MHz, CDCl3
Chemical shift (ppm)

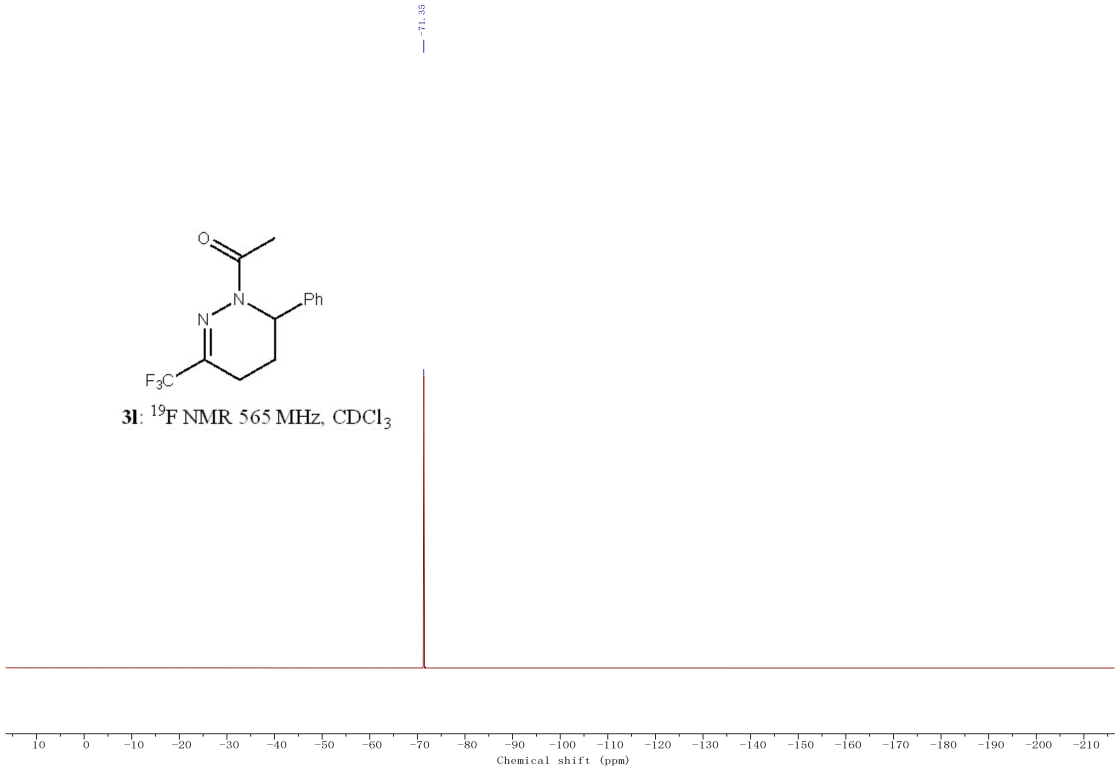
−71.35
O
N
N
Ph
F3C
3l: 19F NMR 565 MHz, CDCl3
Chemical shift (ppm)

NMR copies of compound **3m:**

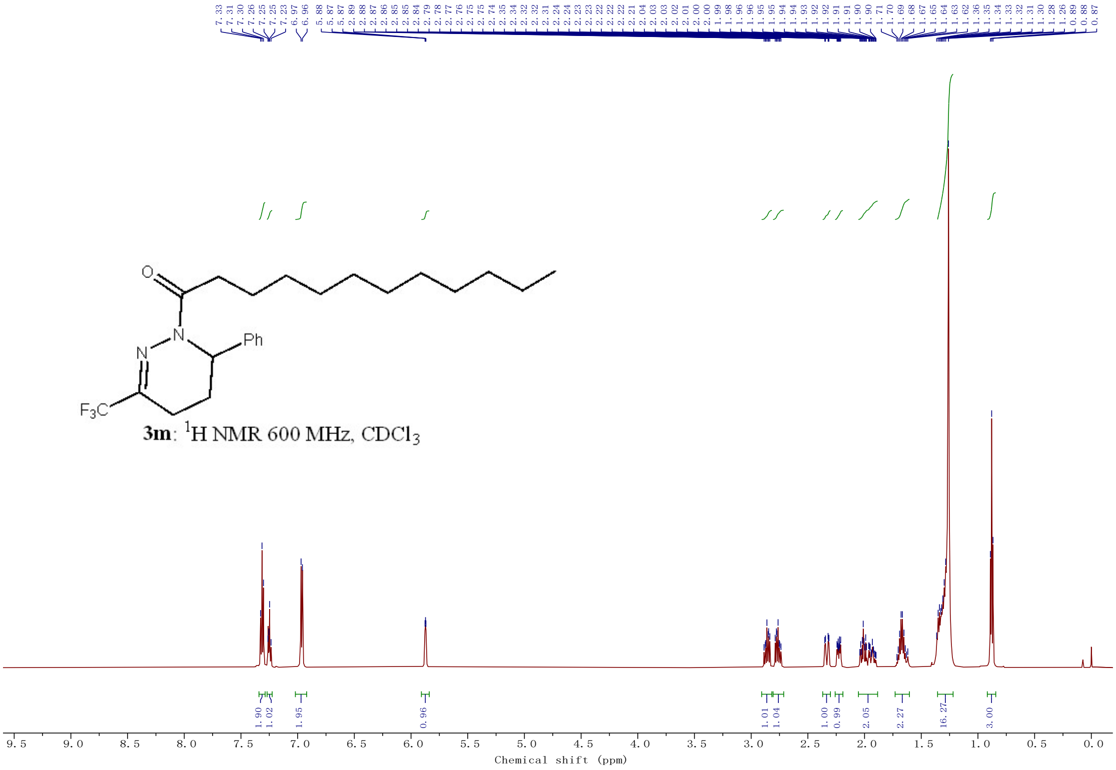

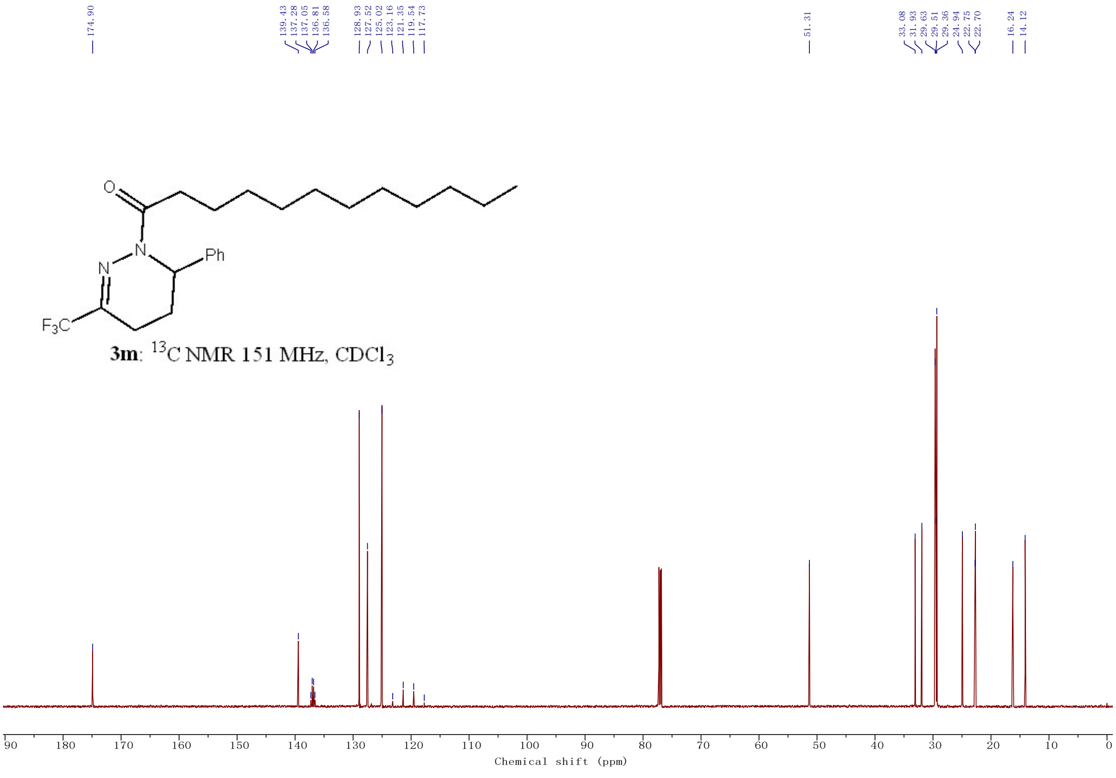

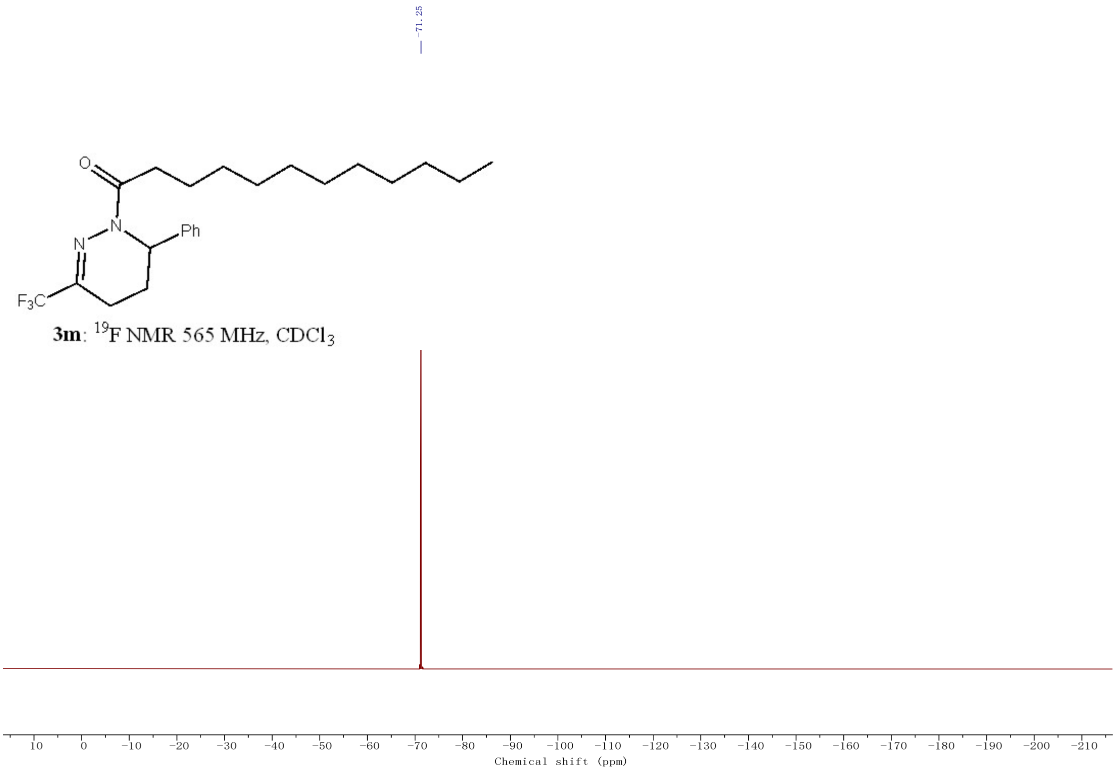

NMR copies of compound **3n:**

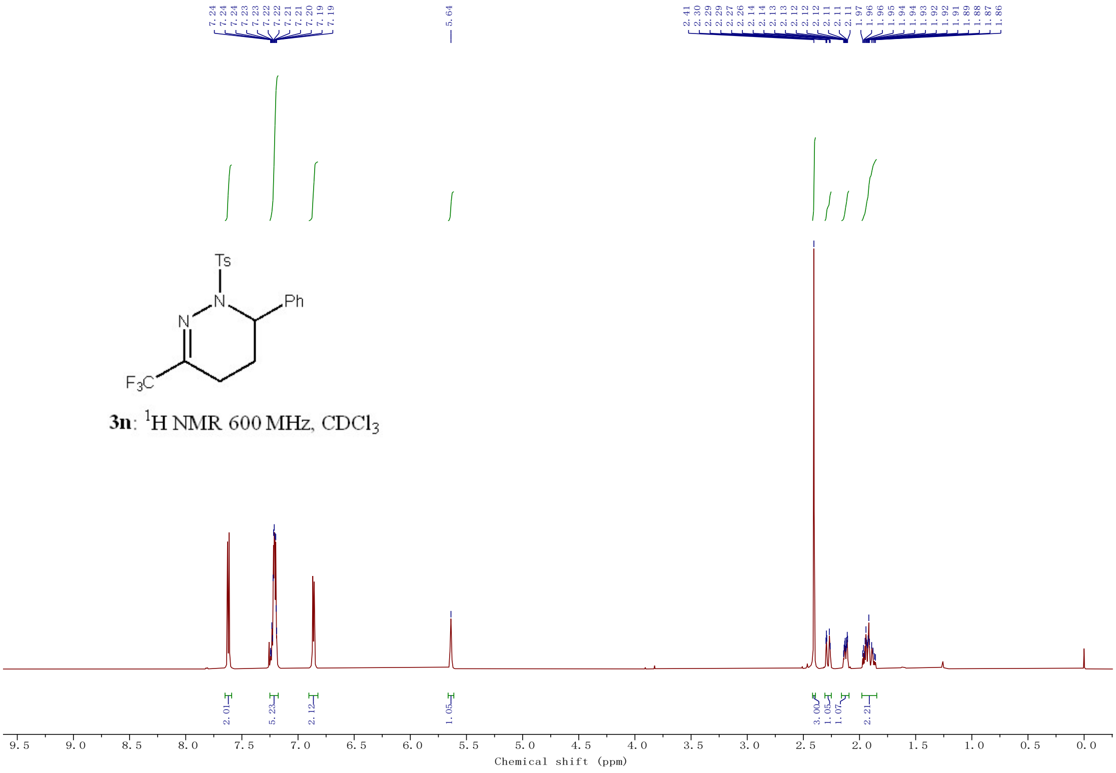

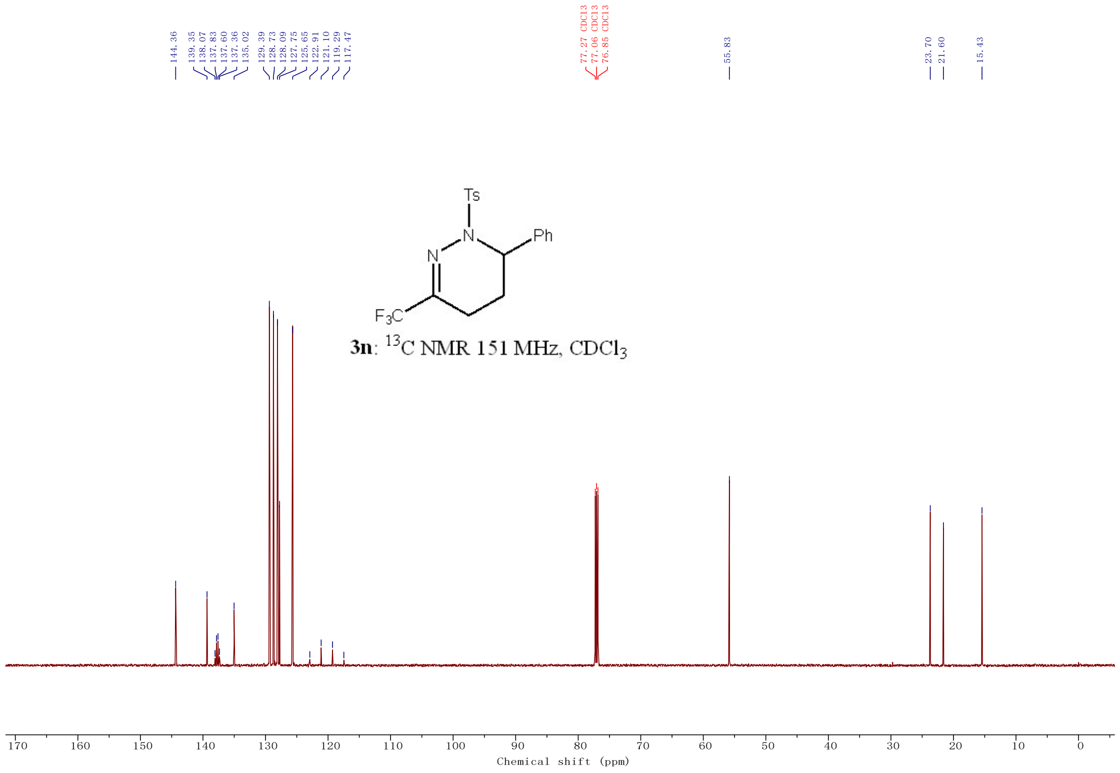

3n: ^{13}C NMR 151 MHz, $CDCl_3$

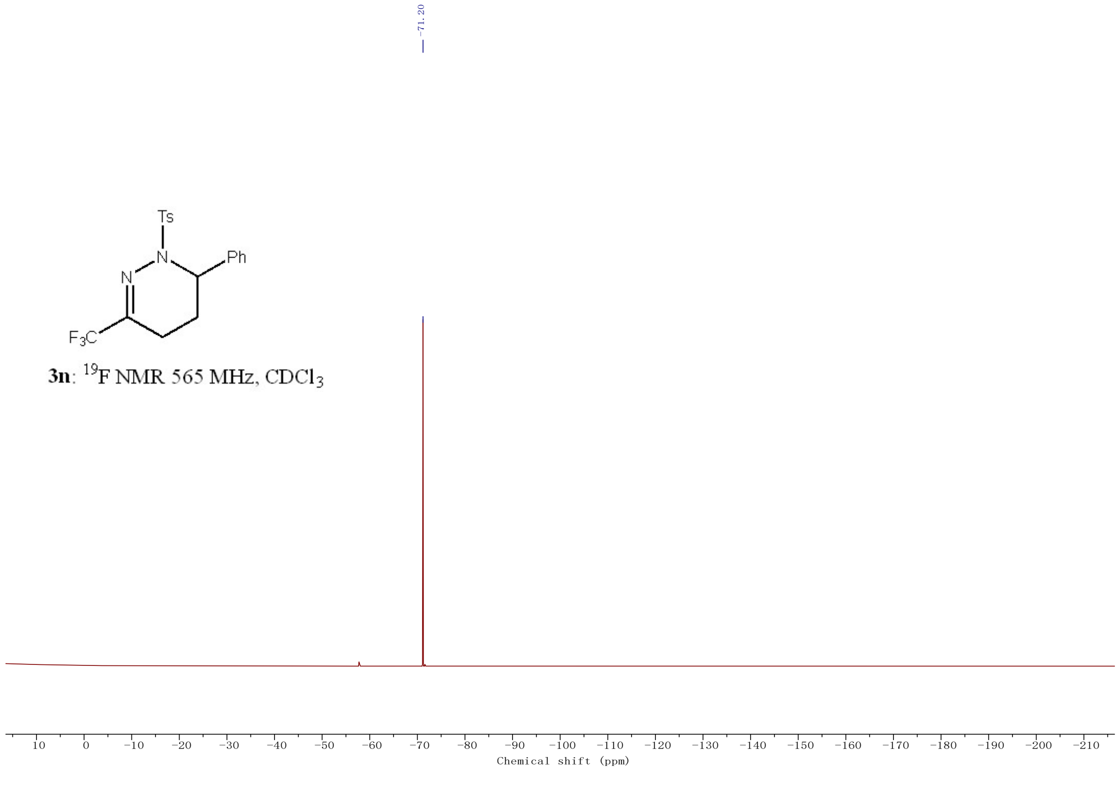

3n: ^{19}F NMR 565 MHz, $CDCl_3$

NMR copies of compound **3o:**

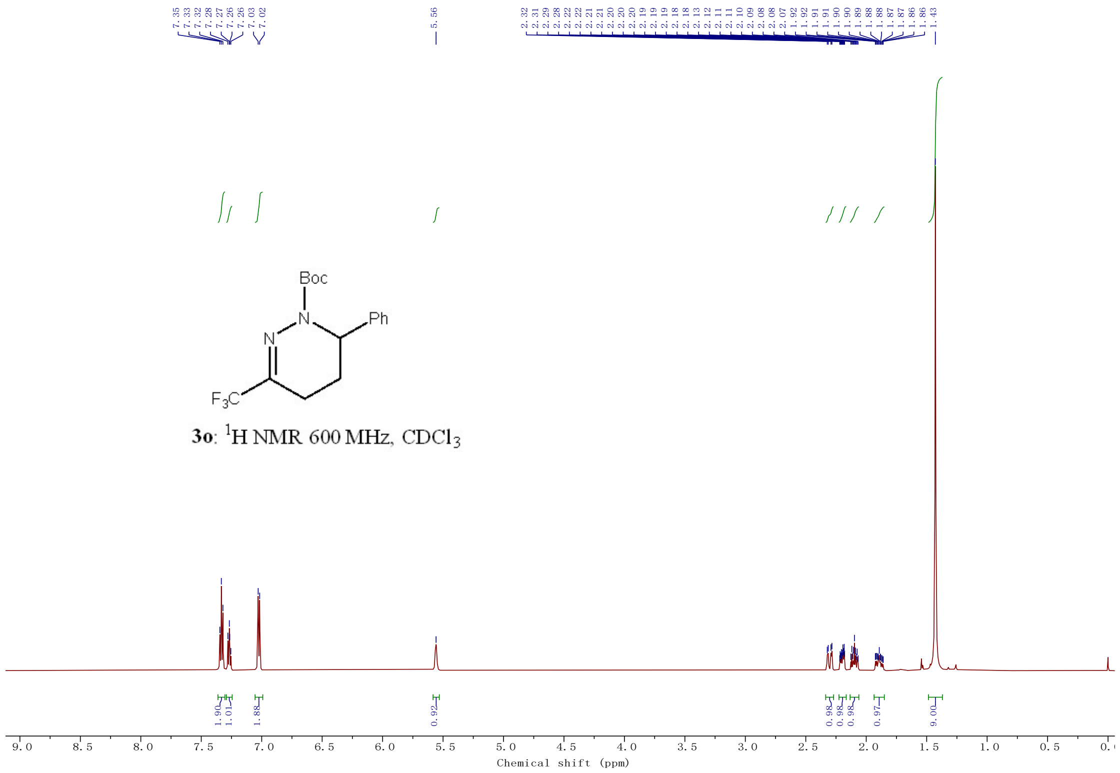

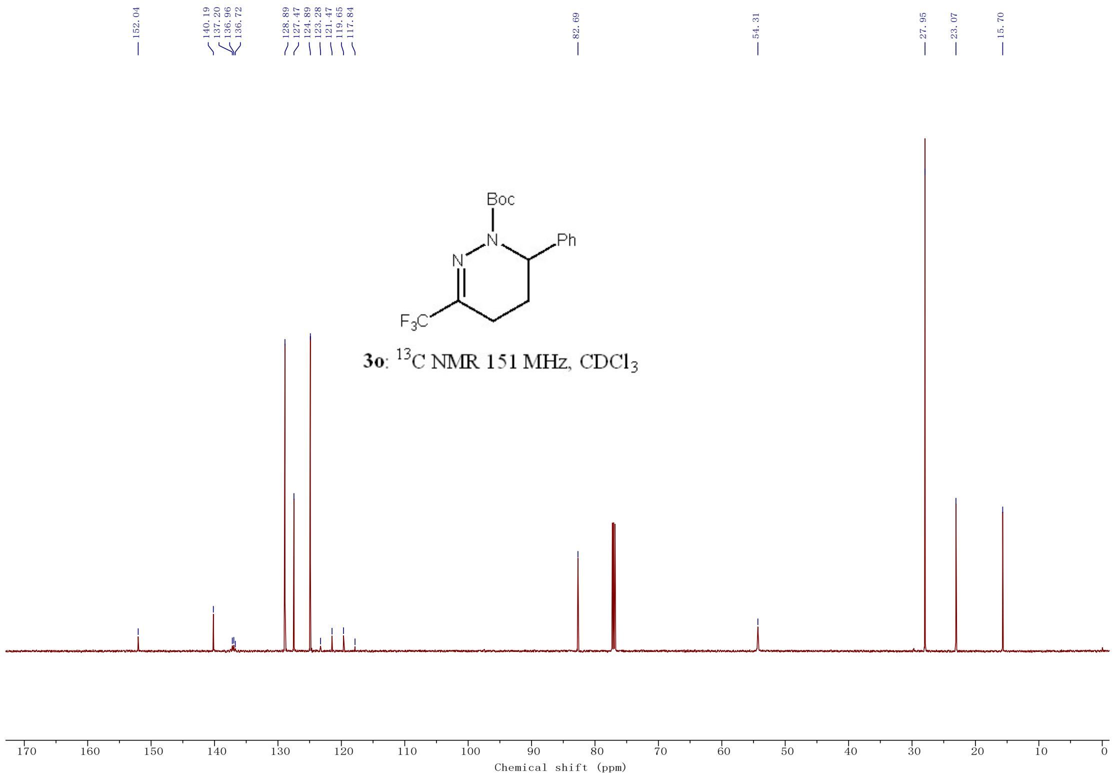

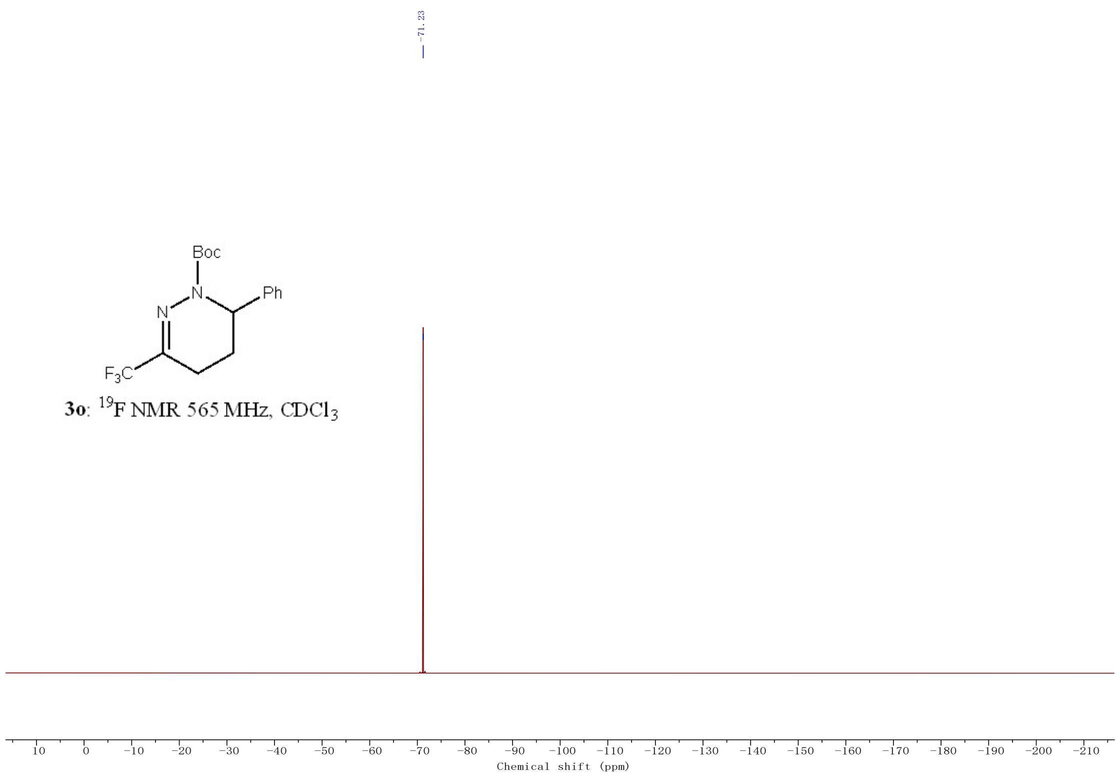

NMR copies of compound **4a:**

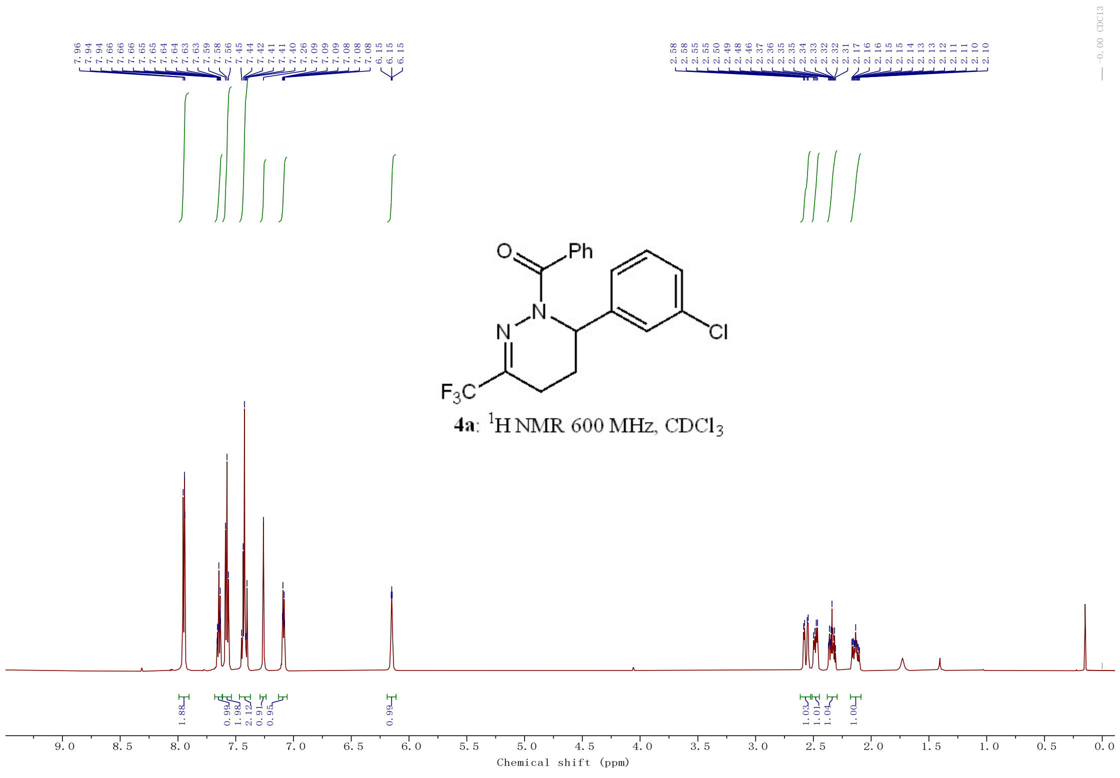

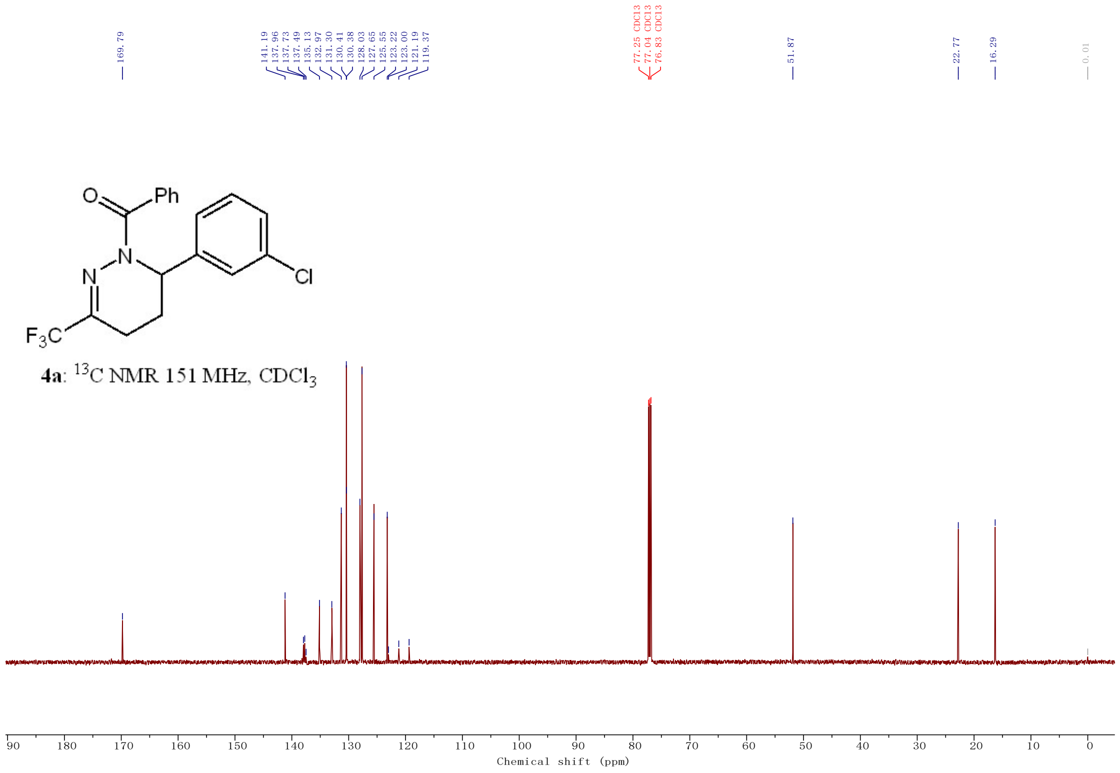
169.79
141.19
137.96
137.73
137.49
135.13
132.97
131.30
130.41
130.38
128.03
127.65
125.55
123.22
123.00
121.19
119.37
77.25 CDCl3
77.04 CDCl3
76.83 CDCl3
51.87
22.77
16.29
0.01
O
Ph
N
N
Cl
F_3C
4a: ^{13}C NMR 151 MHz, $CDCl_3$
Chemical shift (ppm)

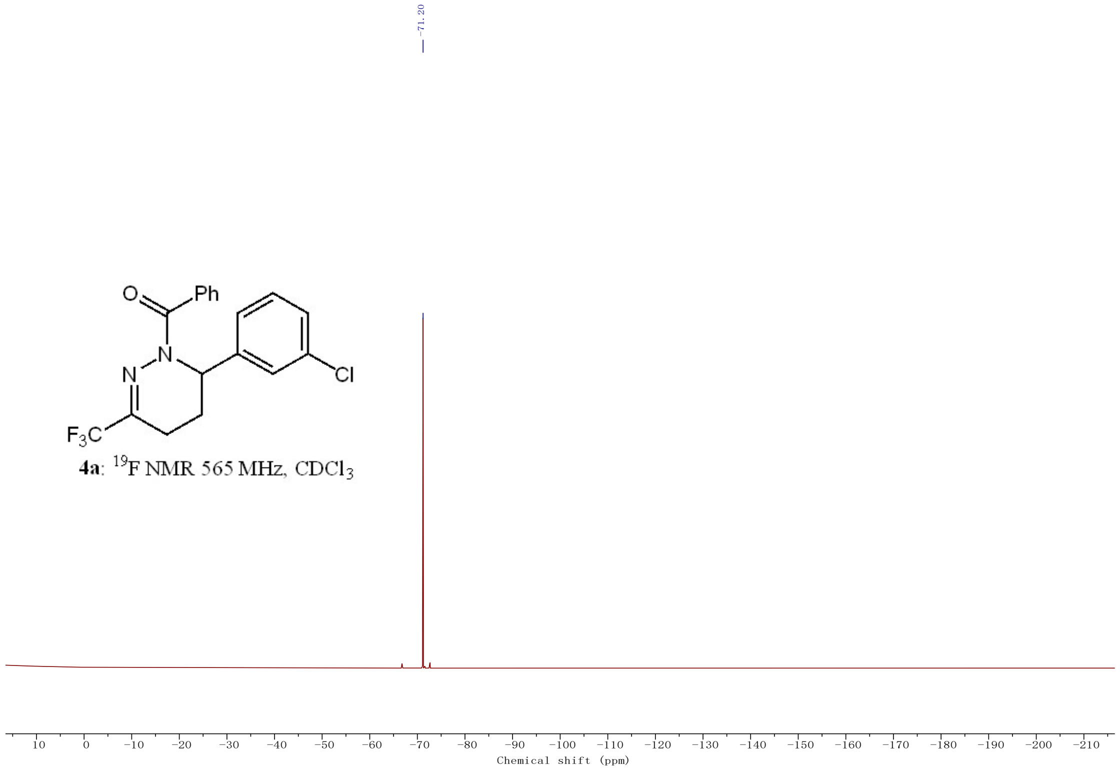
-71.20
O
Ph
N
N
Cl
F_3C
4a: ^{19}F NMR 565 MHz, $CDCl_3$
Chemical shift (ppm)

NMR copies of compound **4b:**

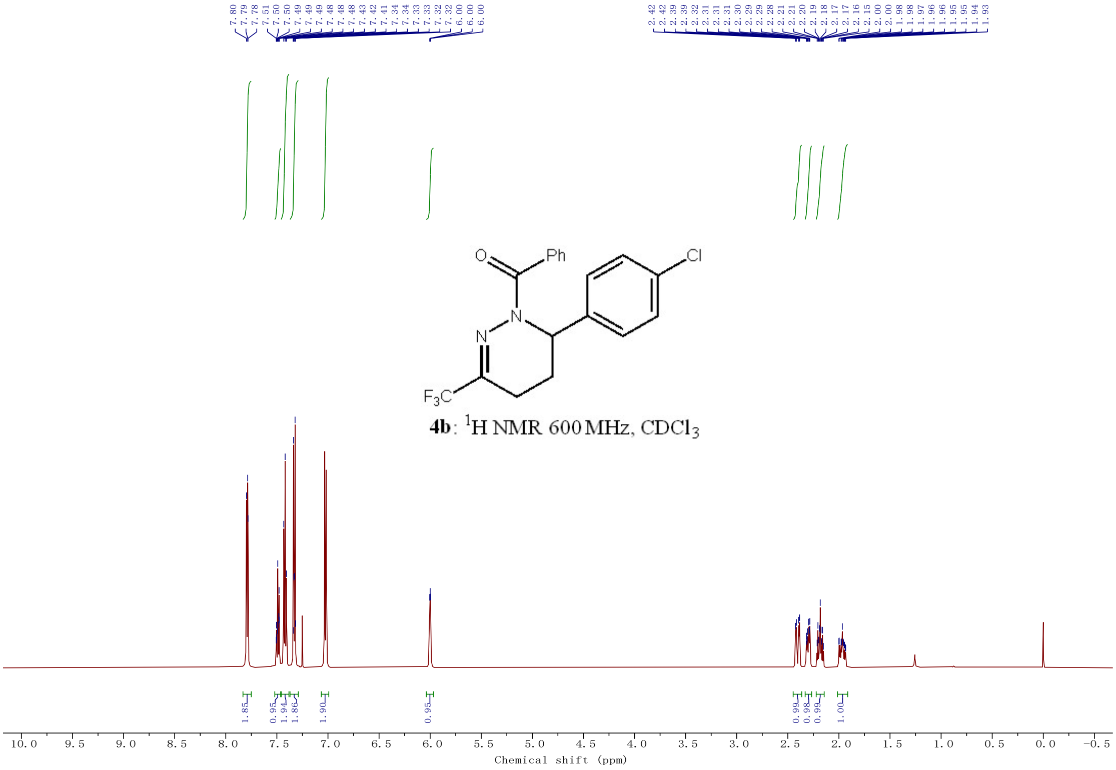

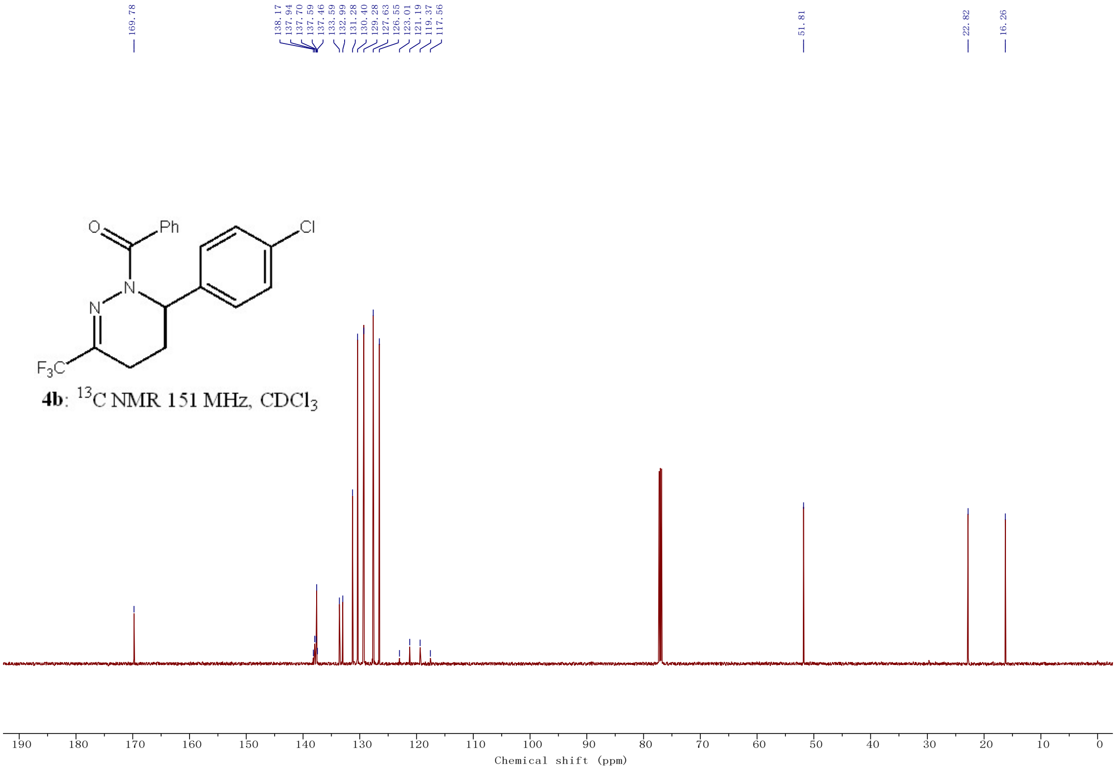

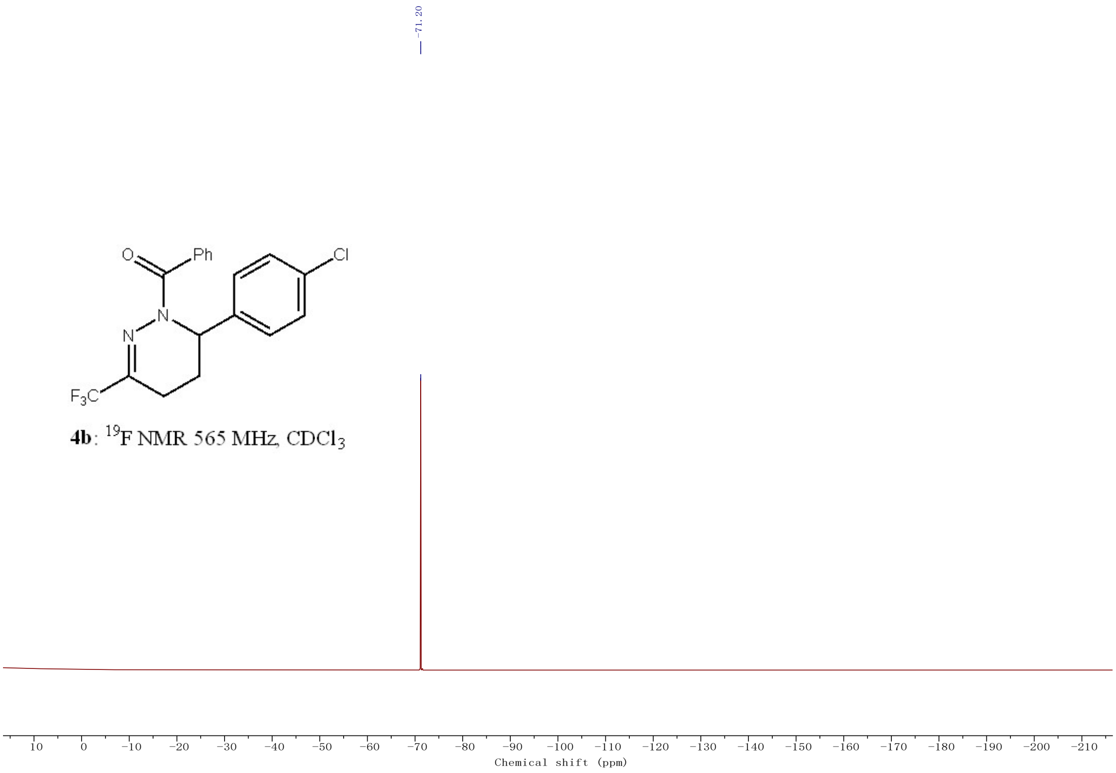

NMR copies of compound **4c:**

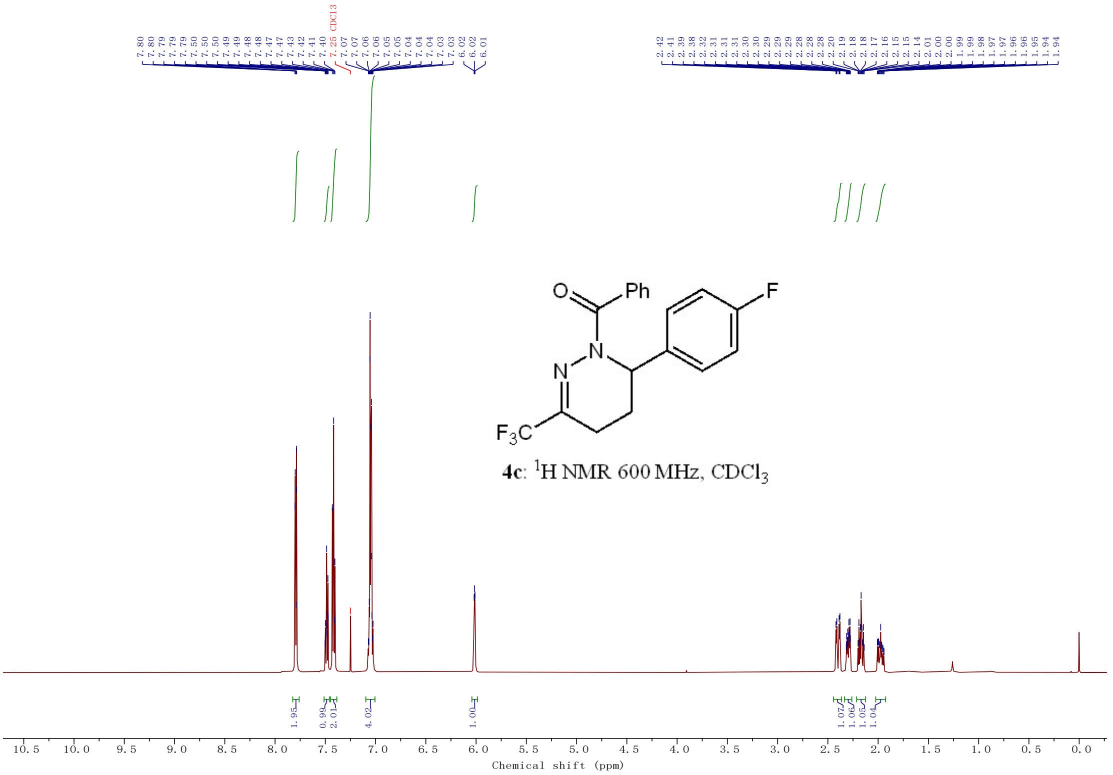

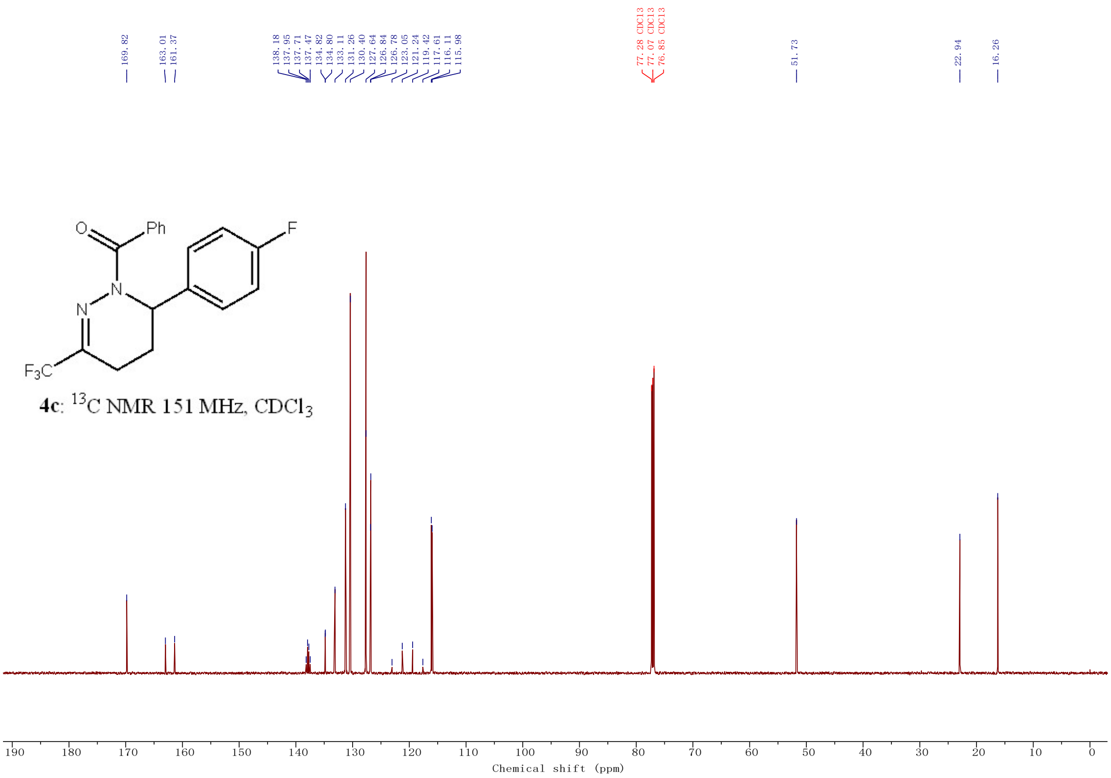

4c: ^{13}C NMR 151 MHz, $CDCl_3$

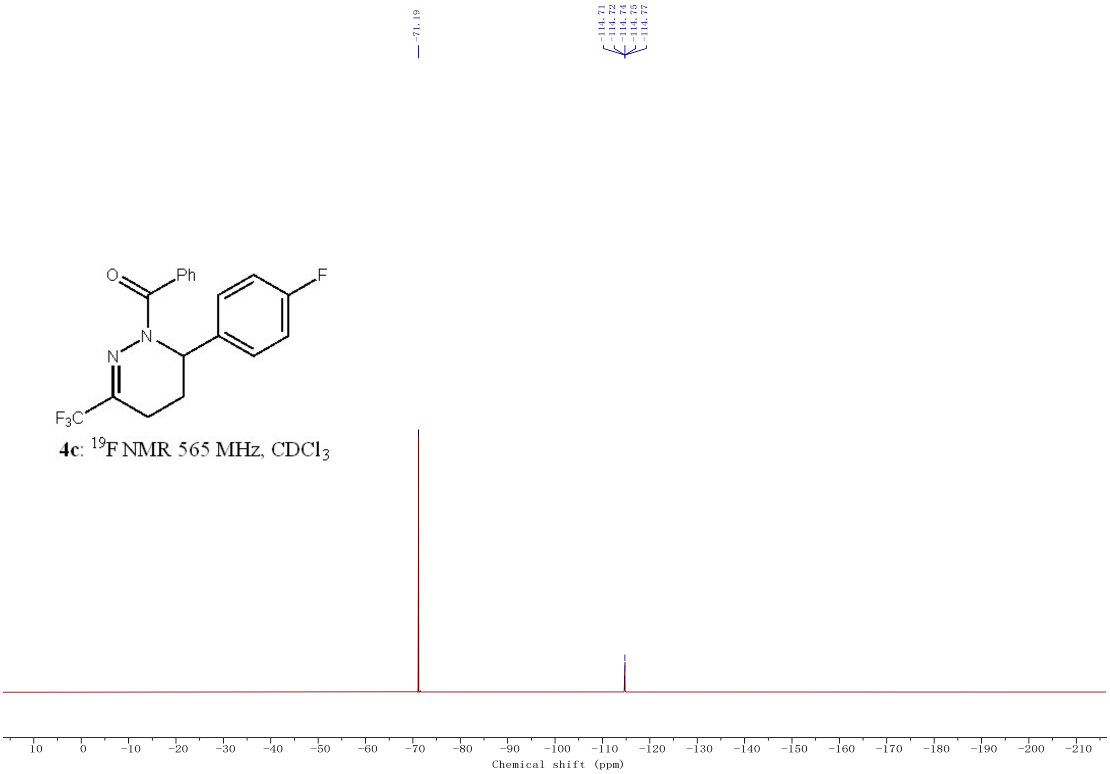

4c: ^{19}F NMR 565 MHz, $CDCl_3$

NMR copies of compound **4d:**

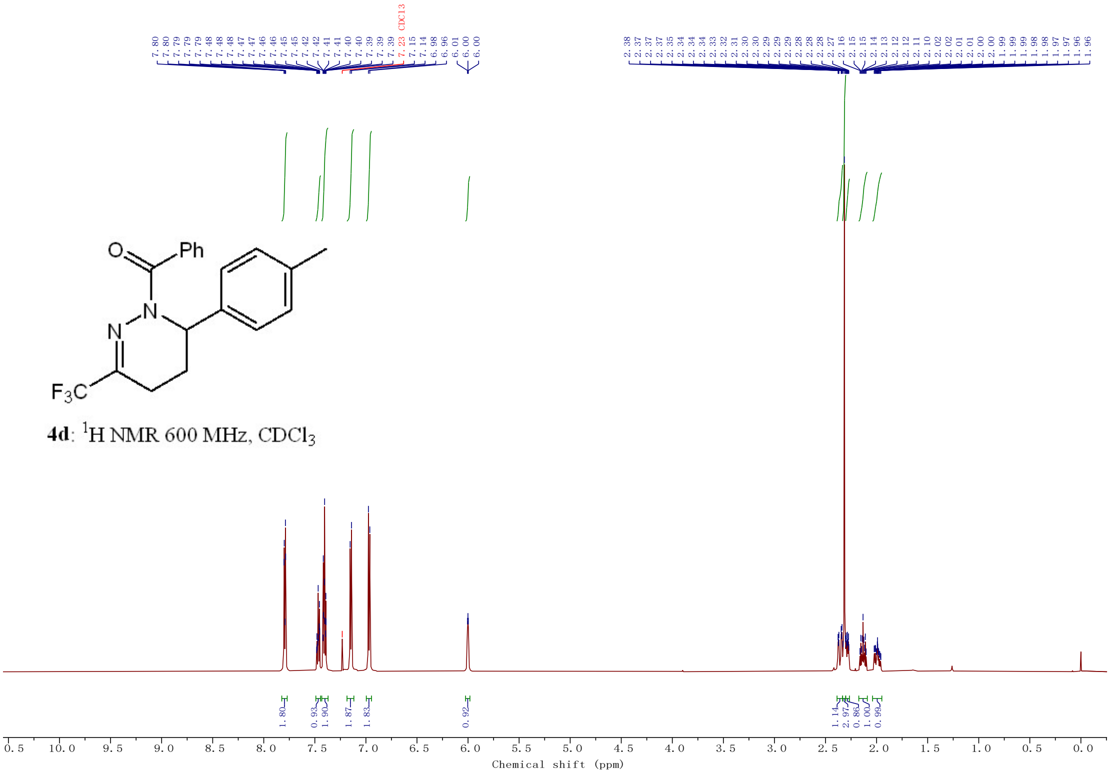

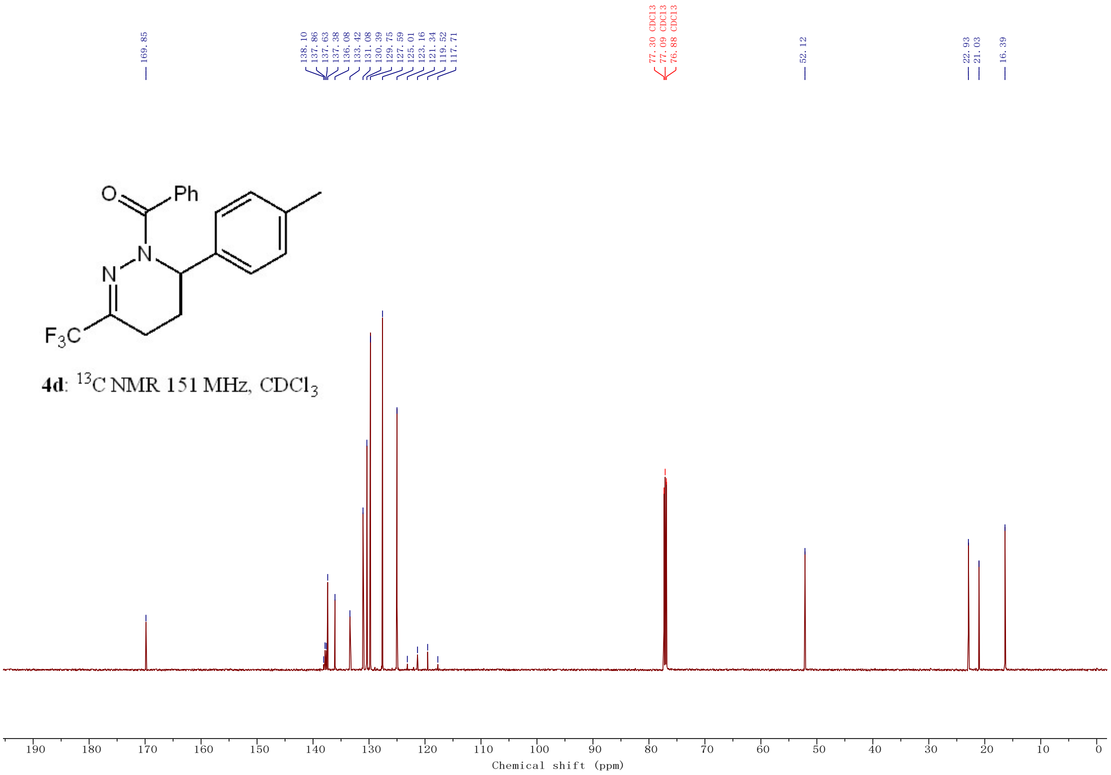

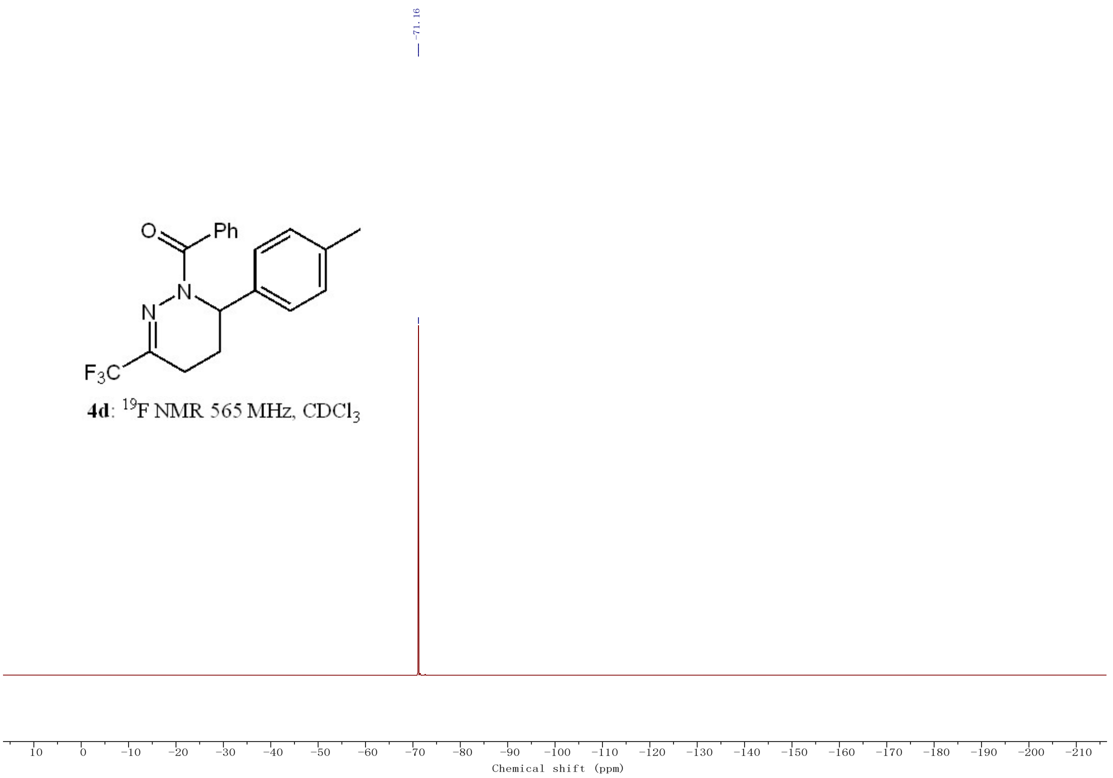

NMR copies of compound **4e:**

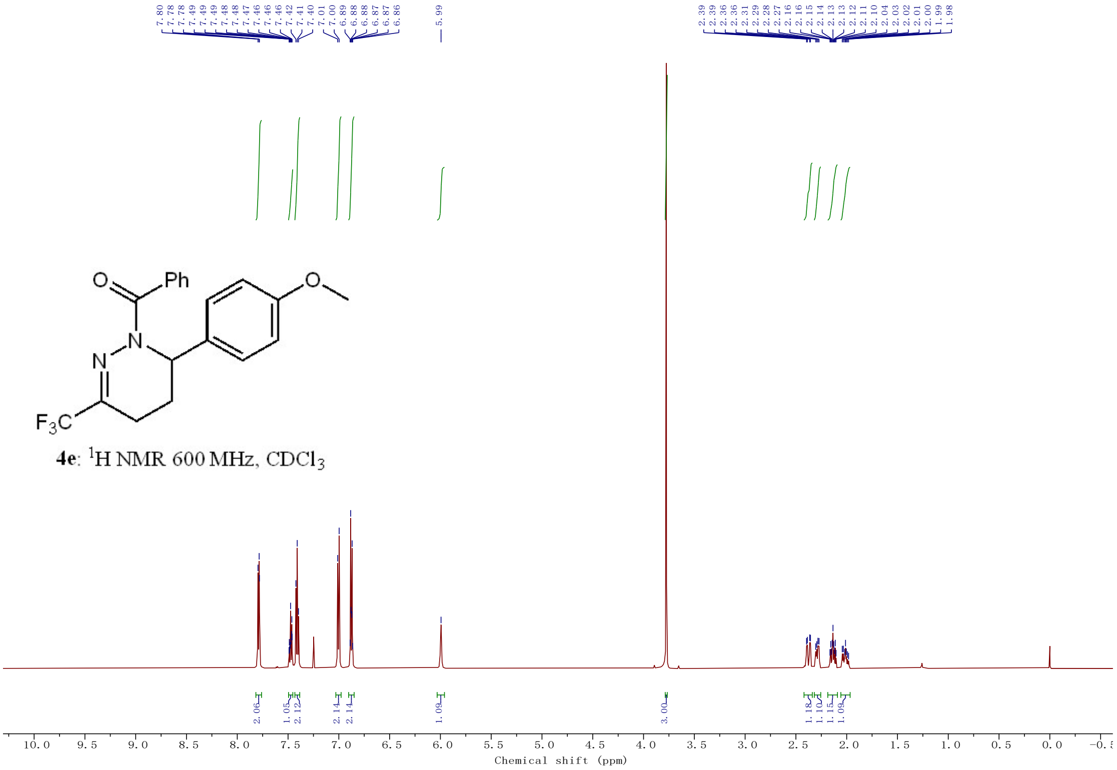

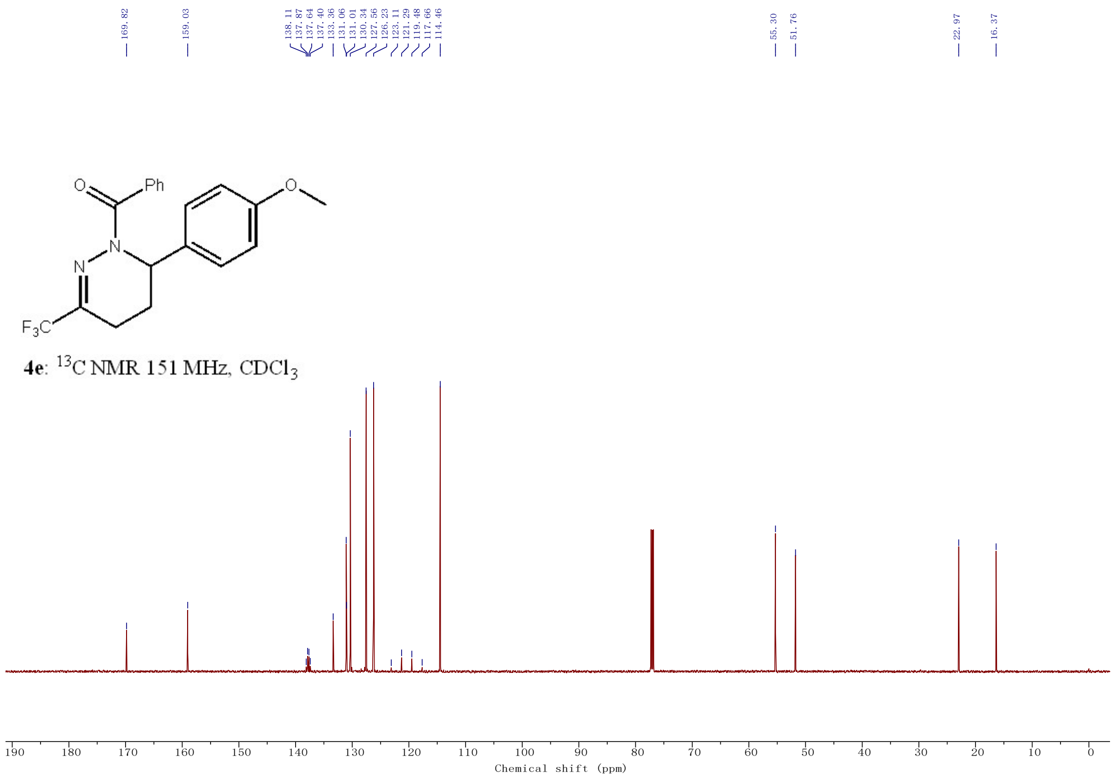

4e: ^{13}C NMR 151 MHz, $CDCl_3$

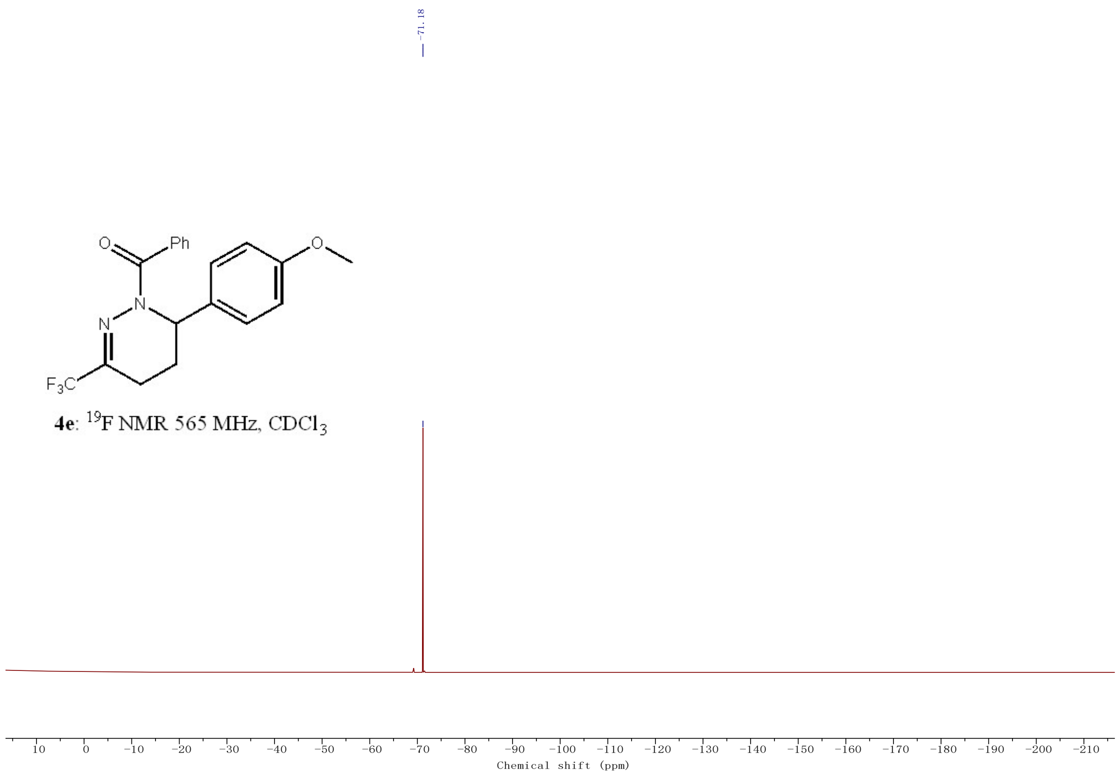

4e: ^{19}F NMR 565 MHz, $CDCl_3$

NMR copies of compound **4f:**

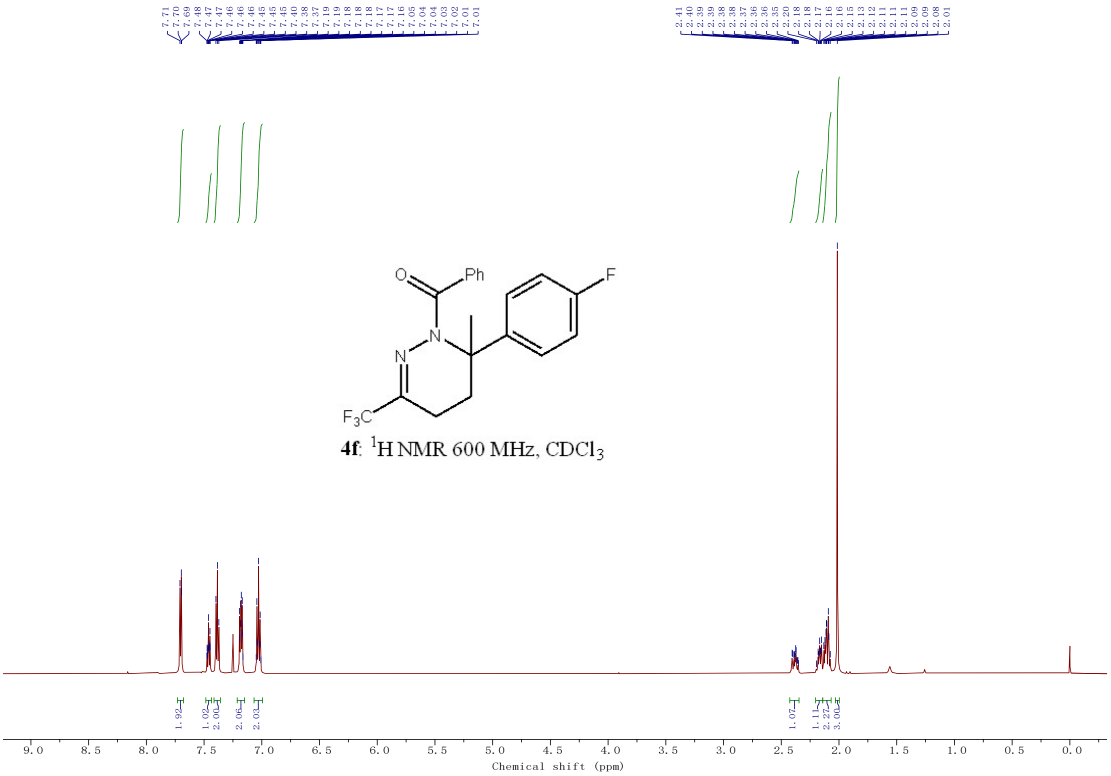

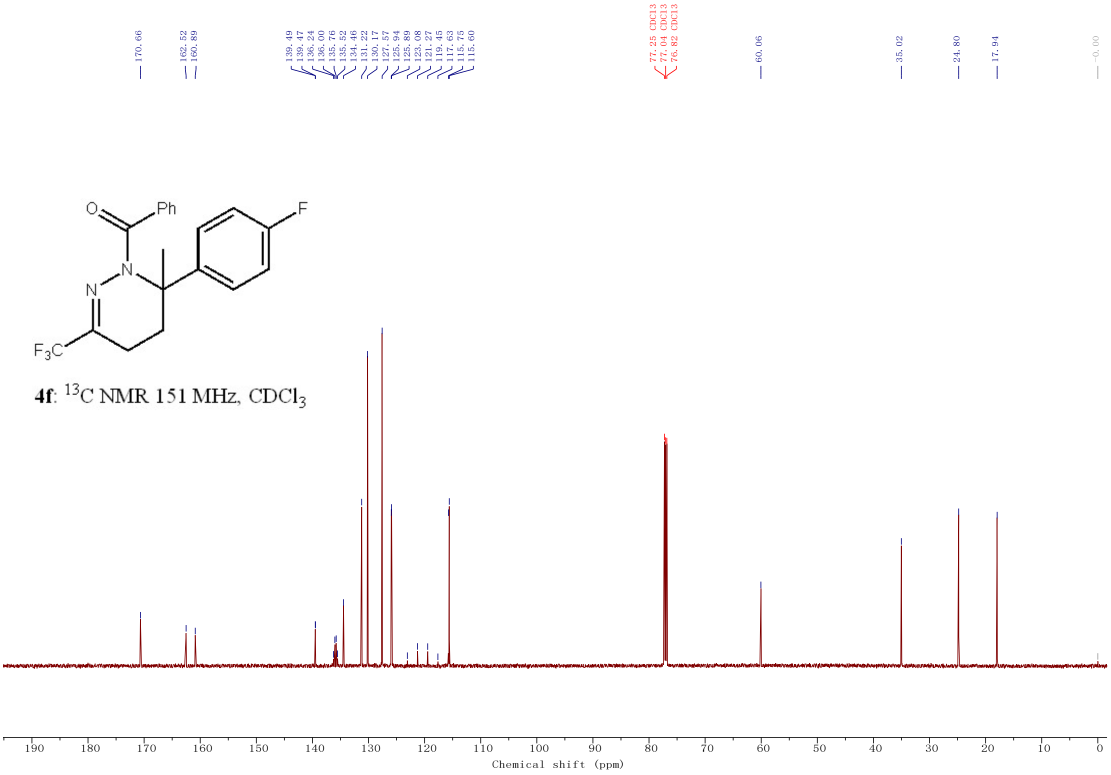

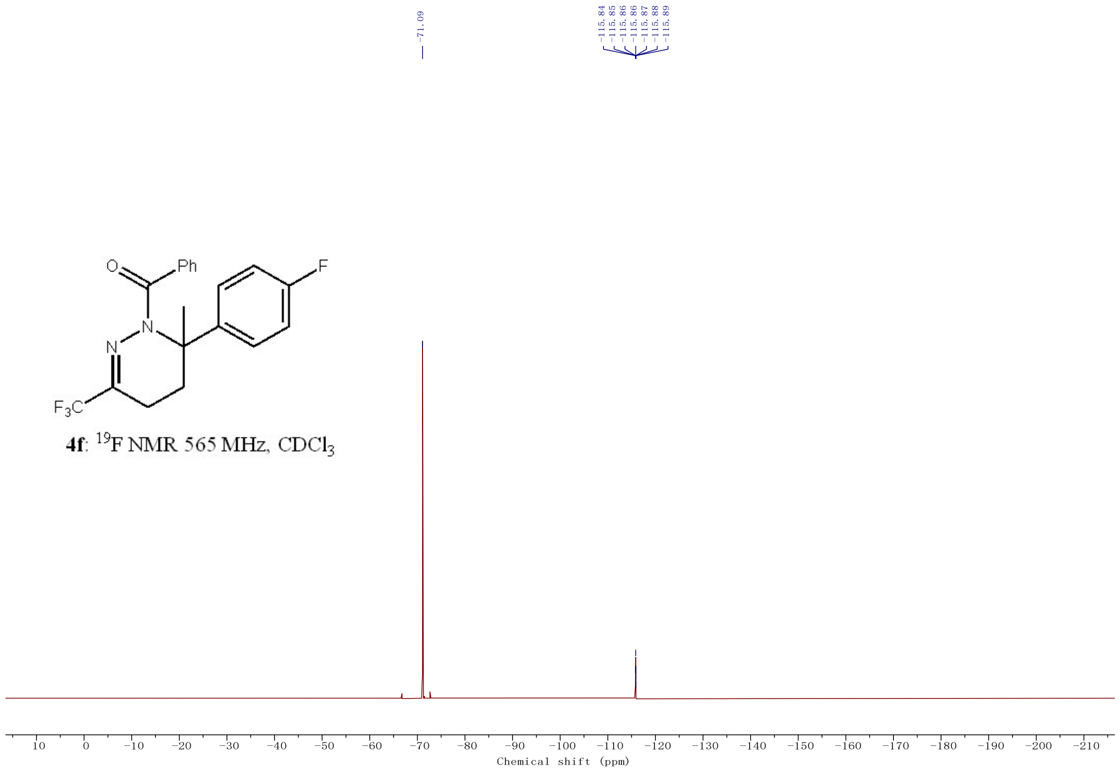

NMR copies of compound **4g:**

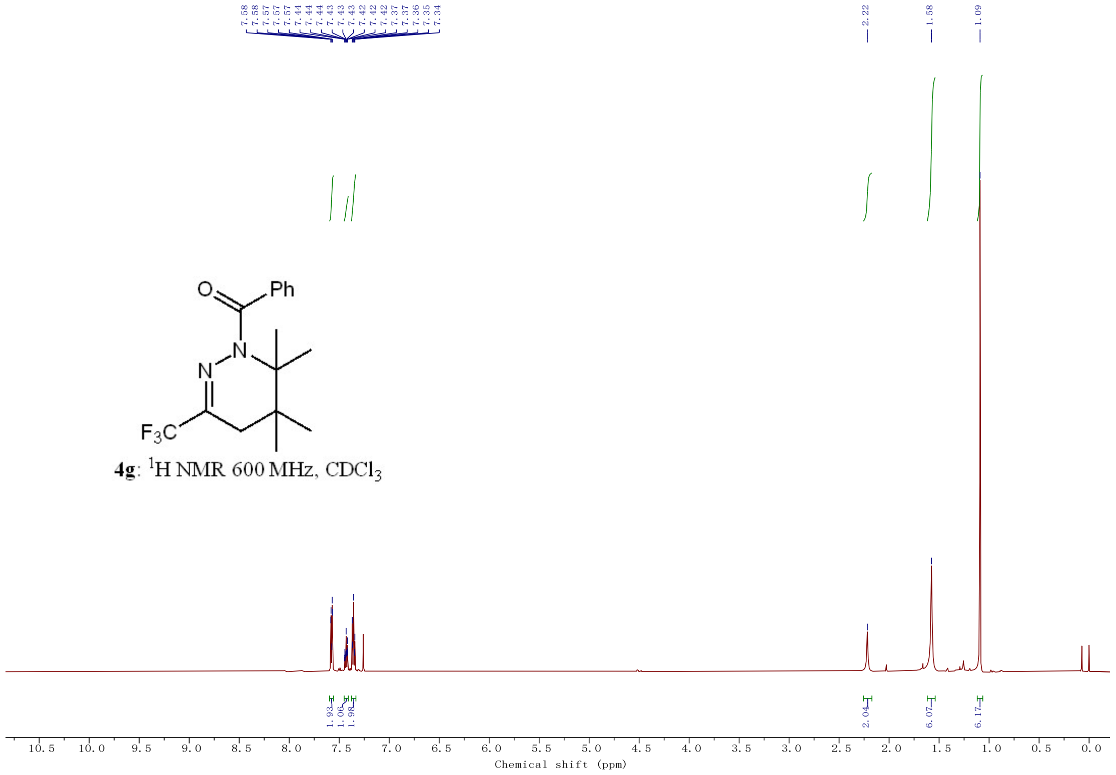

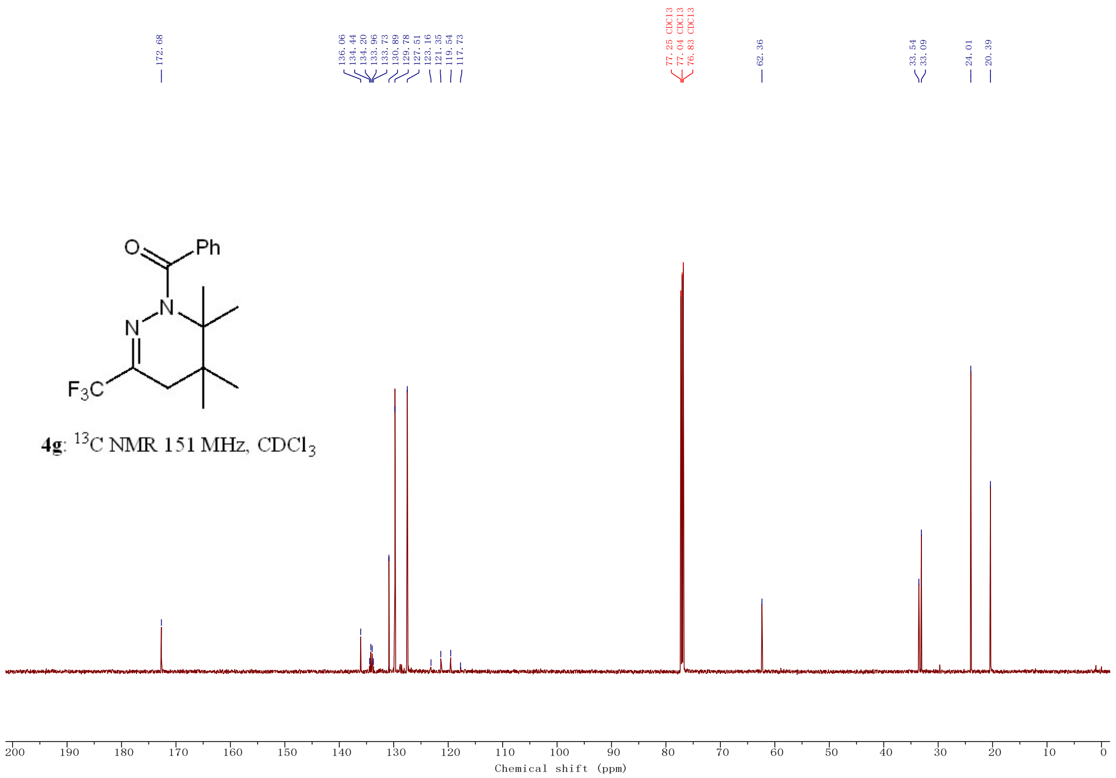
172.68
136.06
134.44
134.20
133.96
133.73
130.89
129.78
127.51
123.16
121.35
119.54
117.73
77.25 CDCl3
77.04 CDCl3
76.83 CDCl3
62.36
33.54
33.09
24.01
20.39
4g: 13C NMR 151 MHz, CDCl3
Chemical shift (ppm)

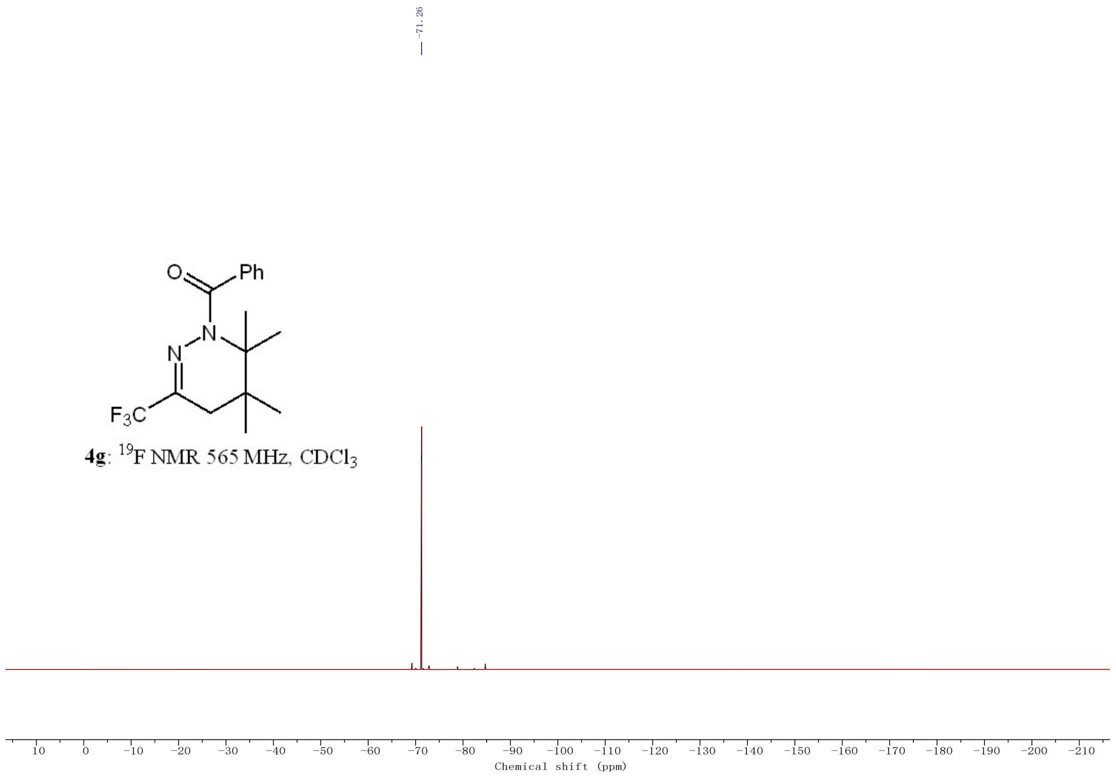
-71.26
4g: 19F NMR 565 MHz, CDCl3
Chemical shift (ppm)

NMR copies of compound **5a:**

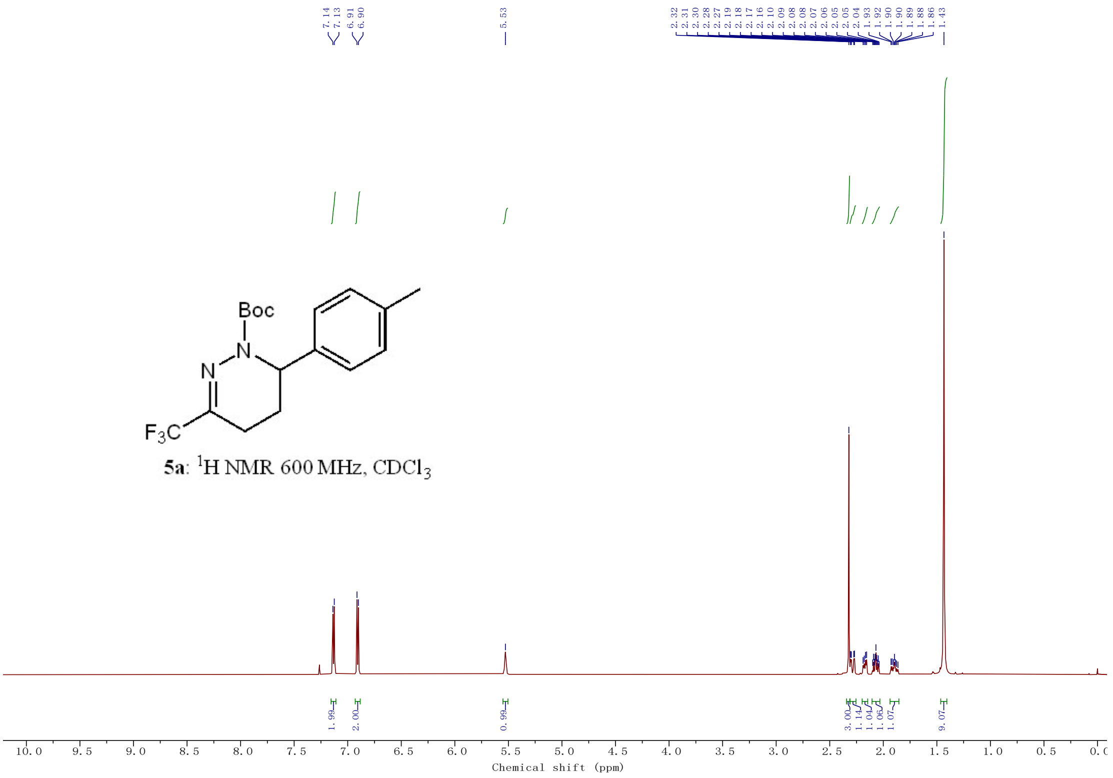

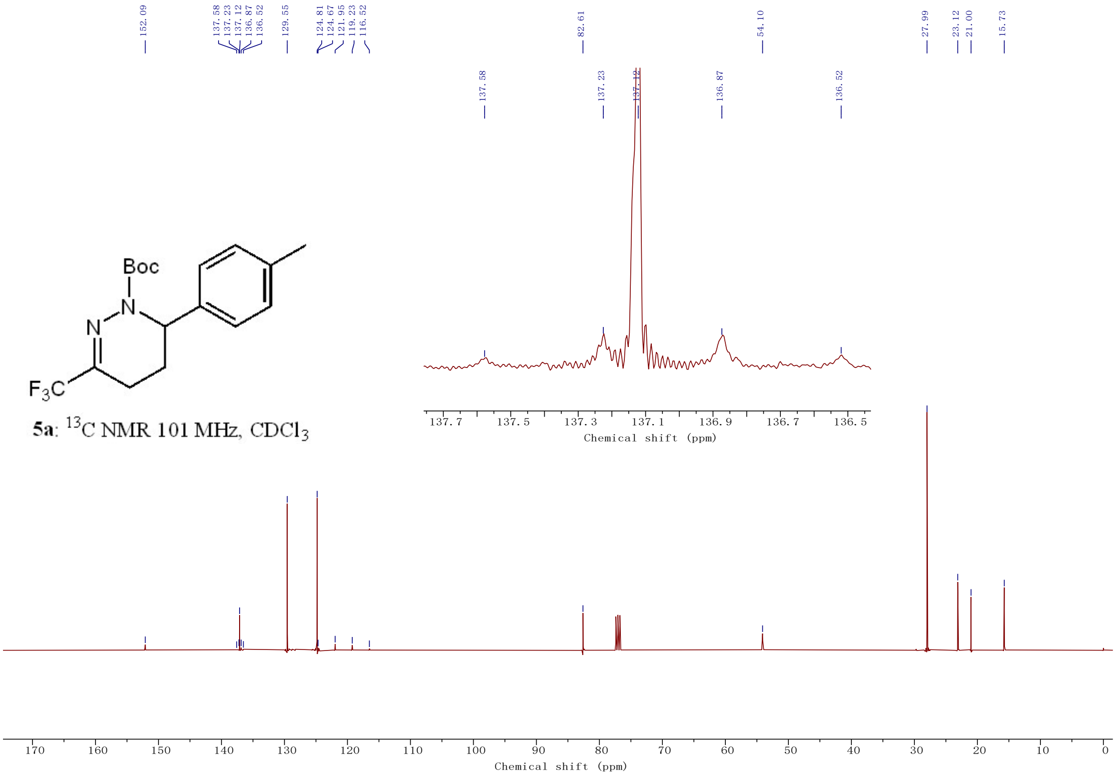

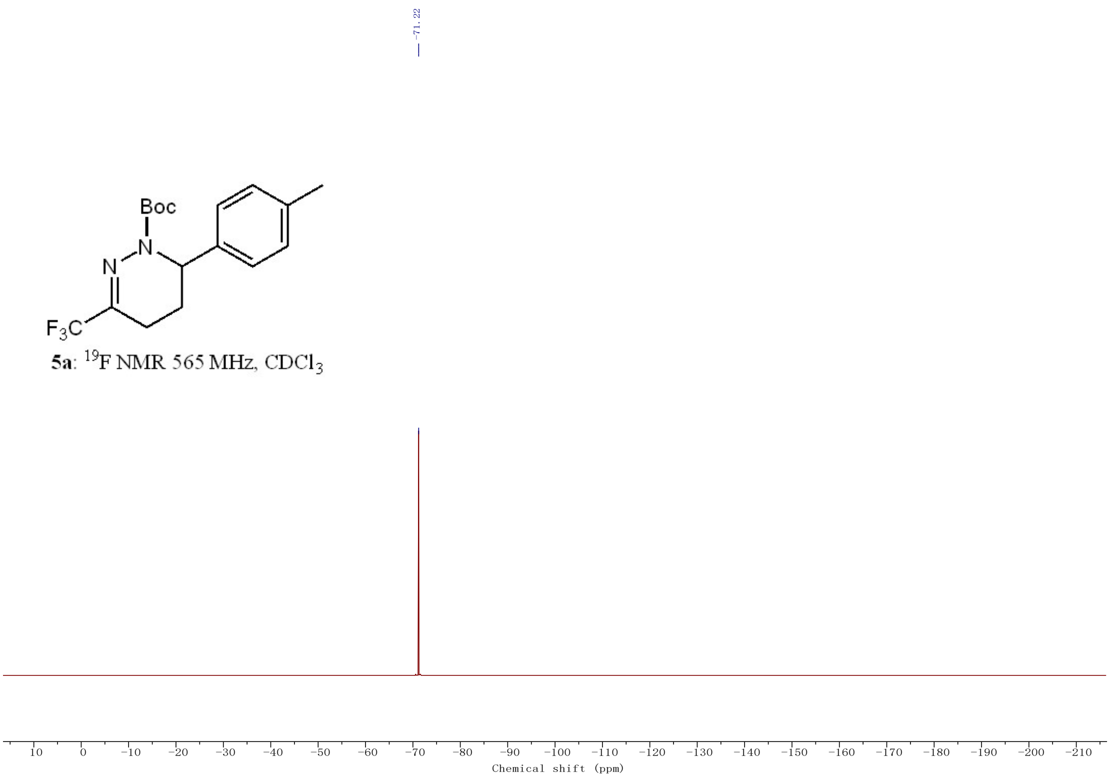

NMR copies of compound **5b:**

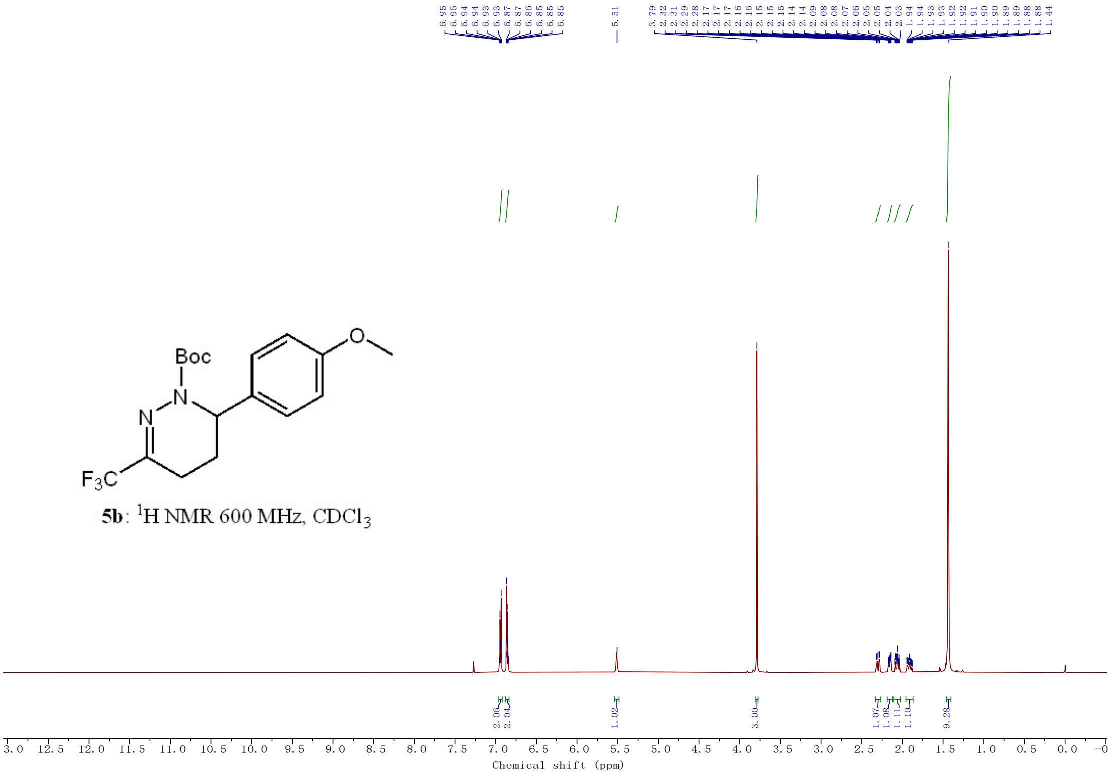

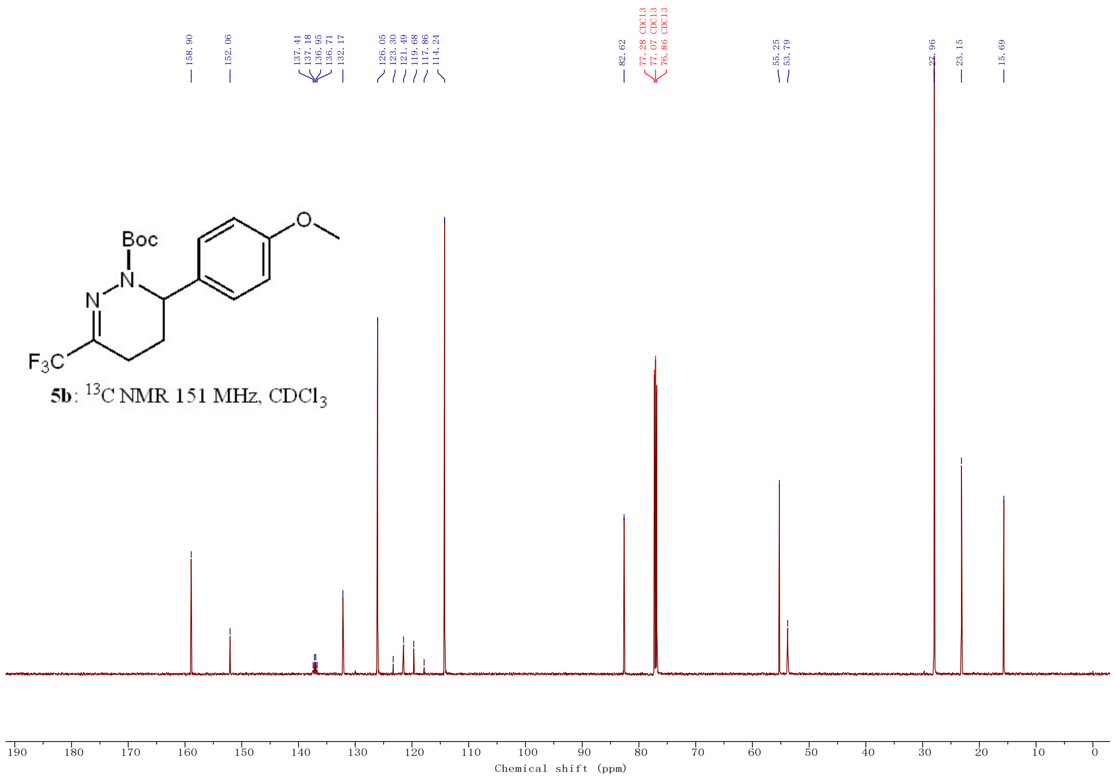
158.90
152.06
137.41
137.18
136.95
136.71
132.17
126.05
123.30
121.49
119.68
117.86
114.24
82.62
77.28 CDCl3
77.07 CDCl3
76.86 CDCl3
55.25
53.79
27.96
23.15
15.69
Boc
N
N
O
F3C
5b: 13C NMR 151 MHz, CDCl3
Chemical shift (ppm)

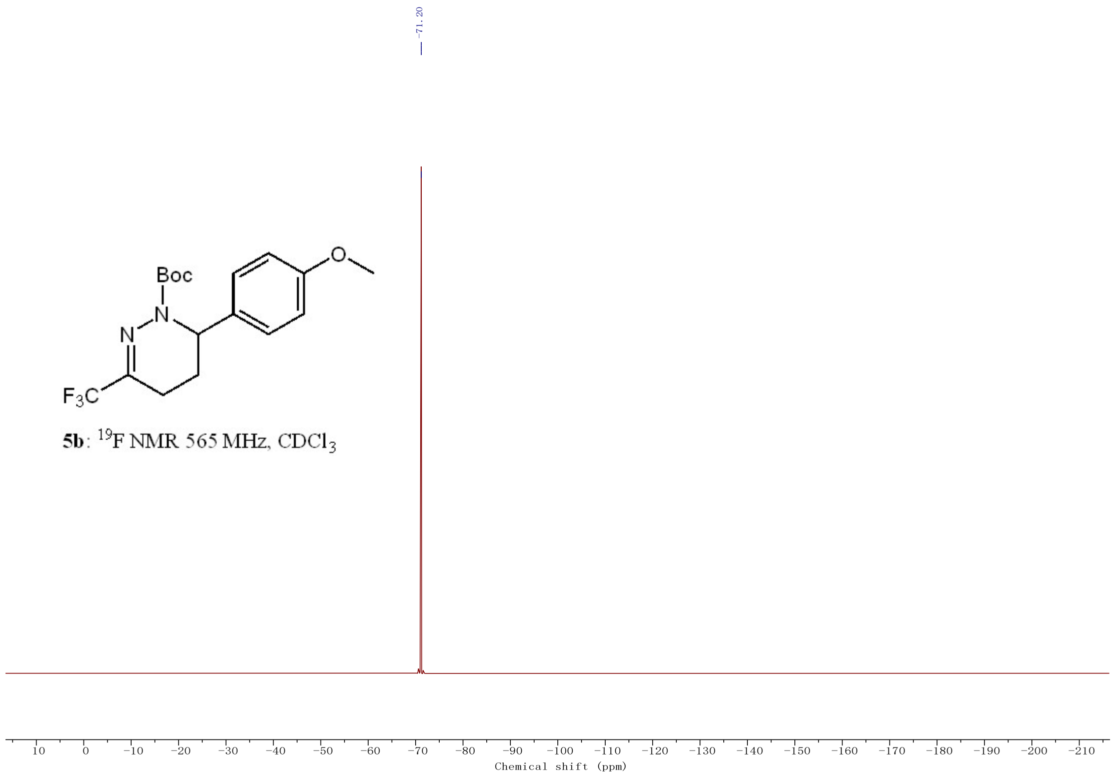
-71.20
Boc
N
N
O
F3C
5b: 19F NMR 565 MHz, CDCl3
Chemical shift (ppm)

NMR copies of compound **5c:**

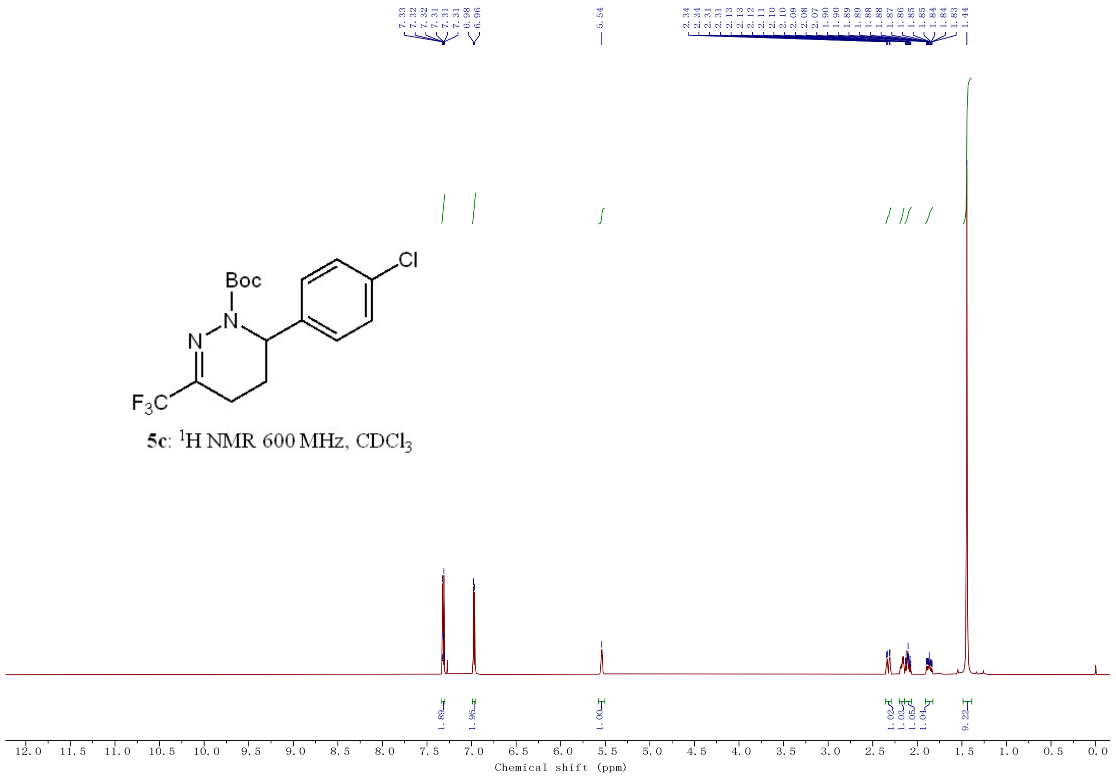

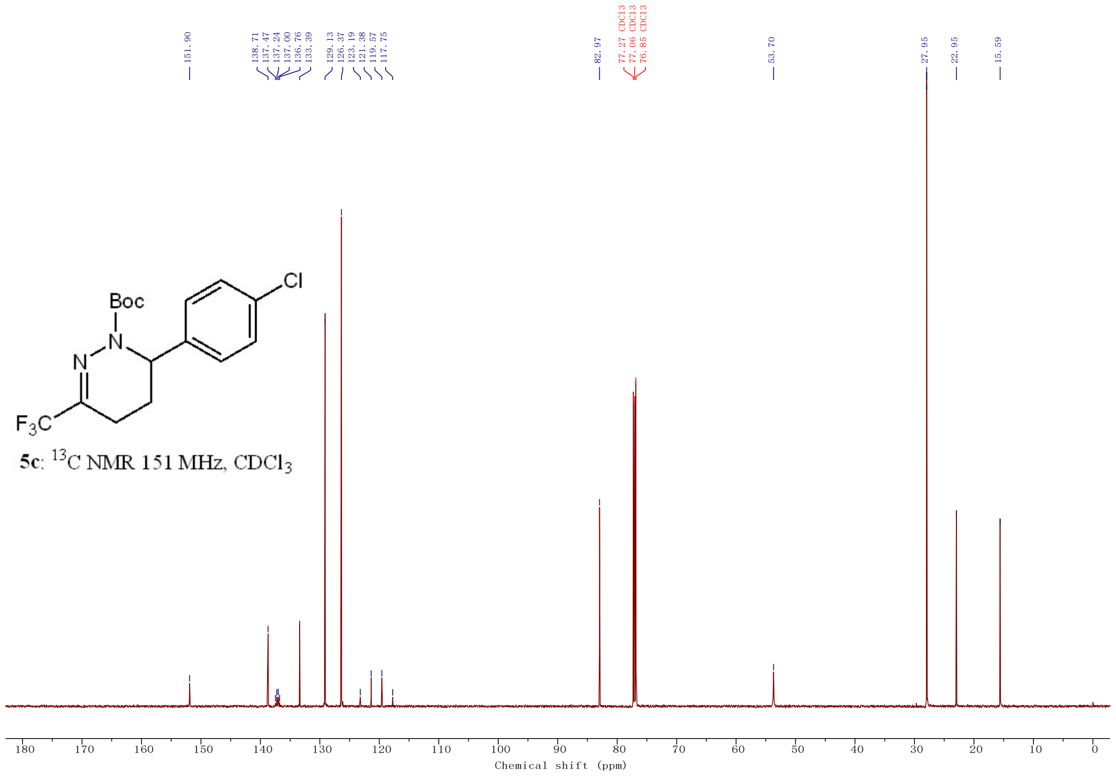

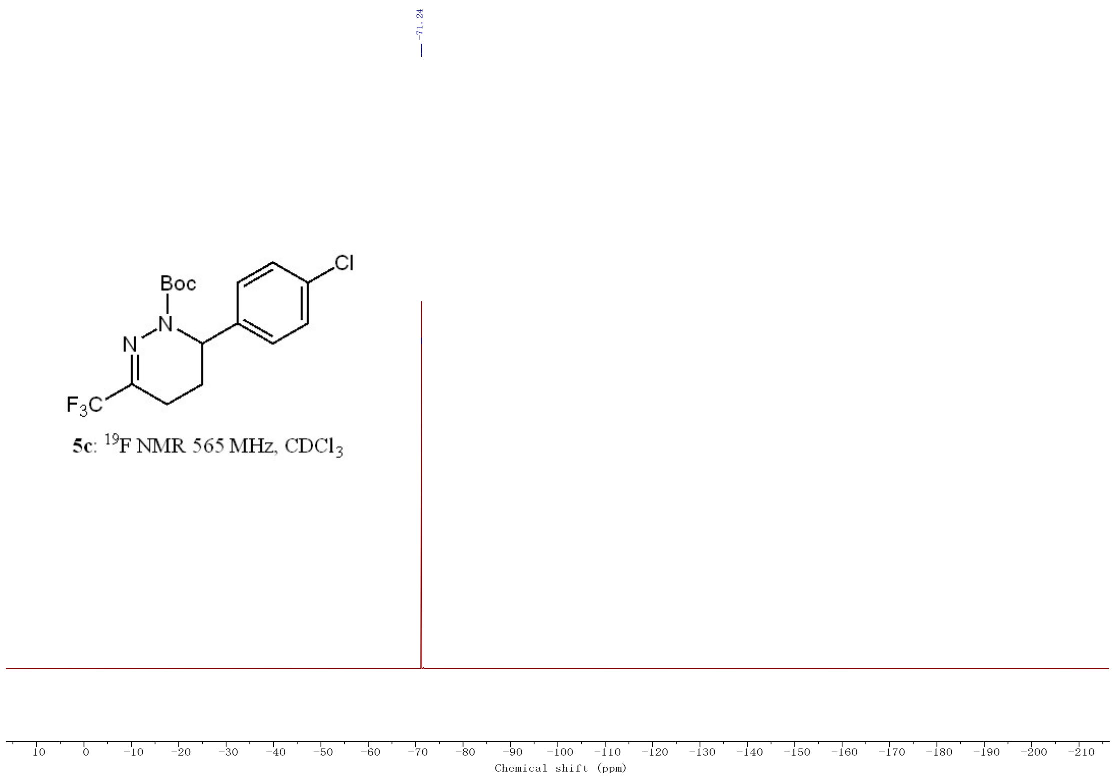

NMR copies of compound **5d:**

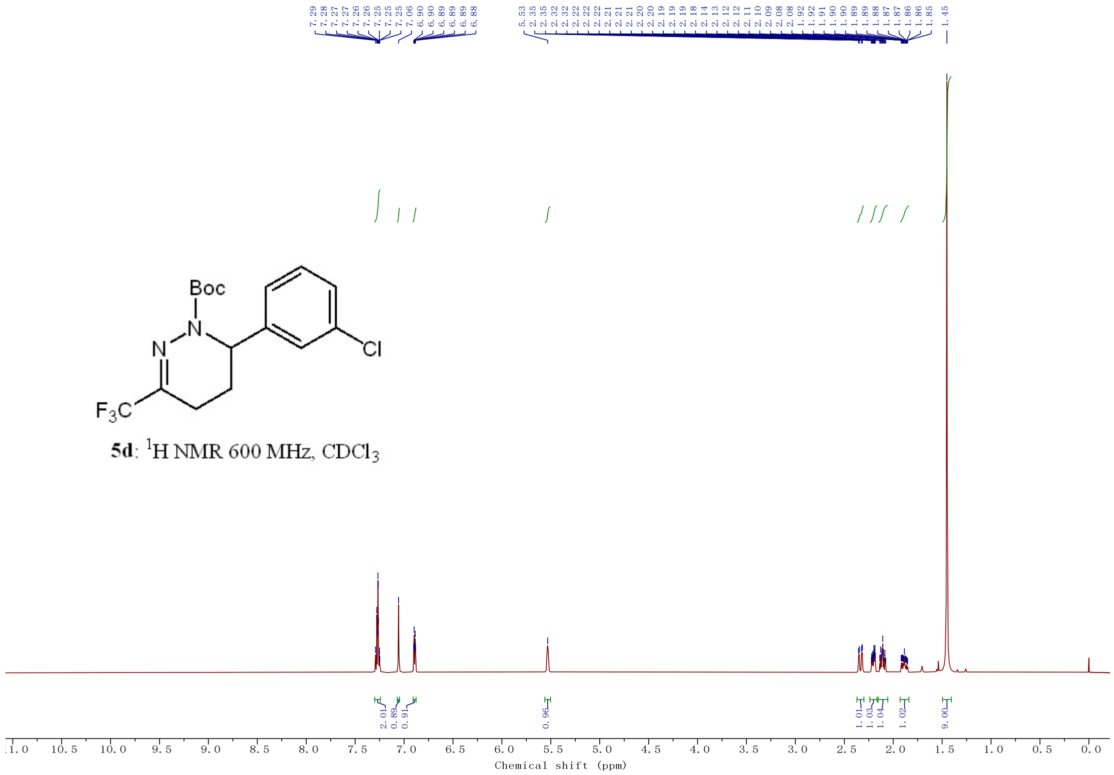

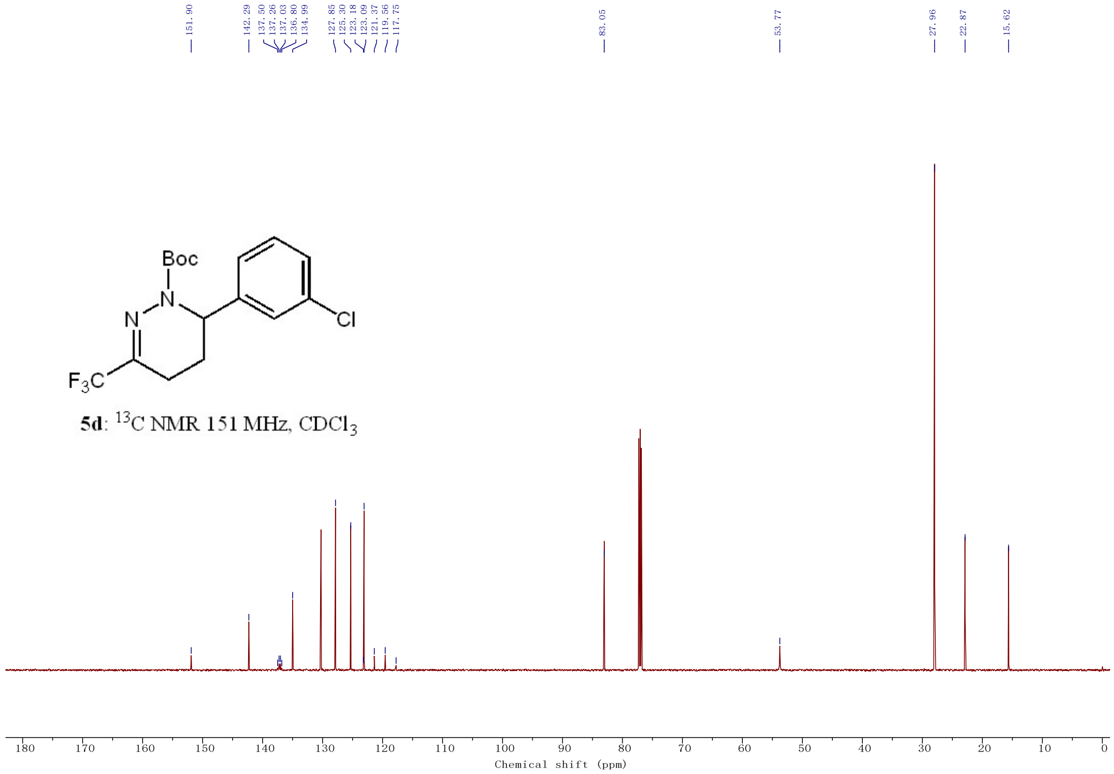

5d: ^{13}C NMR 151 MHz, $CDCl_3$

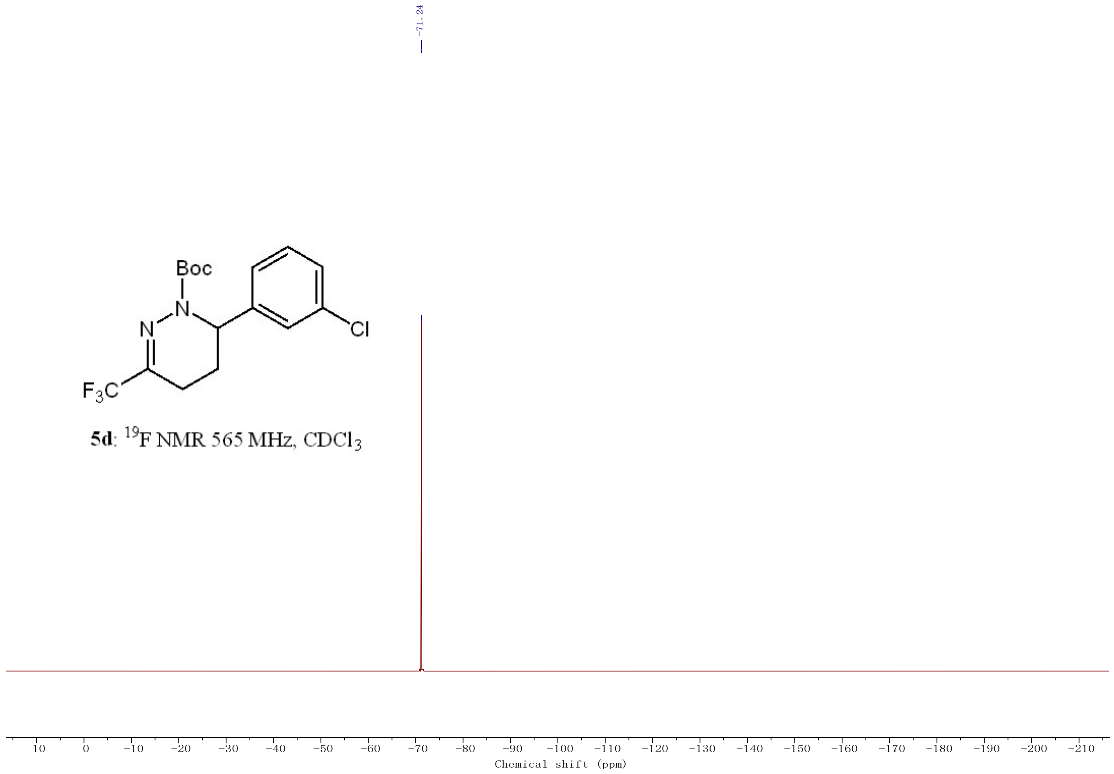

5d: ^{19}F NMR 565 MHz, $CDCl_3$
